# Lactoferrin-derived Peptides Active towards Influenza: Identification of Three Potent Tetrapeptide Inhibitors

**DOI:** 10.1038/s41598-017-10492-x

**Published:** 2017-09-06

**Authors:** Maria Carmina Scala, Marina Sala, Agostina Pietrantoni, Antonia Spensiero, Simone Di Micco, Mariangela Agamennone, Alessia Bertamino, Ettore Novellino, Giuseppe Bifulco, Isabel M. Gomez-Monterrey, Fabiana Superti, Pietro Campiglia

**Affiliations:** 1 Department of Pharmacy, University of Salerno, Via Giovanni Paolo II 132, 84084 Fisciano, Italy; 2 National Centre for Innovative Technologies in Public Health, National Institute of Health, Viale Regina Elena 299, 00161 Rome, Italy; 3 Department of Pharmacy, University of Chieti “G. d’Annunzio”, Via dei Vestini 31, 66100 Chieti, Italy; 4 Department of Pharmacy, University of Naples “Federico II”, Via D. Montesano 49, 80131 Napoli, Italy

## Abstract

Bovine lactoferrin is a biglobular multifunctional iron binding glycoprotein that plays an important role in innate immunity against infections. We have previously demonstrated that selected peptides from bovine lactoferrin C-lobe are able to prevent both Influenza virus hemagglutination and cell infection. To deeper investigate the ability of lactoferrin derived peptides to inhibit Influenza virus infection, in this study we identified new bovine lactoferrin C-lobe derived sequences and corresponding synthetic peptides were synthesized and assayed to check their ability to prevent viral hemagglutination and infection. We identified three tetrapeptides endowed with broad anti-Influenza activity and able to inhibit viral infection in a concentration range femto- to picomolar. Our data indicate that these peptides may constitute a non-toxic tool for potential applications as anti-Influenza therapeutics.

## Introduction

Bovine lactoferrin (bLf) is a glycoprotein consisting of a single polypeptide chain of 689 amino acid residues, with a molecular mass of 76 kDa, which binds two iron atoms with very high affinity^1^. BLf, like lactoferrin of other mammalian species, is folded in two symmetric and globular lobes: N-lobe (residues 1–333) and C-lobe (residues 345–676) which are further divided in two subdomains (I and II) each with the iron binding at the interdomain cleft. These two lobes are linked by a three-turn connecting helix, residues 334 and 344, which provide additional flexibility to the molecule^2^.

Lactoferrin (Lf) is present in various biological fluids and in specific granules of polymorphonuclear leukocytes^3^, and possesses a variety of biological functions, such as promotion of iron absorption, immunomodulation and inhibiting activity towards different pathogens^4–7^. In particular, bLf has been recognized as a potent inhibitor of different enveloped viruses, such as Human Cytomegalovirus^8, 9^, Herpes Simplex Viruses types 1 and 2^10–13^, Human Immunodeficiency Virus^8^, Human Hepatitis C Virus^14^, Hantavirus^15^, Hepatitis B virus^16^, respiratory syncytial virus^17^, Flavivirus^18^, Alphavirus^19^ and Phlebovirus^20^.

**Figure 1:**
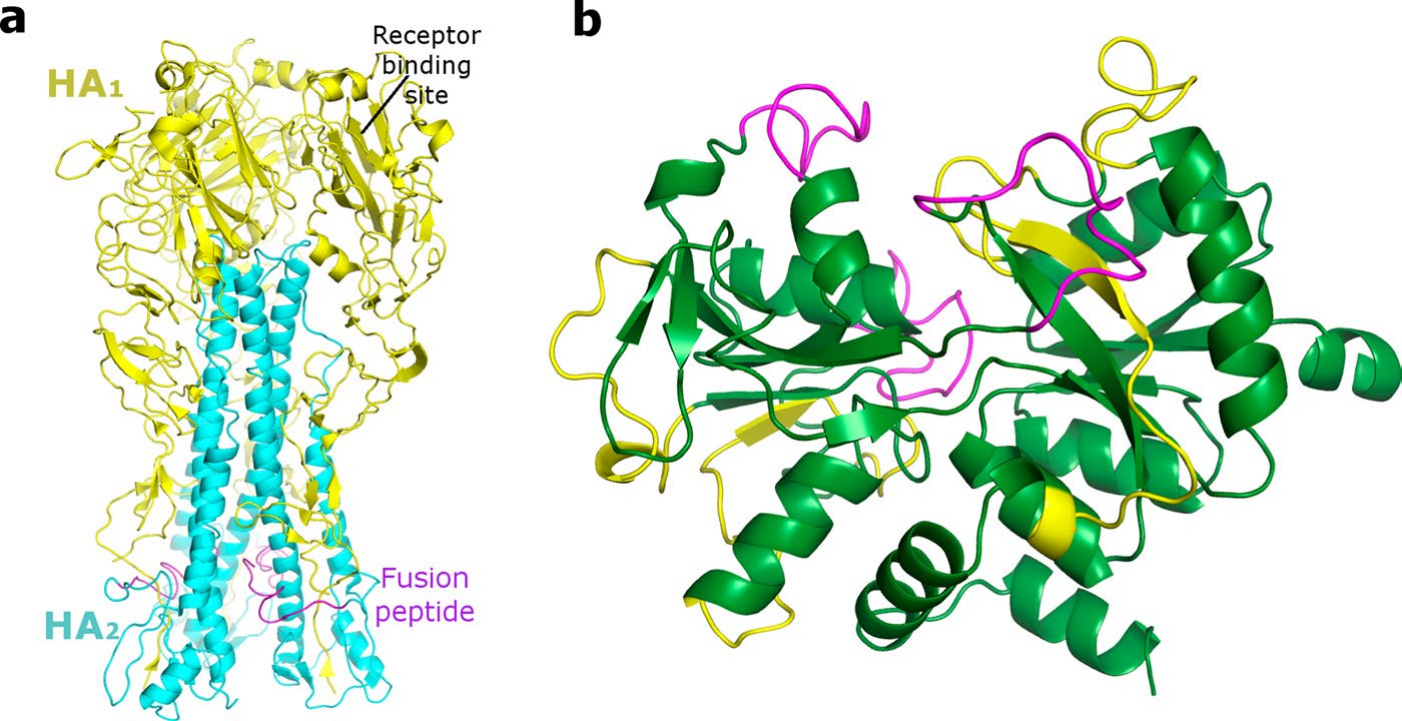
(**a**) Cartoon representation of the HA trimeric protein. The HA_1_ chain has been depicted in yellow and the HA_2_ in cyan. The position of receptor binding site and the fusion peptide have been highlighted. (**b**) Cartoon representation of the bLf C-lobe. The patented sequences are depicted in purple and the newly synthesized sequences in yellow.

In this context, Pietrantoni *et al*. demonstrated that bovine lactoferrin is also able to inhibit the Influenza virus infection by interfering with caspase 3 functions and by inhibiting the export of viral ribonucleoproteins from the nucleus to the cytoplasm^21^. The degree of ion-binding, sialylation or glycosylation of bLf does not affect the inhibition of Influenza virus replication as lactoferrin maintains its antiviral activity in desialylated, deglycosylated, apo and ion-saturated forms^22^. At molecular level, bLf binds Influenza A virus hemagglutinin (HA) inhibiting the hemagglutination and infection of major virus subtypes such as H1N1 and H3N2^23^. Hemagglutinin is the major glycoprotein component of the viral envelope along with neuraminidase (NA); it is a mushroom-shaped trimeric protein and each monomer is composed by two subunits: the HA_1_ and the HA_2_ (Fig. 1a). The HA globular head, constituted by the HA_1_ chain, contains the sialic acid binding sites, while the conserved stem region triggers the conformational rearrangement at acidic pH necessary to produce the infection^24–26^. BLf, and more concretely its C-lobe fragment, has been demonstrated to bind the HA stem region^23^ formed by the HA_2_ domain together with several important residues in the N- and C-terminal segments of the HA_1_ and containing the universally conserved HA epitope^27^. This behavior explains the broad spectrum of the bLf C-lobe anti-Influenza activity^23^.

The alignment of bLf C- and N-lobe sequences and protein–protein docking calculations suggested that the binding between bLf C-lobe and HA is mediated by surface-exposed loops of lactoferrin^23^. In particular, we demonstrated that three C-lobe fragments, 418–429 (SKHSSLDCVLRP, **1**), 506–522 (AGDDQGLDKCVPNSKEK, **2**) and modified sequence 552–563 (NGESSADWAKN, **3**), strongly inhibited viral hemagglutination and infection at low picomolar concentrations^23^, because of this interesting activity, peptides **1**–**3** were patented^28^. These results paved the way to the modulation of the interaction between bLf and HA as a therapeutic anti-Influenza approach.

To better analyze the molecular and structural requirements that determine the bLf C-lobe-HA interaction, we decided to perform a wider mapping of C-lobe domain by designing, synthesizing and evaluating a new library of bLf C-lobe derivatives. Furthermore, we focused on the identification of a minimal active amino acid sequence within one of the most potent identified peptide, the peptide **1** (SKHSSLDCVLRP). For the most effective compounds, the ability of direct binding to viral hemagglutinin was evaluated and their conformation in solution was also determined. The results of these experiments will assist in engineering peptidomimetics or small molecules from bLf C-lobe with a better anti-Influenza activity and therapeutic index.

## Results

### Design

In a previous work we assessed the ability of bLf C-lobe-derived peptides to impair the HA activity. Protein-protein docking simulations, carried out to depict the putative binding mode of involved macromolecules, suggested the main role of three bLf C-lobe loops in the interaction with HA. Synthesized peptides with the corresponding sequences (peptides **1**, **2** and **3**) have demonstrated to be very potent in the prevention of both viral hemagglutination and infection^23^. However, we could not exclude that other bLf portions interact with HA and an in-depth inspection of docked poses (Figure S1) allowed us to select other loops on the bLf surface, as potential HA binders (Fig. 1b, in yellow). The identified sequences are: 441–454 (KANEGLTWNSLKDK, **4**), 478–500 (TGSCAFDEFFSQSCAPGADPKSR, **5**), 552–563 (TNGESTADWAKN, **6**), 619–630 (GKNGKNCPDKFC, **7**), 633–638 (KSETKN, **8**) and 642–659 (NDNTECLAKLGGRPTYEE, **9**).

### Antiviral activity

#### Inhibition of hemagglutination

Peptides **4–9** were synthesized and their ability to inhibit the HA activity was assessed by hemagglutination inhibition assay (HI). The activity of peptide **1** is reported as a reference. The Influenza A virus strains A/Roma-ISS/02/08 H1N1 oseltamivir-sensitive virus, A/Parma/24/09 H1N1 oseltamivir-resistant virus, and A/Parma/05/06 H3N2 were used. As shown in Table 1, three out of six peptides were able to prevent HA activity of all tested viral strains.

**Table 1:** Sequence and HI titer of peptides **1**, **4**–**9**.

Frag	Peptide	Sequence	HI titer (nM)		
			A/Roma- ISS/02/08 H1N1	A/Parma/24/09 H1N1	A/Parma/05/06 H3N2
418–429	**1**	SKHSSLDCVLRP	0.0014^a^	0.0014^a^	0.0007^a^
441–454	**4**	KANEGLTWNSLKDK	12	3	12
478–500	**5**	TGSCAFDEFFSQSCAPGADPKSR	—	—	97
552–563	**6**	TNGESTADWAKN	0.7	1.5	0.3
619–630	**7**	GKNGKNCPDKFC	—	—	—
633–638	**8**	KSETKN	1.5	0.3	3
642–659	**9**	NDNTECLAKLGGRPTYEE	—	2.500	—

^a^Ammendolia *et al*. ref. 23.

Notwithstanding these peptides exerted a strong antiviral action in the nanomolar range, their activity was lower than that previously described for peptides **1**–**3** (low picomolar)^23^. For example, the most potent compound of this series, dodecapeptide **6**, having a sequence similar to undecapeptide **3**, proves to be several orders of magnitude less potent than this latter^23^.

According to these results, peptide **1** demonstrated to be a potent antiviral peptide, inhibiting influenza virus hemagglutination at picomolar concentration. Therefore, we focused on this peptide to carry out an optimization study. To this aim we designed a new small library of peptides through addition of four amino acid residues at both the N- and C-terminals (peptide **10**), at the N-terminal (compound **11**) and at the C-terminal (compound **12**) of the ^418^SKHSSLDCVLRP^429^ sequence (**1**).

We also designed a truncation library to identify the shortest amino acid sequence needed for the peptide activity. The truncation process was carried out via a systematic reduction of four residues at both the N- and C-terminals of the peptide **1** (compounds **13**–**17**).

According to the results showed in Table 2, N-terminal truncation strategy led to the most interesting results. Thus, octapeptide **13** was the most effective fragment of this series in preventing HA activity of all tested viruses in a concentration range 0.15 to 0.7 pM.

**Table 2:** Sequence and HI titers of peptides **1**, **10**–**17**. The HI activity of peptide **1** is reported as a reference.

Frag.	Peptide	Sequence	HI titer (nM)		
			A/Roma- ISS/02/08 H1N1	A/Parma/24/09 H1N1	A/Parma/05/06 H3N2
418–429	**1**	SKHSSLDCVLRP	0.0014^a^	0.0014^a^	0.0007^a^
414–433	**10**	NRKSSKHSSLDCVLRPTEGY	0.3	6	0.0014
414–429	**11**	NRKSSKHSSLDCVLRP	—	12	0.4
418–433	**12**	SKHSSLDCVLRPTEGY	0.7	0.1	0.03
422–429	**13**	SLDCVLRP	0.0007	0.00015	0.0004
426–429	**14**	VLRP^**b**^	0.7	0.15	1.5
422–425	**15**	SLDC^**b**^	0.0014	6	1.5
418–425	**16**	SKHSSLDC	0.3	12	12
418–421	**17**	SKHS^**b**^	0.1	1.5	12

^a^Ammendolia *et al*. ref. 23.

^b^The peptides 14, 15, 17 are acetylated and amidated at N-terminal and C-terminal, respectively.

The C-terminal tetrapeptide of **1**, peptide **14**, lost activity toward all strains used in the assay, indicating the relevance of N-terminal residue of **1** and **13** for the hemagglutination inhibition activity, while the tetrapeptide **15** maintained the same inhibition level of **1** on Influenza A/Roma-ISS/02/08 H1N1 strain, showing a remarkable inhibitory selectivity toward this virus (1000 fold) compared to the A/Parma H1N1 and H3N2 strains. This same activity profile was also observed with compounds **16** and **17**, derived from the C-terminal deletion on the lead sequence 418–429, that were more potent against A/Roma strain than compared to the other viral strains.

#### Neutralization assay

Thereafter, we assessed the ability to affect virus replication in Madin-Darby canine kidney (MDCK) cell line of most potent peptides of the first series (**4**, **6**, **8**) and fragments **13**–**17** by neutralization assay. As shown in Table 3, peptides **4**, **6** and **8** were able to prevent infection of all tested viruses in a concentration range from about 0.5 pM to 400 nM, with a relevant reduction of activity and, consequently, of the selectivity index (SI ≈ 10^2^/10^7^).

**Table 3:** *In vitro* antiviral activity of most potent peptides against influenza virus infection.

Pep.	Sequence	A/Roma-ISS/02/08 H1N1		A/Parma/24/09 H1N1		A/Parma0/5/06 H3N2	
		EC_50_^a^ (pM)	SI^	EC_50_^a^ (pM)	SI^	EC_50_^a^ (pM)	SI^
**1**	SKHSSLDCVLRP	4 ± 0.37^b^	>6.25.10^6^	3.1 ± 0.12^b^	>8.10^6^	5.8 ± 0.7^b^	>4.4.10^6^
**4**	KANEGLTWNSLKDK	1 ± 0.15	>2.5.10^7^	50.000 ± 250	>5.10^2^	1.000 ± 360	>2.5.10^4^
**6**	TNGESTADWAKN	400 ± 0.02	>6.25.10^4^	50.000 ± 230	>5.10^2^	10.000 ± 120	>2.5.10^3^
**8**	KSETKN	0.5 ± 0.01	>5.10^7^	500 ± 0.46	>5.10^4^	400.000 ± 210	>0.65.10^2^
**13**	SLDCVLRP	0.3 ± 0.5	>8.33.10^7^	2.5 ± 0.37	>1.10^7^	300 ± 0.2	>8.33.10^4^
**14**	VLRP	0.45 ± 0.1	>5.55.10^7^	1 ± 0.05	>2.5.10^7^	250 ± 0.42	>1.10^5^
**15**	SLDC	0.5 ± 0.001	>5.10^7^	4.6 ± 0.05	>5.4.10^6^	4.3 ± 0.03	>5.8.10^7^
**16**	SKHSSLDC	80 ± 0.19	>3.125.10^5^	0.1 ± 0.001	>2.5.10^8^	5.0 ± 0.45	>5.10^6^
**17**	SKHS	3 ± 0.61	>8.33.10^6^	0.048 ± 0.0012	>5.2.10^8^	5.0 ± 0.02	>5.10^6^

^a^EC_50:_ the reciprocal substance dilution at which 50% of cells were protected from the virus induced killing; ^SI: the ratio between CC_50_ (the reciprocal substance dilution at which 50% of cells were protected from substance toxicity, corresponding to a concentration >25 μM) and EC_50_; The mean values of 3 independent experiments with standard errors are shown.

^b^Ammendolia *et al*. ref. 23.

The octapeptide **13** (SLDCVLRP), the most active in the prevention of viral hemagglutination (HI titer 0.15–0.7 pM, Table 2), conserved a good antiviral activity against the two A H1N1 strains with EC_50_ values of 0.3 and 2.5 pM, respectively, but lost activity against H3N2 strain with respect to **1** (≈50 fold, EC_50_ = 300 pM). Tetrapeptide **14**, containing a net positive charge (Arg^428^), showed a similar activity profile to peptide **13**, being more active against both H1N1 strains compared to reference peptide **1**. Tetrapeptide **15**, with an opposite net charge with respect to **14** (Asp^424^), was strongly effective against A/Parma/05/06 H3N2 strain (EC_50_ = 4.3 pM). Derivatives obtained by the C-terminal deletion of **1** (peptides **16** and **17**) showed a different behaviour: octapeptide **16** was 30 and 10 fold more potent than **1** and **13**, respectively, to prevent viral infection against A/Parma/24/09 H1N1 strain, while it was 20 and 270 fold less active than these, against A/Roma- ISS/2/08 H1N1 strain. The N-terminal tetrapeptide **17** containing two positive residues (Lys^419^-Hys^420^) regains antiviral activity on this latter strain, maintaining high antiviral activity at femto- and picomolar concentration against both A/Parma H1N1 and H3N2 strains, respectively. These results suggest that tetrapeptides **14**, **15** and, in particular, **17** could be good starting point in the search for new peptidomimetics and small molecules candidates for Influenza virus treatment as well as in the search for new peptide formulation with the same aim.

### SPR-based binding assays

**Figure 2:**
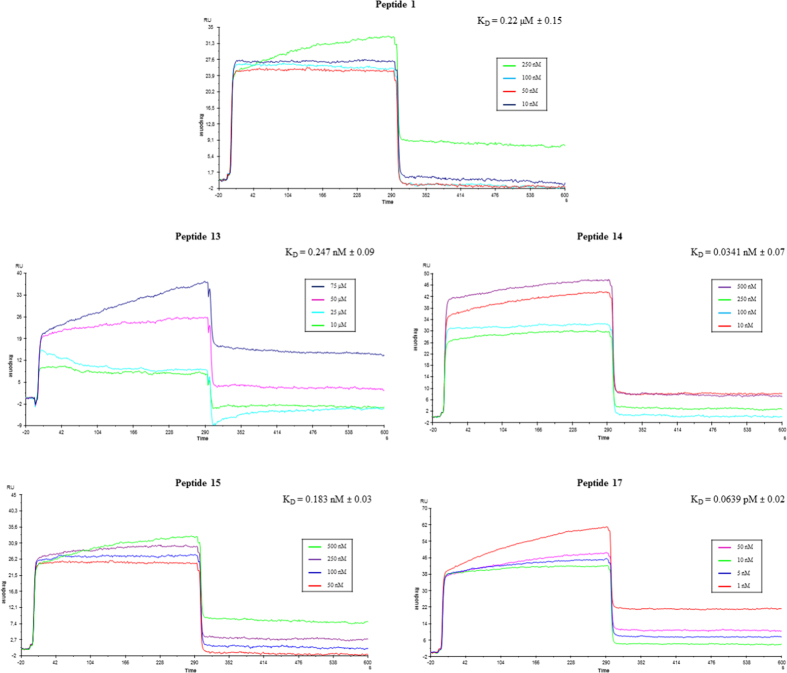
Sensorgrams obtained from SPR interaction analysis of compounds **1**, **13**, **14**, **15** and **17** binding to immobilized HA. Each compound was injected at four different concentrations (from 1 nM to 75 µM).

Peptide **1**, **13** and tetrapeptides **14**, **15** and **17** were tested by surface plasmon resonance (SPR) assays^29^ to evaluate the capability of compounds to bind the viral hemagglutinin. The HA protein was immobilized (up to ~ 27 000 response units, RU) on different flow cells of the biosensor chip and tested ligands were injected at various concentrations (from 1 nM to 100 µM) over the protein surface. A regeneration step was necessary (1 mM NaOH, data not shown). After injection, running buffer was allowed to flow over the surface and the dissociation of compounds from the surface was observed (Fig. 2). In contrast, the control flow cell, where no HA was immobilized, showed no significant signal changes (data not shown). In addition, to evaluate a potential no specific binding, all compounds were injected on immobilized GRK2 protein. Equilibrium dissociation constant (Kd) values were derived from the ratio between kinetic dissociation (kd) and association (ka) constants, obtained by fitting data from all injections at different concentrations of each compound using the simple 1:1 Langmuir binding fit model of the BIA evaluation software.

The bLf analogues efficiently interacted with the immobilized protein. Figure 2 shows the sensorgrams of compounds **1**, **13**, **14**, **15** and **17** bound to HA in HBS-N buffer. Interestingly, the three tetrapeptides bind HA with higher efficiency with respect to **1**, the most effective being the peptide **17** that showed a Kd value of 0.0639 pM. The order of peptide binding affinity was **17** > **14** > **15** > **13** > **1**. In order to evaluate the specificity of the inhibitory peptides, peptide **4** was used as a control (Figure S16).

### NMR analysis of peptide 1, 4, 13–15 and 17

**Figure 3:**
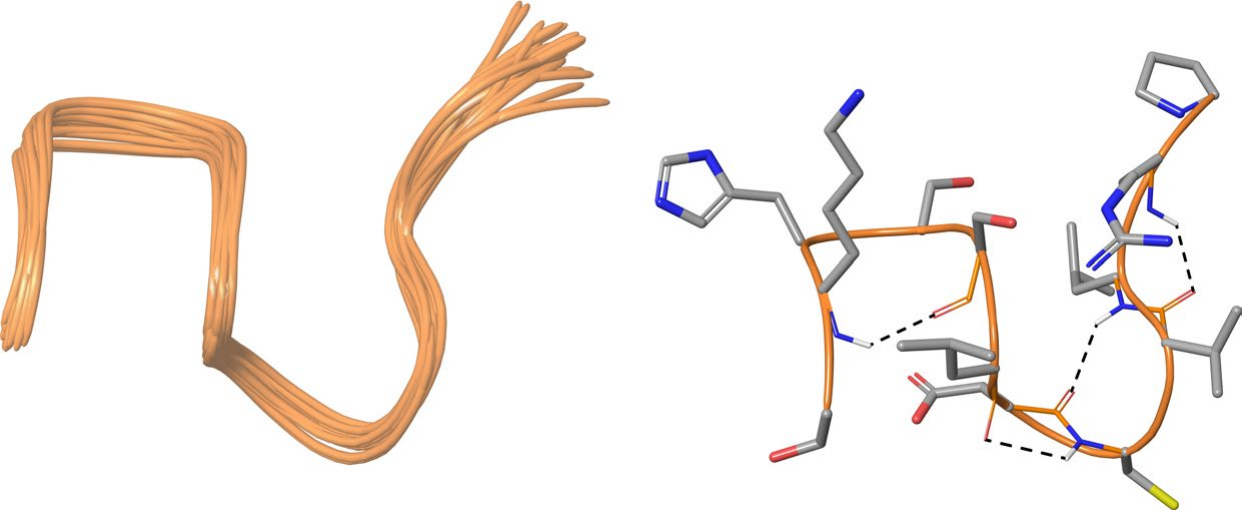
On the left, superposition of backbone atoms of twenty NMR structures of **1** (orange ribbons) generated by using CYANA 2.1. On the right, the average NMR derived structures of **1**. The atoms are depicted in tube and colored by atom types (O, red; N, blue; S, yellow; polar hydrogen, white). The backbone C atoms of **1** are colored as for the ribbons and the side chain C atoms are in grey. The dashed lines indicate intramolecular H-bonds responsible of the global fold.

The solution-state structure (HFA/H_2_O) of the oligopeptide SKHSSLDCVLRP (**1**) was obtained by 2D NMR spectroscopy. In details, according to standard procedures^30^, the chemical shift assignments of the ^1^H resonances (Table S2) have been achieved by using DQF-COSY^31^, TOCSY^32^ and NOESY^33^ experiments. A set of 117 inter-proton distance restraints were collected from 2D-NOESY NMR experiments (*t*_mix_ = 400 ms) and used in simulated annealing protocol of the software CYANA 2.1^34^. The NMR structure bundle (Fig. 3, left) of SKHSSLDCVLRP shows high structural agreement with RMSD of 0.29 Å referenced to the backbone atoms. By means of PROMOTIF software^35^, the quantitative analysis of φ and ψ dihedral angles of the representative structures of SKHSSLDCVLRP was carried out, highlighting a global turn conformation. In particular, **1** contains four β-turns (type IV) formed by residues: 2–5, 4–7, 6–9 and 7–10 (Table S2). It was also observed a γ-turn structure involving the residues Val9-Arg11 (Table S2).

**Figure 4:**
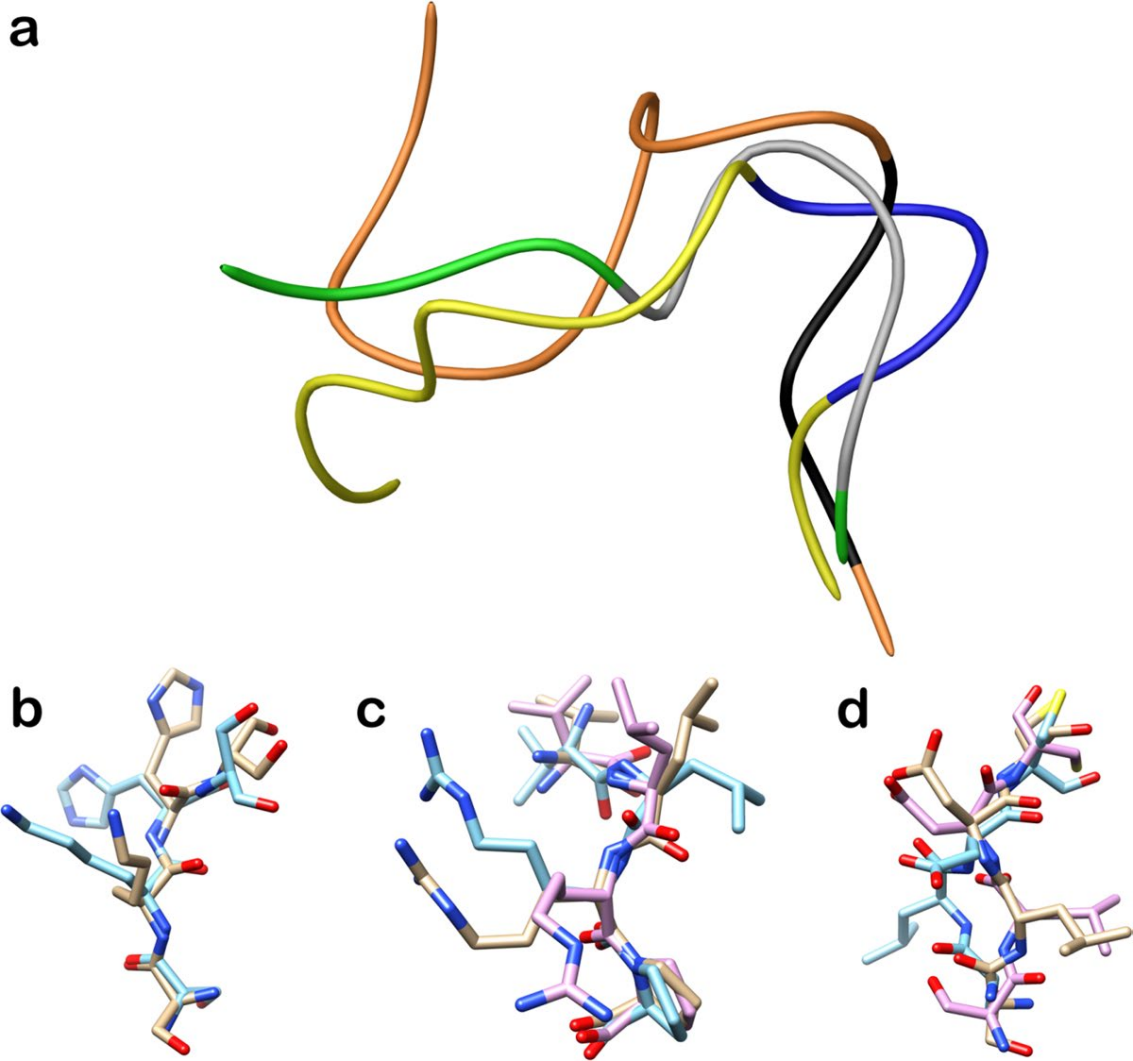
(**a**) Superimposition of loop Ser418-Pro429 (yellow and blue ribbon) of lactoferrin (PDB ID: 3IB0), **1** (orange and black ribbon) and **13** (green and grey ribbon). The blue, black and grey portions indicate the helix 3_10_ of Ser418-Pro429, the γ-turn of **1**, and β-turn of **13**, respectively. (**b**) Superimposition of first four amino acids (SKHS) of **1** (cyan) and loop Ser418-Pro429 (tan). (**c**) Superimposition of last four amino acids (VLRP) of **1** (cyan), **13** (purple) and loop Ser418-Pro429 (tan). (**d**) Superimposition of SLDC amino acids of **1** (cyan), **13** (purple) and loop Ser418-Pro429 (tan). The atoms are depicted in tube and colored by atom types (O, red; N, blue). The C atoms of **1**, **13** and loop Ser418-Pro429 are colored as for tube.

The overall turn conformation observed for **1** is in line with spatial arrangement of the loop Ser418-Pro429 of C-terminal lobe of lactoferrin (PDB ID: 3IB0). In particular, we observed that the helix 3_10_ formed by Cys425-Leu427 of protein loop is overlapped with the γ-turn of **1** centered on Val9-Arg11 (Fig. 4a). Moreover, we observed a very good superimposition between **1** and the loop Ser418-Pro429 in the first four amino acids (SKHS, Fig. 4b), suggesting this conformation as a structural requirement for the resulting peptide activity as shown by **17**.

**Figure 5:**
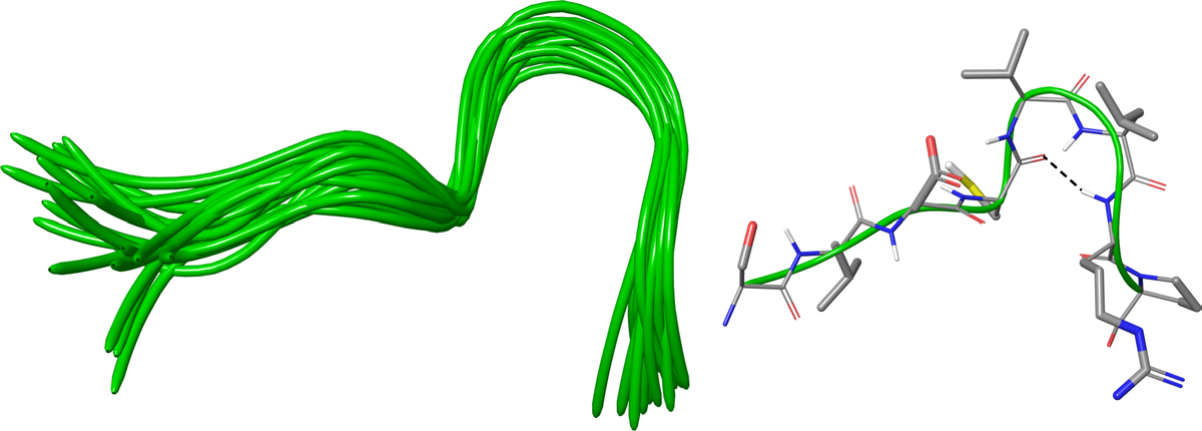
On the left, superposition of backbone atoms of twenty NMR structures of **13** (green ribbons) generated by using CYANA 2.1. On the right, the average NMR derived structures of **13**. The atoms are depicted in tube and colored by atom types (O, red; N, blue; S, yellow; polar hydrogen, white). The backbone C atoms of **13** are colored as for the ribbons and the side chain C atoms are in grey. The dashed lines indicate intramolecular H-bonds responsible of the global fold.

We also investigated the solution structure of octapeptide **13** (SLDCVLRP), by following the same strategy adopted for **1**. The NMR conformation bundle (Fig. 5, left) of **13** presents high structural definition with an RMSD of 0.70 Å referenced to the backbone atoms. As observed for **1**, the quantitative analysis of φ and ψ dihedral angles of the representative structures of **13** revealed a global turn arrangement. In particular, the octapeptide **13** (SLDCVLRP) contains a β-turn (type IV) involving the residues Cys4-Arg7 (Table S3). Such β-turn conformation of **13** is overlapping with the helix 3_10_ formed by Cys425-Leu427 of lactoferrin (PDB ID: 3IB0) and the γ-turn of **1** delimited by Val9-Arg11 (Fig. 4a). We superimposed the residues VLRP and SLDC of **1**, **13** and the loop Ser418-Pro429 (Fig. 4c,d). For the common VLRP portion of **1**, **13** and the loop Ser418-Pro429 (Fig. 4c) a very similar spatial arrangement was observed. For what concerns residues SLDC of **1**, **13** and the loop Ser418-Pro429 (Fig. 4d), a very good conformational similarity was found between protein loop and **13**, whereas **1** slightly diverges in the leucine spatial arrangement. These structural findings could suggest the structural elements responsible for the resulting peptide activities of **14** and **15**.

**Figure 6:**
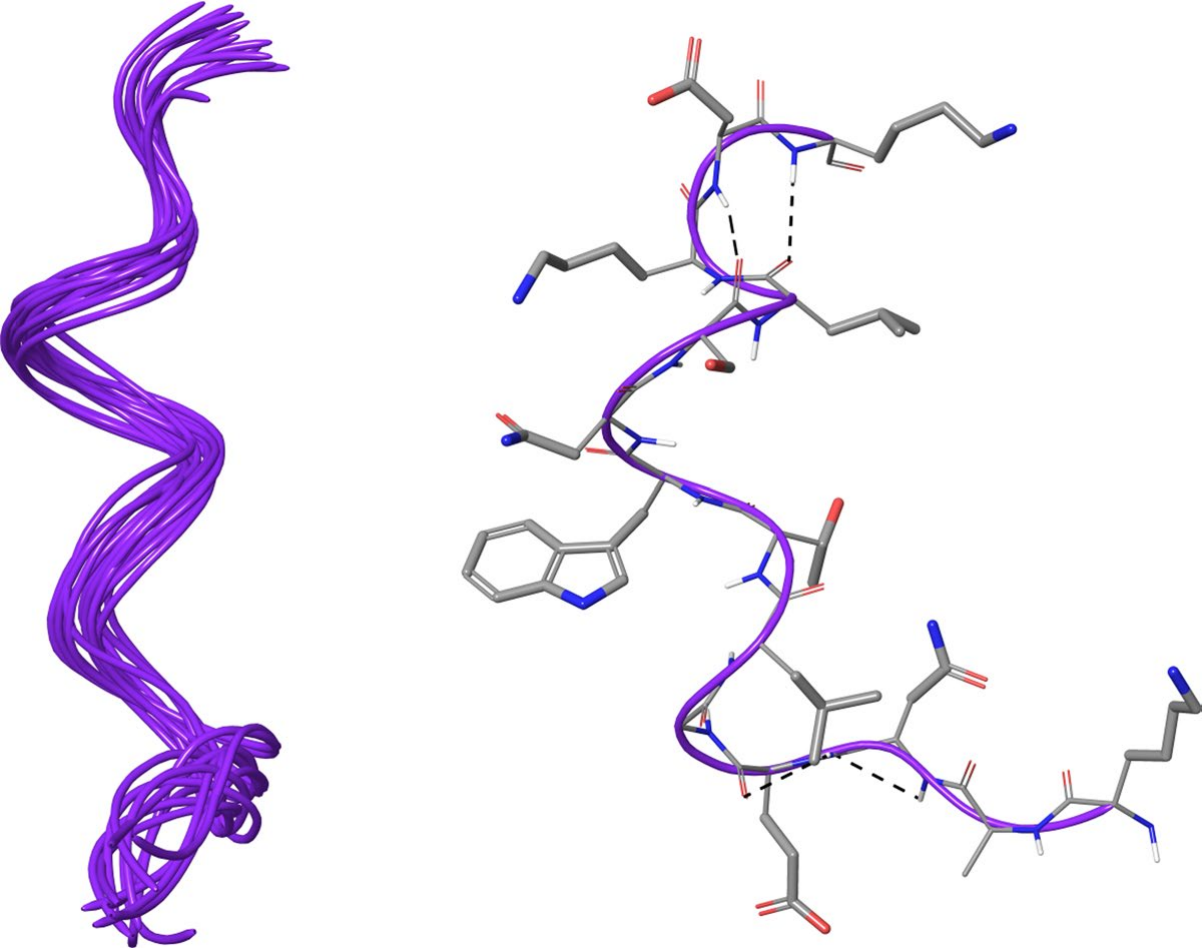
On the left, superposition of backbone atoms of twenty NMR structures of **4** (purple ribbons) generated by using CYANA 2.1. On the right, the average NMR derived structures of **4**. The atoms are depicted in tube and colored by atom types (O, red; N, blue; S, yellow; polar hydrogen, white). The backbone C atoms of **4** are colored as for the ribbons and the side chain C atoms are in grey. The dashed lines indicate intramolecular H-bonds responsible of the global fold.

In order to confirm the turn conformational arrangement as fundamental for the biological activity, we determined the solution structure of peptide **4** (KANEGLTWNSLKDK) that weakly inhibits the infection. In the same experimental conditions for **1** and **13**, the NMR conformation bundle (Fig. 5, left) of **4** shows a high structural convergence with an RMSD of 1.01 Å referenced to the backbone atoms (Fig. 6). It is noteworthy that, differently from **1** and **13**, we observed a peculiar helix G formed by residues Leu11-Asp13, corroborating the structural outcomes suggested by the analysis of the active peptides.

Similarly to **1**, **4** and **13**, we tried to assign the ^1^H resonances of tetrapeptides **14** (VLRP), **15** (SLDC) and **17** (SKHS) in HFA/H_2_O, but most of resonances resulted overlapped. Thus, we assigned the ^1^H resonances in DMSO (Tables S4–S6)^36, 37^ of tetrapeptides **14** (VLRP), **15** (SLDC) and **17** (SKHS). We also attempted to determine the solution structures of **14**, **15** and **17** by collecting interproton distance restraints from 2D-NOESY and 2D-ROESY^38^ experiments at different mixing time, but the very low number of inter-residue NOE effects hampered this task. This was due to the expected high flexibility of the tetrapeptides in solution, nevertheless we may assume that the preferred conformation of **14**, **15** and **17** is similar to the spatial arrangement observed for **1**, **13** and the loop Ser418-Pro429 as highlighted by the biological activity of **14**, **15** and **17**.

## Discussion

The effective treatment of Influenza virus infection is still an unmet goal. Several compounds are in clinical phases for this pathology^39^ and most of them target the HA, one of the surface exposed glycoprotein of Influenza virus. HA, in fact, is a promising target because it plays a relevant role in both cell surface attachment and virion internalization. Peptides impairing the viral entry in the cell are actively searched also because of the well-established strategy setup for Enfuvirtide, an HIV inhibitor peptide approved by FDA^40^. Therefore, most peptides have been identified that block the Influenza virus by several mechanisms^41^. In this context, we demonstrated the anti-Influenza activity of bLf derived peptides **1**–**3**, mediated by the interaction with HA^23^. In the present work, we went in-depth on the demonstrated interaction between bovine lactoferrin C-lobe and HA attempting to enlarge the number of bLf derived peptides active toward Influenza virus. The synthesis and biological assays on peptides **4**–**9** confirmed lactoferrin to be an effective source of active peptides, even though not all tested peptides showed activity nor improved the good activity profile observed for peptides **1**–**3**. The lack of antiviral effect by some of the tested peptides suggests that the antiviral property of these compounds is specific to those peptides and neither a general property of any oligomeric peptide nor based on charge or hydrophobic interactions.

The almost complete exploration of the bLf C-lobe loops allowed to confirm the interest on peptides **1**–**3** that is counterbalanced by some issues due to the pharmaceutical development of peptides constituted by 11–17 amino acids. It is well known that clinical administration of peptides raises several issues because of the instability of the amide bond, therefore, our final objective is the development of peptidomimetics that, mimicking our active peptides, maintain their potency and broad spectrum activity and can be clinically exploited for the treatment of Influenza virus infection. To reach this goal a necessary step is represented by the identification of the minimum peptide bringing the antiviral activity. In the present work the starting point is represented by one of most potent bLf derived peptides (**1**). Surprisingly, all the three obtained tetrapeptides **14**, **15** and **17** conserved the anti-Influenza effect toward both H1N1 and H3N2 strains, that is meaningful for a broad spectrum activity toward HAs belonging to different clades. Peptide **17**, in particular, was more active than **1** in neutralization assays, confirming the interest toward this peptide for further developments. Moreover, the SPR-based binding assays confirm that the antiviral activity is due to the interaction with HA as demonstrated also by HI assays. The HI, SPR and antiviral activity assays look at the interaction with the target protein (HA) from different point of view and this fact could explain the divergence of the obtained results. The activity showed in the HI and neutralization assays relies on the direct binding of our peptides with the receptor binding site on the HA surface (HA_1_ subunit, Fig. 1a). However, as hemagglutinin is a very large protein, it cannot be excluded that our peptides can bind also some other region of HA even though we have no direct demonstration of it. This behavior has been observed also for the bLf C-lobe (that blocks the hemagglutination and binds HA_2_) and for small molecules previously developed by us^42^. An alternative/adjunctive binding on the HA surface could affect both SPR assay results and antiviral activity. Moreover, the interaction of our peptides with hemagglutinin results in the modification of HA functions, and thus influenza virion cannot bind to the host cell membrane. This alteration of HA functions could also influence viral fusion by blocking HA conformation change that commonly leads to capsid escape from endosome and dissemination of viral genome.

The different results of HI and *in vitro* antiviral activity assays for the two H1N1 strains (one oseltamivir-sensitive virus and one oseltamivir-resistant virus) is likely due to the different exposition of HA in virus with different NA. In fact, the oseltamivir-resistant virus possesses a mutated NA that could result in a different response to peptides in *in vitro* antiviral assay in which both viral attachment and fusion can be possible targets. Moreover, the two H1N1 strains have very similar HAs that differ in four residues surrounding the receptor binding site. This slight difference could affect the HI results, that depends on the binding of ligands in this region, and the antiviral activity that is certainly influenced by this interaction.

NMR spectroscopy analysis performed on compounds **1** showed a global turn conformation for this peptide and hypothesized the preferred bioactive conformation of our tetrapeptides.

Further steps will be aimed at improving the stability and pharmaceutical suitability of identified peptides with the synthesis of modified peptides/peptidomimetics along with the activity profile definition enlarging the panel of tested viral strains.

In conclusion, this study describes the identification of three C-lobe bLf-derived tetrapeptides as the minimum fragments expressing the broad anti-influenza activity of bLf. Peptides **14** (VLRP), **15** (SLDC) and **17** (SKHS) were designed from the fragment 418–429 (SKHSSLDCVLRP, **1**), which is involved in the C-lobe bLf-HA interaction. These tetrapeptides retain the inhibitory potency of the fragment 418–429 and inhibit the Influenza virus hemagglutination and cell infection in a concentration range of femto- to picomolar. SPR assay confirmed a high affinity of **1**, **13**, **14**, **15** and **17** for HA protein, suggesting also that this protein could be considered the potential molecular target of our peptides. In the context of the search for anti-Influenza peptides, our findings stand out because of both the potency of identified peptides and the small dimensions of compounds **14**, **15** and **17**; in fact, at the best of our knowledge, they are the smallest peptides endowed with anti-Influenza activity.

Our results strongly encourage the pursuit of this path for the development of a novel class of anti-Influenza drugs.

## Methods

### Material and chemicals

N^α^-Fmoc-protected amino acids, Wang resin, Rink amide-resin, coupling reagents, N,N-Diisopropylethylamine (DIEA), piperidine and trifluoroacetic acid (TFA) were purchased from Iris Biotech (Germany). Rink Amide-ChemMatrix resin was purchased from Biotage AB (Sweden). Peptide synthesis solvents, reagents, as well as CH_3_CN for High Performance Liquid Chromatography (HPLC) were reagent grade and were acquired from commercial sources and used without further purification unless otherwise noted.

#### Cells and Viral strains

Madin-Darby canine kidney (MDCK, ATCC, CRL-2936) cells were grown at 37 °C in minimal essential medium (MEM, Invitrogen, Paisley, UK) containing 1.2 g/l NaHCO_3_, and supplemented with 10% inactivated fetal calf serum (FCS, Invitrogen, Paisley, UK), 2 mM glutamine, nonessential amino acids, penicillin (100 IU/ml), and streptomycin (100 μg/ml).

The following influenza A virus strains were used: A/RomaISS/02/08 H1N1 (Brisbane-like) oseltamivir-sensitive virus, A/Parma/24/09 H1N1 (Brisbane-like) oseltamivir-resistant virus, and A/Parma/05/06 H3N2 (Wisconsin-like). Virus titers were determined by a hemagglutinin titration and/or plaque assay according to the standard procedures^43, 44^.

### Peptide synthesis

The synthesis of bLf analogues (**8**, **13–17**) was performed according to the solid phase approach using standard Fmoc (9-Fluorenylmethoxycarbonyl) methodology^45, 46^ in a manual reaction vessel on a Rink amide resin (0.150 g, loading 0.59 mmol/g) previously Fmoc-deprotected by a 25% piperidine solution in DMF (1 × 5 min and 1 × 25 min). Each coupling reaction was accomplished using a 3-fold excess of amino acid with HBTU (2-(1H-benzotriazole-1-yl)-1,1,3,3-tetramethyluronium hexafluorophosphate) and HOBt (1-Hydroxybenzotriazole) in the presence of DIEA (6 eq.). The peptide resin was washed with dichloromethane (DCM, 3×), N,N-dimethylformamide (DMF, 3×), and DCM (3×) and the Fmoc deprotection protocol, described above, was repeated after each coupling step. After peptide assembling the N-terminal Fmoc group was removed and the peptides (**14**, **15** and **17**) were acetylated adding a solution of Ac_2_O/DCM (1:3) shaking for 30 min. Finally, peptides were released from the resin using a cleavage mixture containing 90% TFA, 5% Triisopropylsilane (TIS) and 5% H_2_O for 3 h. The resin was removed by filtration, and the crude peptide was recovered by precipitation with cold anhydrous ethyl ether to give a white powder and then lyophilized.

Peptides **4–7**, **9**, **10**–**12** were synthesized using an Automated Microwave Peptide Synthesizer from Biotage AB (Initiator + Alstra™). Peptides were synthesized on a Wang-ChemMatrix resin (0.150 g, loading 0.3 mmol/g). Peptides were synthesized as previously described^47^.

All crude peptides were purified by RP-HPLC on a preparative C18-bonded silica column (Phenomenex Kinetex AXIA 100 Å, 100 × 21.2 mm, 5 µm) using a Shimadzu SPD 20 A UV/VIS detector, with detection at 214 and 254 nm. The column was perfused at a flow rate of 15 ml/min with solvent A (5%, v/v, water in 0.1% aqueous TFA) and a linear gradient from 5 to 90% of solvent B (85%, v/v, acetonitrile in 0.1% aqueous TFA) over 20 min was adopted for peptide elution. Analytical purity and retention time (t_r_) of each peptide were determined using HPLC conditions in the above solvent system (solvents A and B) programmed at a flow rate of 0.800 ml/min using a linear gradient from 5 to 90% B over 11 min, fitted with C-18 column Supelco, Ascentis express peptide C18 column (50х3.00 mm, 2.7 µm). All analogues showed >97% purity when monitored at 220 nm. Homogeneous fractions, as established using analytical HPLC, were pooled and lyophilized. Ultra high resolution mass spectra were obtained by both positive ESI infusion and MALDI on a Bruker Solarix FT-ICR 7-Tesla. For ESI-MS the instrument was tuned with a standard solution of NaTFA. Spectra were recorded in the range 150–3000 m/z, with an ion accumulation of 0.040 ms, at a transient time of 1 megawards. Source parameters: Dry gas temperature 200 °C, nebulizer (N_2_) and drying gas (Air) 1 and 4 L/min, capillary voltage+4.0 kV. Peptides were directly infused by syringe pump at a concentration of 1 mg/mL in water. MALDI spectra were recorded in the range 150–4000 m/z with the following parameters ion accumulation 0.060 ms, transient time 2 megawards, laser shots 20, laser power 18%, frequency 1000 Hz. For MALDI analyses peptides were spotted on an anchor chip stainless steel plate using alpha cyano 4 hydroxycinnamic acid as matrix.

### Cytotoxicity assay

This procedure was performed as reported elsewhere^21^. Briefly, two-fold serial dilutions of each protein in culture medium were incubated at 37 °C with confluent MDCK cells grown in 96-well tissue culture microplates (Nalge Nunc Europe Ltd, Neerijse, Belgium). After 24 hours, cell morphology, viability and proliferation were evaluated. Protein dilutions that did not affect any of these parameters were considered as non-cytotoxic concentrations and utilized for antiviral assays.

### Hemagglutination inhibition assay

Virus in PBS (Phosphate-buffered saline) was incubated for 1 hour at 4 °C with serial dilutions of bLf or peptidic fragments in PBS. An equal volume of 0.5% turkey erythrocytes was then added and allowed to agglutinate. Titers were expressed as the reciprocal of the protein dilutions giving 50% hemagglutination of erythrocytes by four virus-agglutinating units.

### Neutralization assay

Neutralization was carried out by incubating serial two-fold peptide fragment dilutions, starting from 12.5 µM, in culture medium with equal volumes of virus suspension containing 10^6^ p.f.u. for 1 hour at 4 °C. In negative controls, culture medium was used instead of peptide fragments in the same volume. MDCK cells, grown in 96-well tissue culture microplates (Nalge Nunc Europe Ltd, Neerijse, Belgium), were infected with 100 μl/well (10 p.f.u./cell; in quadruplicate) of the virus-peptide mixtures. After adsorption, cells were rinsed thoroughly and incubated at 37 °C for 24 hours. The viral cytopathic effect (c.p.e.) was measured by neutral red staining as reported elsewhere by our laboratory^11^.

### SPR analysis

The affinity of synthetic peptides for HA was determined by SPR using a Biacore 3000 optical biosensor equipped with research-grade CM5 sensor chips (Biacore AB). Recombinant Full-Length Hemagglutinin (BioVision, catalog no. 4844–10) was immobilized (41,68 μg/mL in 10 mM sodium acetate, pH 4.5) at a flow rate of 10 μL/min by using standard amine-coupling protocols^48^ to obtain densities of 27−28 kRU.

HBS-N buffer (0.01 M HEPES pH 7.4, 0.15 M NaCl) was used as a running buffer. After the immobilization of HA, HBS-N buffer was injected over the chip at a flow rate of 5 μL/min overnight. A solution of peptide in HBS-N buffer at various concentrations (from 1 nM to 100 µM) was injected at 25 °C with a flow rate of 10 μL/min for 5 min (association phase), and then the buffer alone was injected for 5 min (dissociation phase).

Regeneration of the surface was performed, when necessary, by a 10 s injection of 1 mM NaOH.

The simple 1:1 Langmuir binding fit model of the BIAevaluation software (GE Healthcare, version 4.1) was used for determining equilibrium dissociation constants and kinetic dissociation and association constants using Equations (1) and (2), where R represents the response unit, and C is the concentration of the analyte.

1 $$\frac{dR}{dt}=ka\times C\,(Rmax-R)-kd\times R$$

2 $$Kd=kd/ka$$

### NMR experiments and structure calculation

The NMR sample of **1** was obtained dissolving 1 mg of the oligopeptide in 50% of hexafluoroacetone and 50% of H_2_O (10 mM of KH_2_PO_4_) and placed in a 3 mm NMR tube (200 μl). As for **1**, 1.3 mg of **13** and 0.85 mg of **4** were dissolved in 200 μl of 50% of hexafluoroacetone and H_2_O (10 mM of KH_2_PO_4_). The compounds **14** (1.7 mg), **15** (1.3 mg) and **17** (3.4 mg) was dissolved in 500 μl of [D6] DMSO^36, 37, 49^.

All NMR experiments were performed on a Bruker DRX 600 spectrometer equipped with a cryoprobe at *T* = 300 K. All spectra were acquired in the phase-sensitive mode, and the TPPI method was used for quadrature detection in the *ω*_1_ dimension^50^. The residual water signal was suppressed by excitation sculpting with gradients. Data block sizes of 4096 in *t*_2_ and 512 equidistant *t*_1_ values were used. Before Fourier transformation, the time domain data matrices were multiplied by shifted sine bell QSINE (SSB = 2) functions in both dimensions. For **1**, the DQF-COSY, 2D-TOCSY and 2D-NOESY experiments were executed with a number of 48 scans/*t*_1_ and a *t*_1*max*_ value of 81.3 ms. For **4** and **13**, the DQF-COSY, 2D-TOCSY and 2D-NOESY experiments were executed with 16 scans/*t*_1_, 56 scans/*t*_1_ and 56 scans/*t*_1_, respectively, with a *t*_1*max*_ value of 94.8 ms. For **14**, the DQF-COSY, 2D-TOCSY and 2D-ROESY experiments were executed with 16 scans/*t*_1_, 16 scans/*t*_1_ and 48 scans/*t*_1_, respectively, with a *t*_1*max*_ value of 64.0 ms. For **15**, the DQF-COSY, 2D-TOCSY and 2D-ROESY experiments were executed with 16 scans/*t*_1_, 18 scans/*t*_1_ and 48 scans/*t*_1_, respectively, with a *t*_1*max*_ value of 85.3 ms. For **17**, the DQF-COSY, 2D-TOCSY and 2D-ROESY experiments were executed with 16 scans/*t*_1_, 24 scans/*t*_1_ and 64 scans/*t*_1_, respectively, with a *t*_1*max*_ value of 64.0 ms. A mixing time of 80 ms was used for the 2D-TOCSY experiments. 2D-NOESY and 2D-ROESY experiments were run with mixing times in the range of 100−550 ms. SPARKY software was used for qualitative and quantitative analyses of 2D spectra^51^. The obtained peak volumes were converted into upper distance bounds with the CALIBA routine from the CYANA software package. The pseudoatom corrections were applied for non-stereospecifically assigned protons of methylene and methyl groups. The experimentally derived constraints were used to generate an ensemble of 200 structures with the standard CYANA protocol of simulated annealing in the torsion angle space (using 50,000 steps). The best 20 structures that had low target function values and small residual violations were selected. All the 3D models were depicted using the Chimera 1.10.1^52^ and Maestro 9.6 (Schrödinger, *LLC*, *New York*, *NY*, 2013).

## Electronic supplementary material

Supporting Information
