# Supplementary material for: Lactoferrin-derived Peptides Active towards Influenza: Identification of Three Potent Tetrapeptide Inhibitors
Source: Sci Rep. 2017 Sep 6;7:10593. doi: 10.1038/s41598-017-10492-x (PMC5587682; doi:10.1038/s41598-017-10492-x)
Supplement: Supplementary file 1 — Supporting Information [file 41598_2017_10492_MOESM1_ESM.pdf]

SUPPORTING INFORMATION

Lactoferrin-derived Peptides Active towards Influenza: Identification of Three Potent Tetrapeptide Inhibitors

Maria Carmina Scala[1§], Marina Sala[1§], Agostina Pietrantoni[2§], Antonia Spensiero[1], Simone Di Micco[1], Mariangela Agamennone[3], Alessia Bertamino[1], Ettore Novellino[4], Giuseppe Bifulco[1], Isabel M. Gomez-Monterrey[4], Fabiana Superti[2], Pietro Campiglia[1]**

[1] Department of Pharmacy, University of Salerno, Via Giovanni Paolo II 132, 84084, Fisciano, Italy

[2] National Institute of Health, Viale Regina Elena 299, 00161, Rome, Italy

[3] Department of Pharmacy, University of Chieti "G. d'Annunzio", Via dei Vestini 31, 66100, Chieti Italy

[4] Department of Pharmacy, University of Naples "Federico II", Via D. Montesano 49, 80131, Napoli, Italy

* To whom correspondence may be addressed: pcampigl@unisa.it and fabiana.superti@iss.it

[§] These authors contributed equally to this work

Table of Contents:

Title, Affiliations, Table of Contents.....S1
Docked poses of the bLf C-lobe on the HA (**Figure S1**) S2
Analytical data of peptides **4-17** (**Table S1**).....S3
HRMS spectra and HPLC chromatograms of peptide **4-17** (**Figure S2-S15**) S3
Sensorgram of peptide **4** (**Figure S16**) S24
^{1}H chemical shifts (ppm) of **1** in HFA/H_2O (600 MHz, 300 K) (**Table S2**) S25
^{1}H chemical shifts (ppm) of **13** in HFA/H_2O (600 MHz, 300 K) (**Table S3**) S26
^{1}H chemical shifts (ppm) of **14** in HFA/H_2O (600 MHz, 300 K) (**Table S4**) S26
^{1}H chemical shifts (ppm) of **15** in HFA/H_2O (600 MHz, 300 K) (**Table S5**) S27
^{1}H chemical shifts (ppm) of **17** in [D$_6$]DMSO (600 MHz, 300 K) (**Table S6**) S27
Mean values of φ, ψ and χ1 angles and αC distances relative to the most representative conformers of **1** (**Table S7**) S27
Mean values of φ, ψ and χ1 angles and αC distances relative to the most representative conformers of **13** (**Table S8**) S28

^{1}H chemical shifts (ppm) of **4** in HFA/H_2O (600 MHz, 300 K). (**Table S9**) S28
2D-NOESY spectrum of **1** HFA/H_2O solution (600 MHz, 300 K, t_{mix} = 400 ms) (**Figure S17**) S29
Ramachandran plot of NMR derived bundle of **1**, calculated by PROCHECK software (**Figure S18**) S30
2D-NOESY spectrum of **4** HFA/H_2O solution (600 MHz, 300 K, t_{mix} = 300 ms) (**Figure S19**) S31
Ramachandran plot of NMR derived bundle of **4**, calculated by PROCHECK[i] software (**Figure S20**) S32
2D-NOESY spectrum of **13** HFA/H_2O solution (600 MHz, 300 K, t_{mix} = 500 ms) (**Figure S21**) S33
Ramachandran plot of NMR derived bundle of **13**, calculated by PROCHECK[i] software (**Figure S22**) S34
References...S34

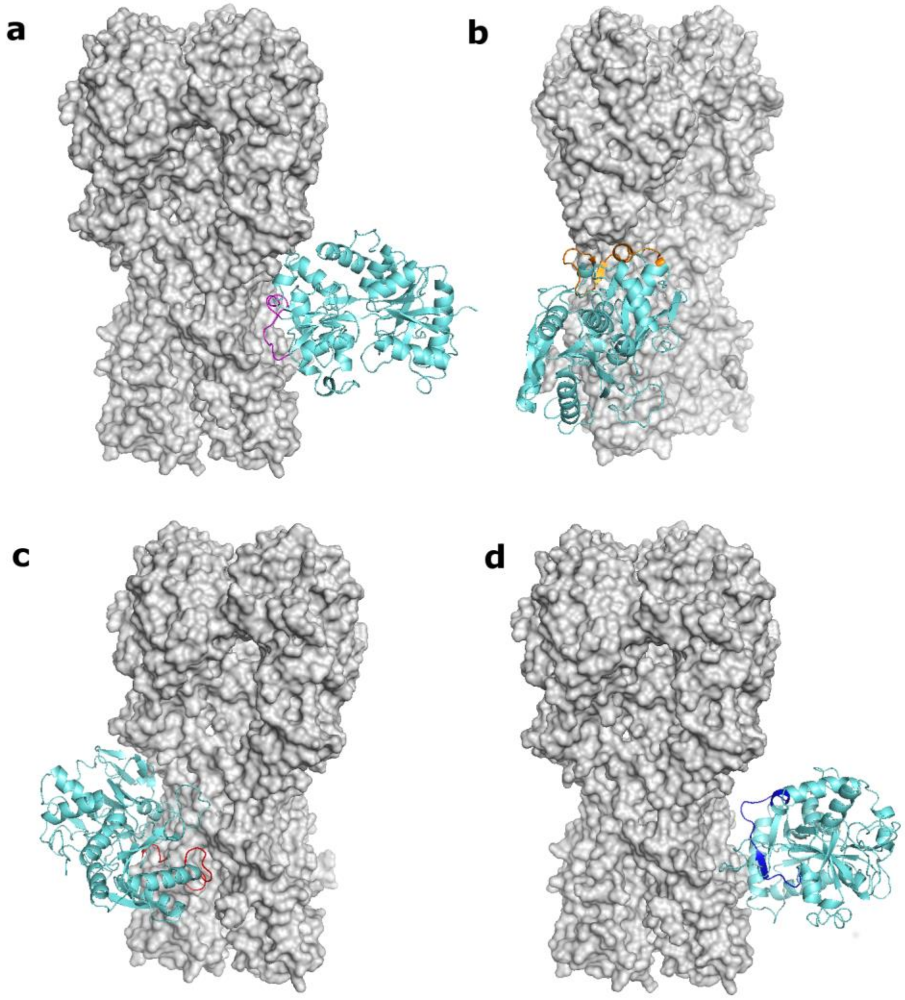

Figure S1. Docked poses of the bLf C-lobe (cyan cartoon) on the HA (grey surface) highlighting the potential role of selected loop sequences. Selected sequences are colored: a) peptide **4** (purple); b) peptide **5** (orange); c) peptides **7** and **8** (red); d) peptide **9** (blue).

Table S1. Analytical data of peptides **4-17**.

Pep.	Sequence	HPLC k'[a]	HRMS
**4**	KANEGLTWNSLKDK	6.47	1602.85134
**5**	TGSCAFDEFFSQSCAPGADPKSR	6.89	2408.02628
**6**	TNGESTADWAKN	5.07	1292.58779
**7**	GKNGKNCPDKFC	4.26	1308.58370
**8**	KSETKN	5.22	706.37420
**9**	NDNTECLAKLGGRPTYEE	5.76	2009.92731
**10**	NRKSSKHSSLDCVLRPTEGY	6.08	2278.15056
**11**	NRKSSKHSSLDCVLRP	5.71	1825.98013
**12**	SKHSSLDCVLRPTEGY	2.45	1791.87152
**13**	SLDCVLRP	6.67	901.49417
**14**	VLRP	5.70	525.35082
**15**	SLDC	3.93	477.18970
**16**	SKHSSLDC	4.14	876.38864
**17**	SKHS	4.68	499.26235

[a] k'=[(peptide retention time-solvent retention time)/solvent retention time].

Supplementary figures of Mass spectrometry and HPLC of peptides used in the study

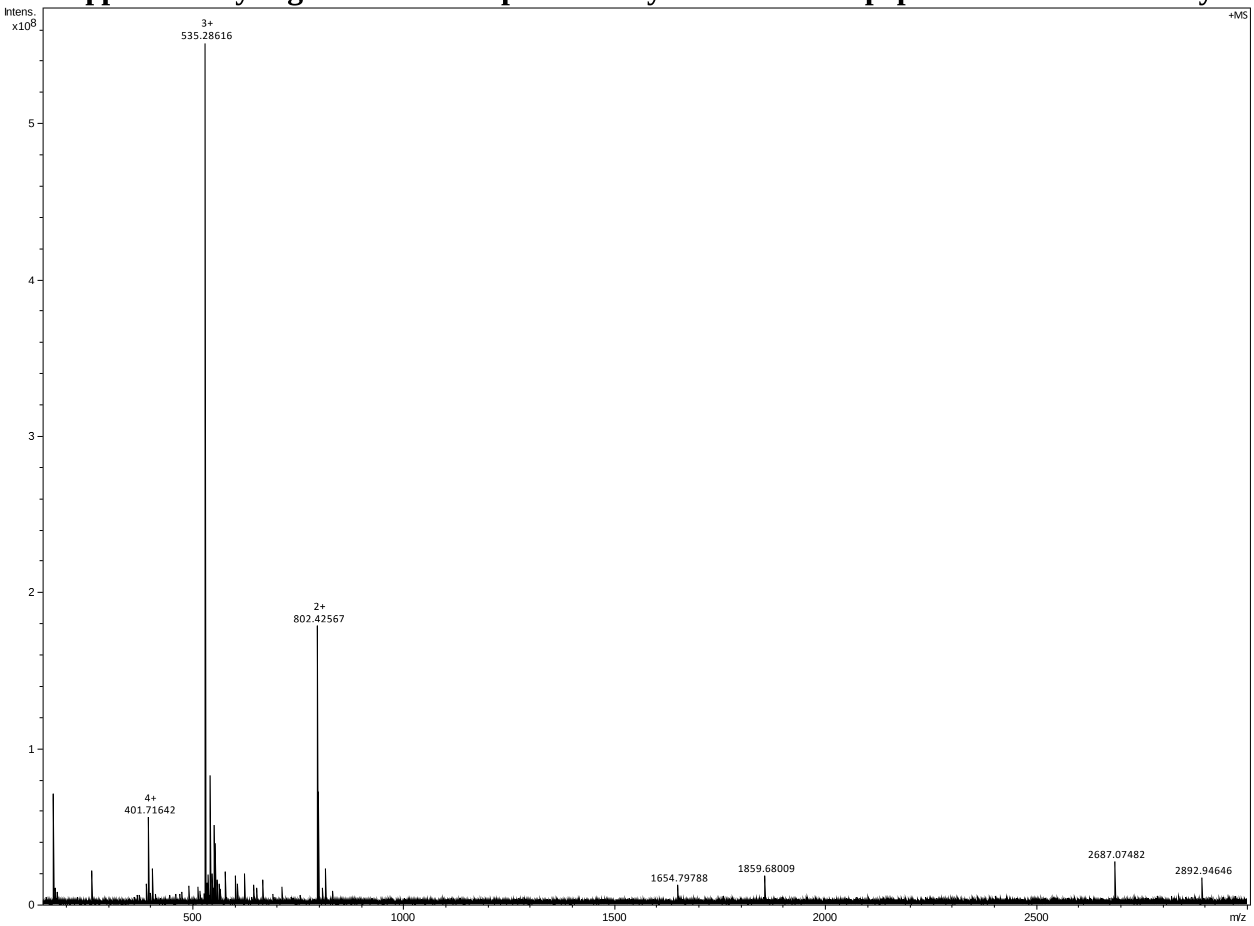

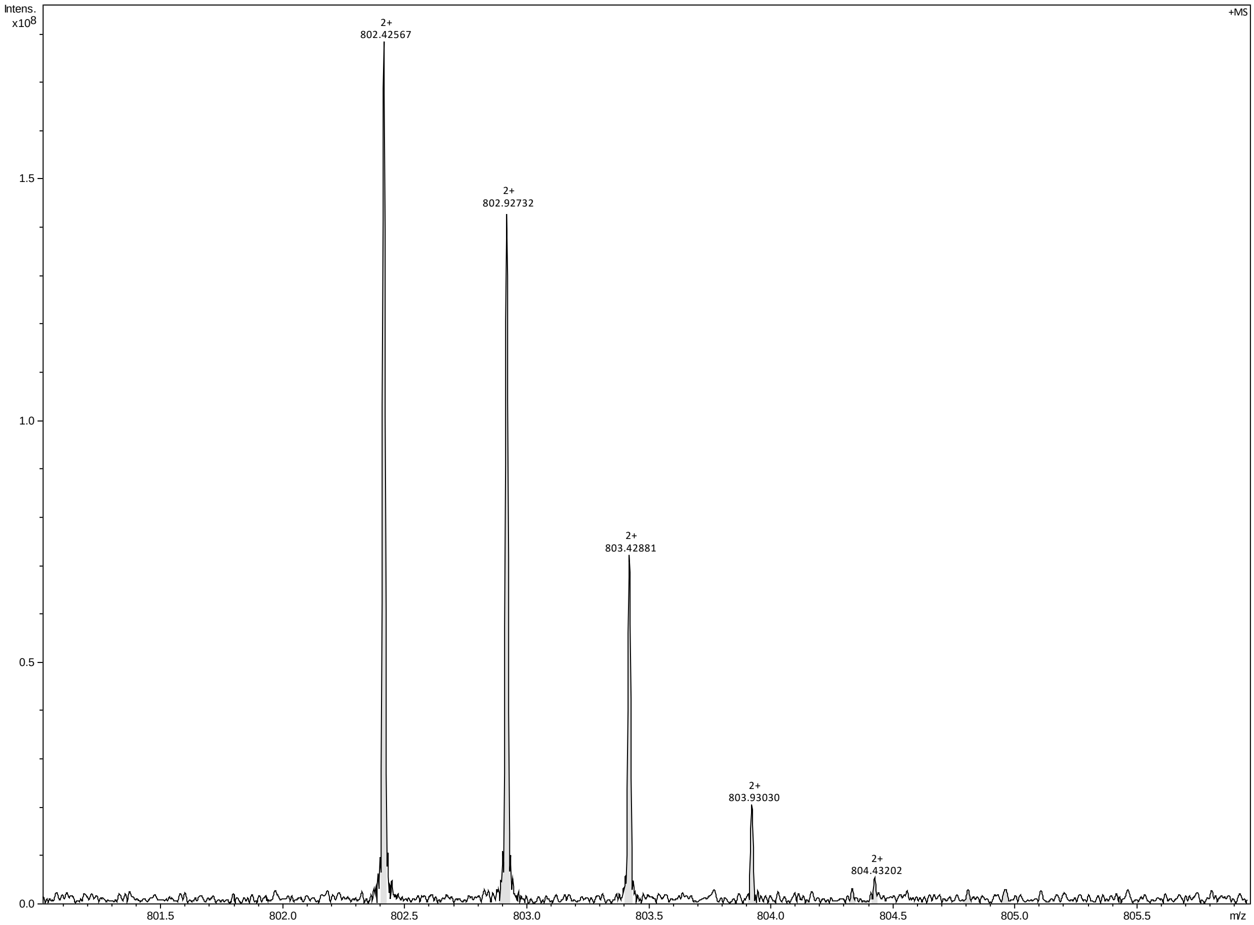

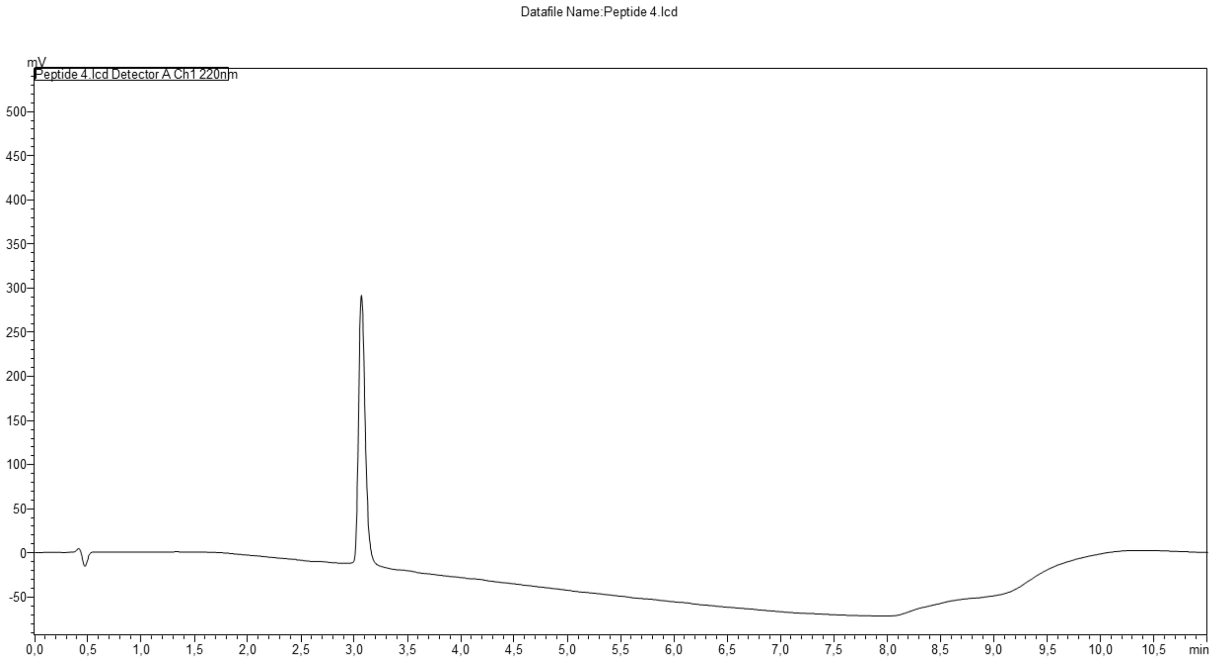

Figure S2. HR-ESI-MS of Peptide **4** ion $[M+H]^{2+}$ and analytical HPLC trace at 220 nm.

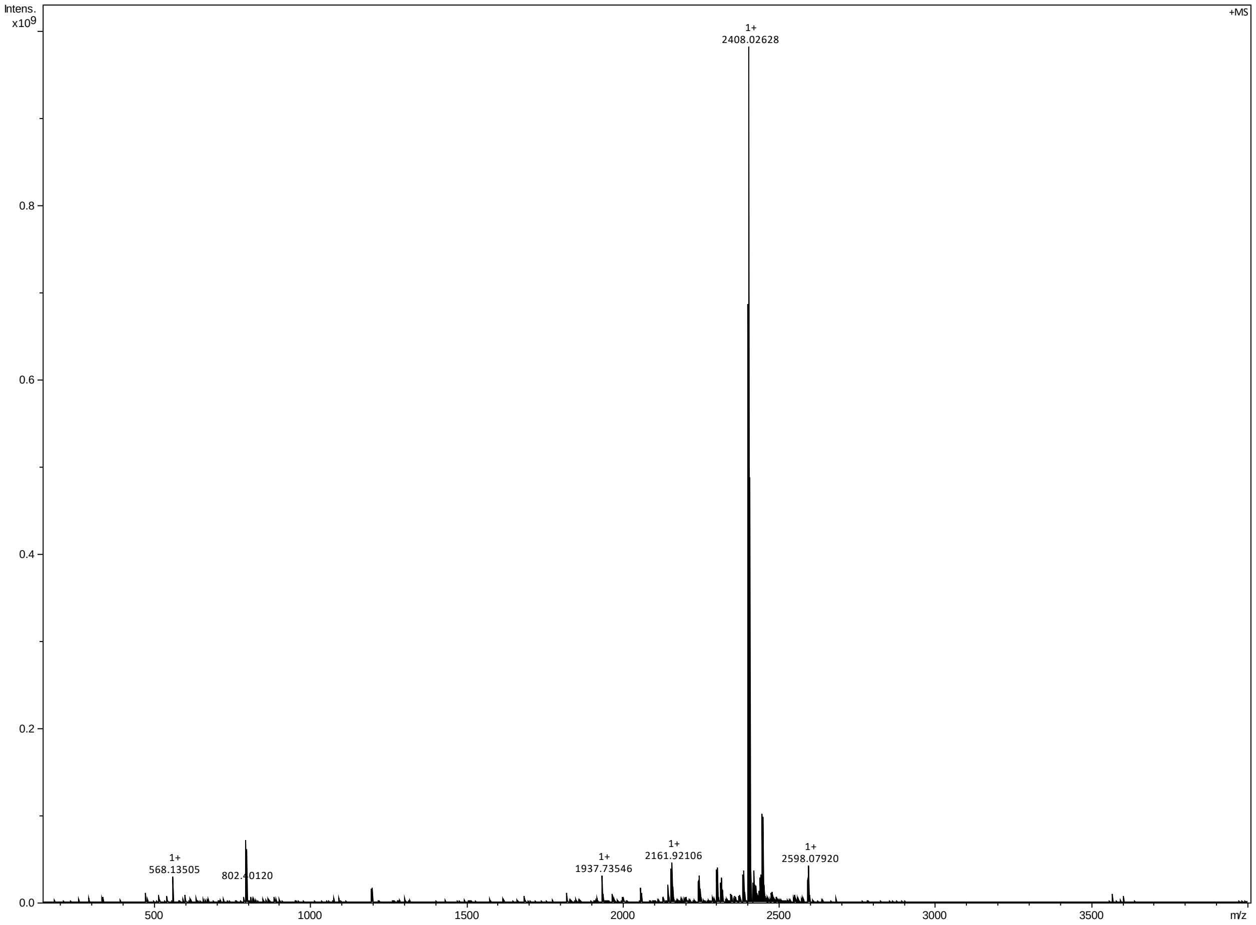
Intens.
x10^9
+MS
1+
2408.02628
1+
568.13505
802.40120
1+
1937.73546
1+
2161.92106
1+
2598.07920
0.0
0.2
0.4
0.6
0.8
500
1000
1500
2000
2500
3000
3500
m/z

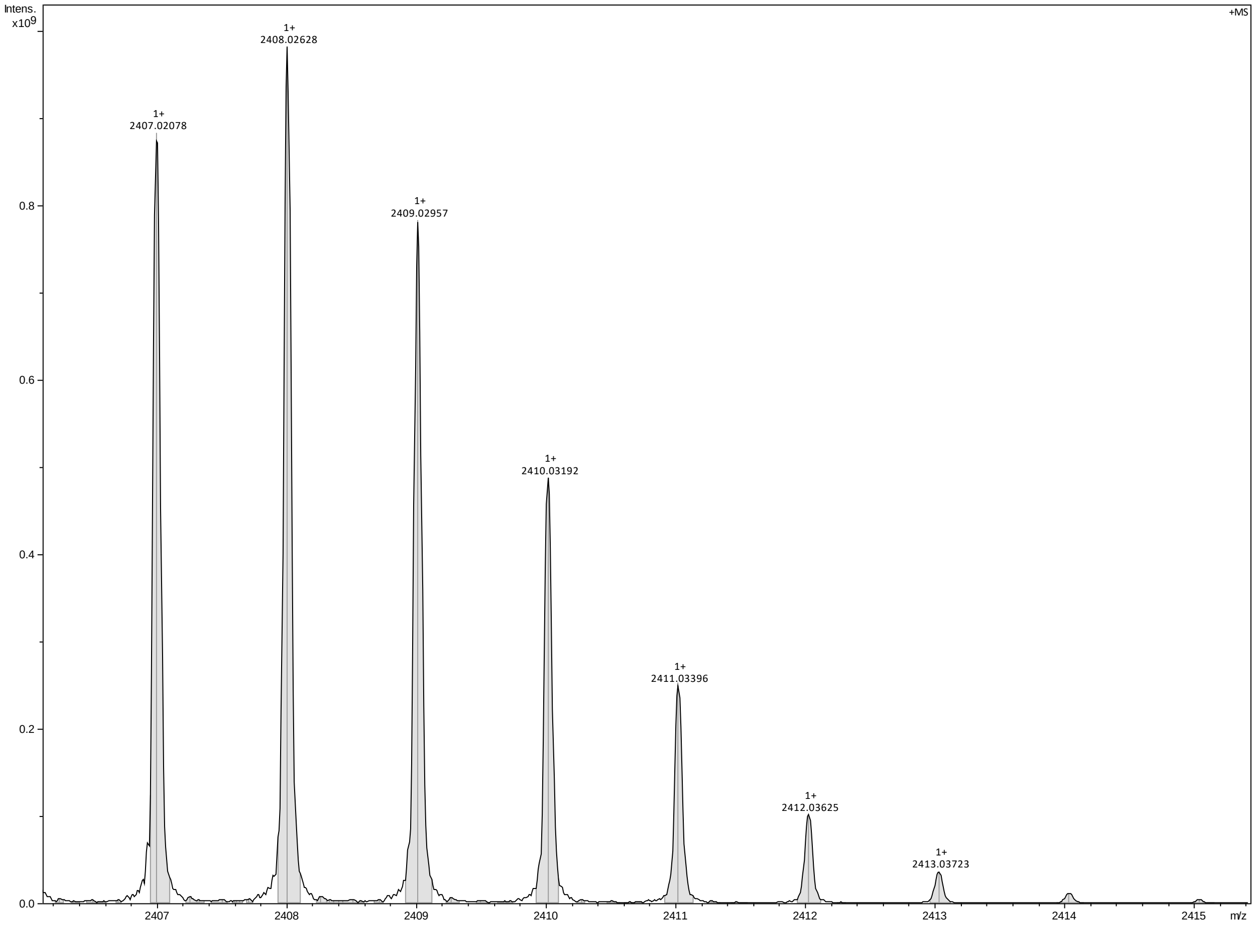
Intens.
x10^9
+MS
1+
2408.02628
1+
2407.02078
1+
2409.02957
1+
2410.03192
1+
2411.03396
1+
2412.03625
1+
2413.03723
0.0
0.2
0.4
0.6
0.8
2407
2408
2409
2410
2411
2412
2413
2414
2415
m/z

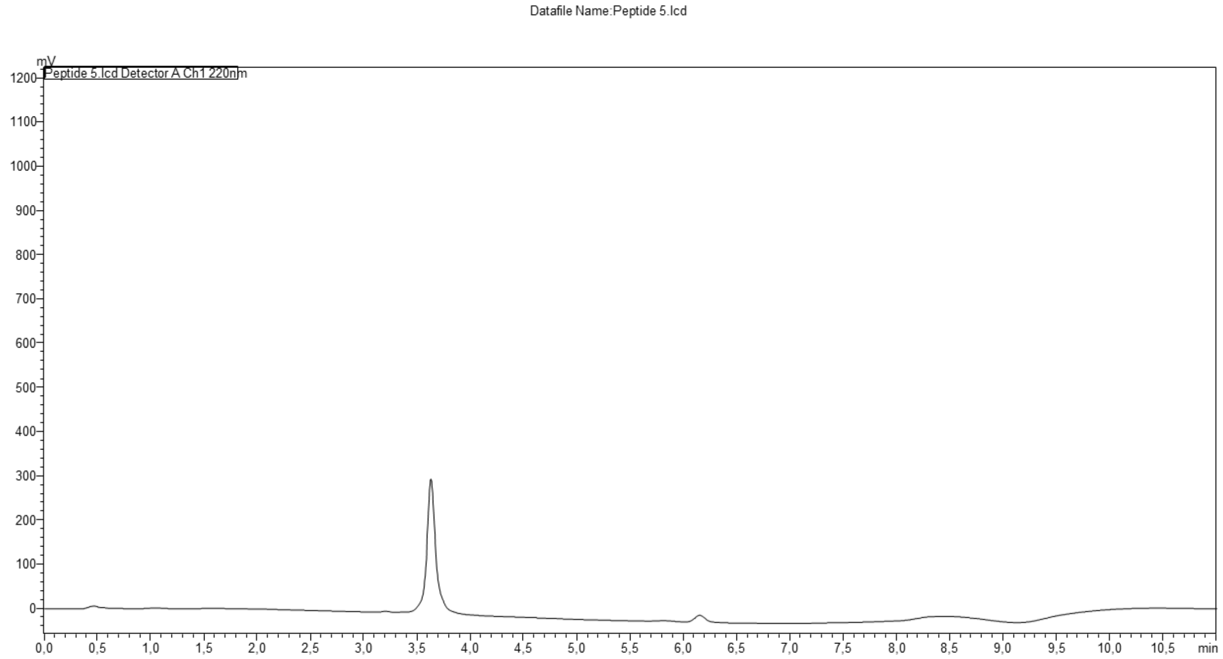

Figure S3. HR-MALDI-MS of Peptide **5** ion $[M+H]^+$ and analytical HPLC trace at 220 nm.

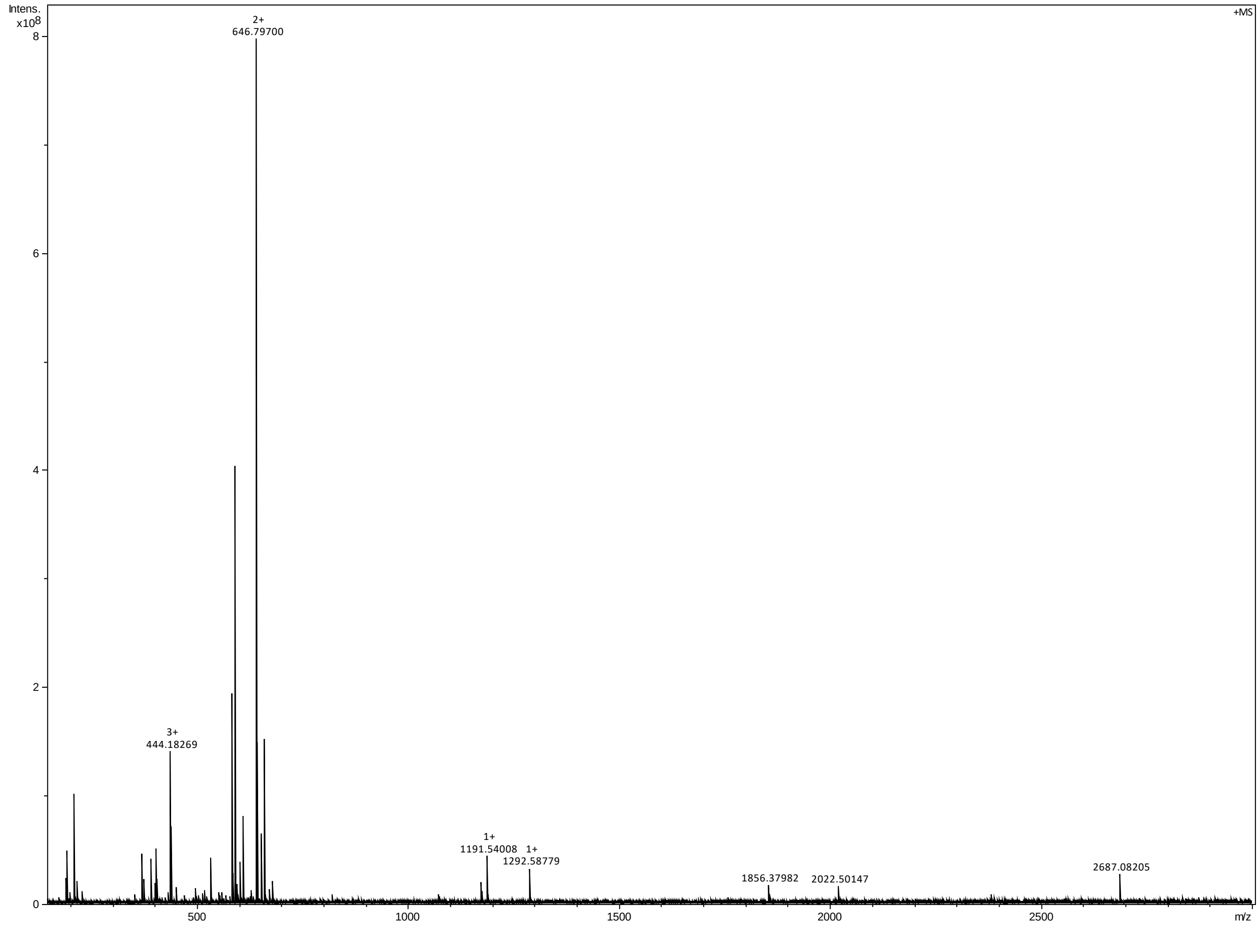

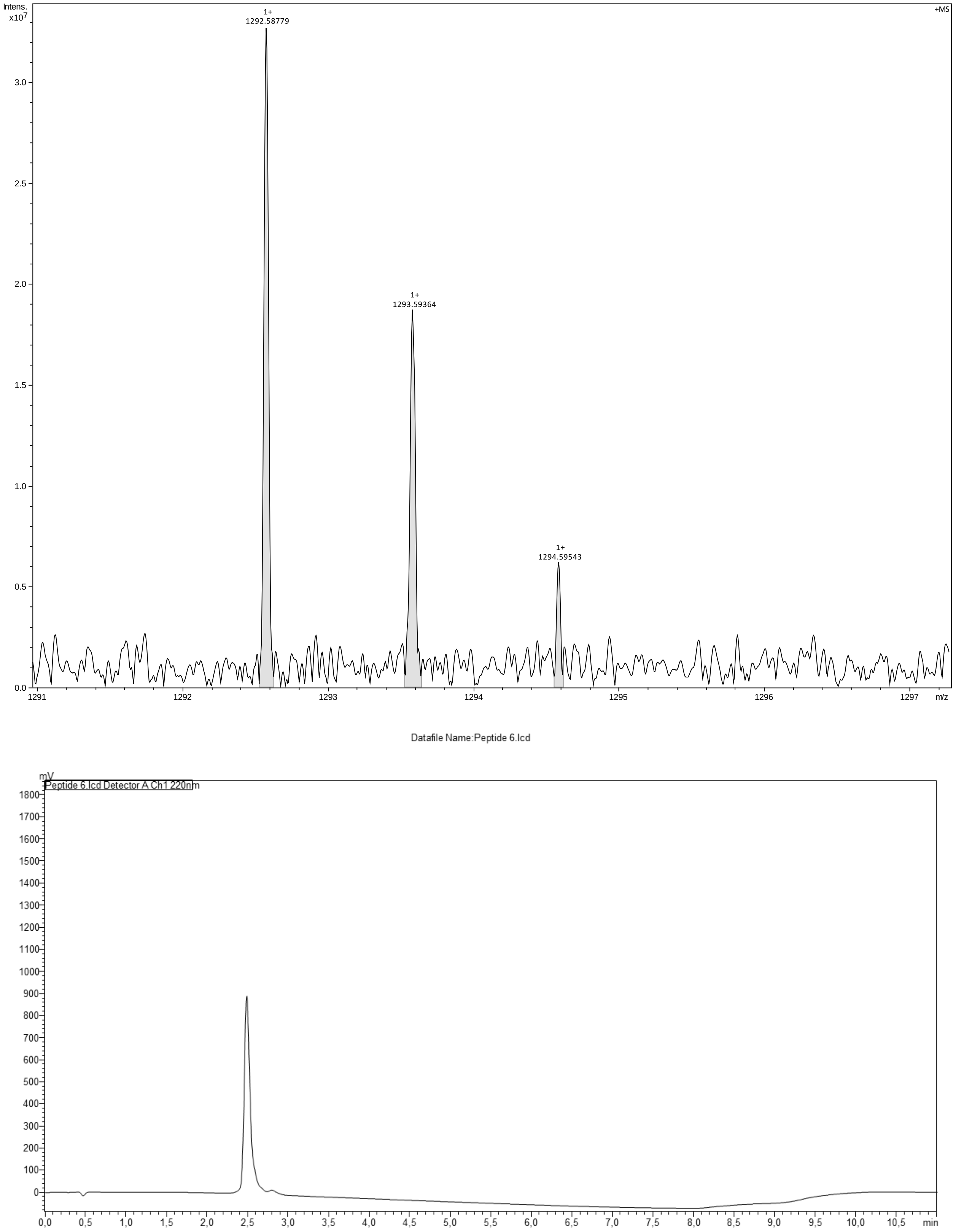

Figure S4. HR-ESI-MS of Peptide **6** ion $[M+H]^{+}$ and analytical HPLC trace at 220 nm.

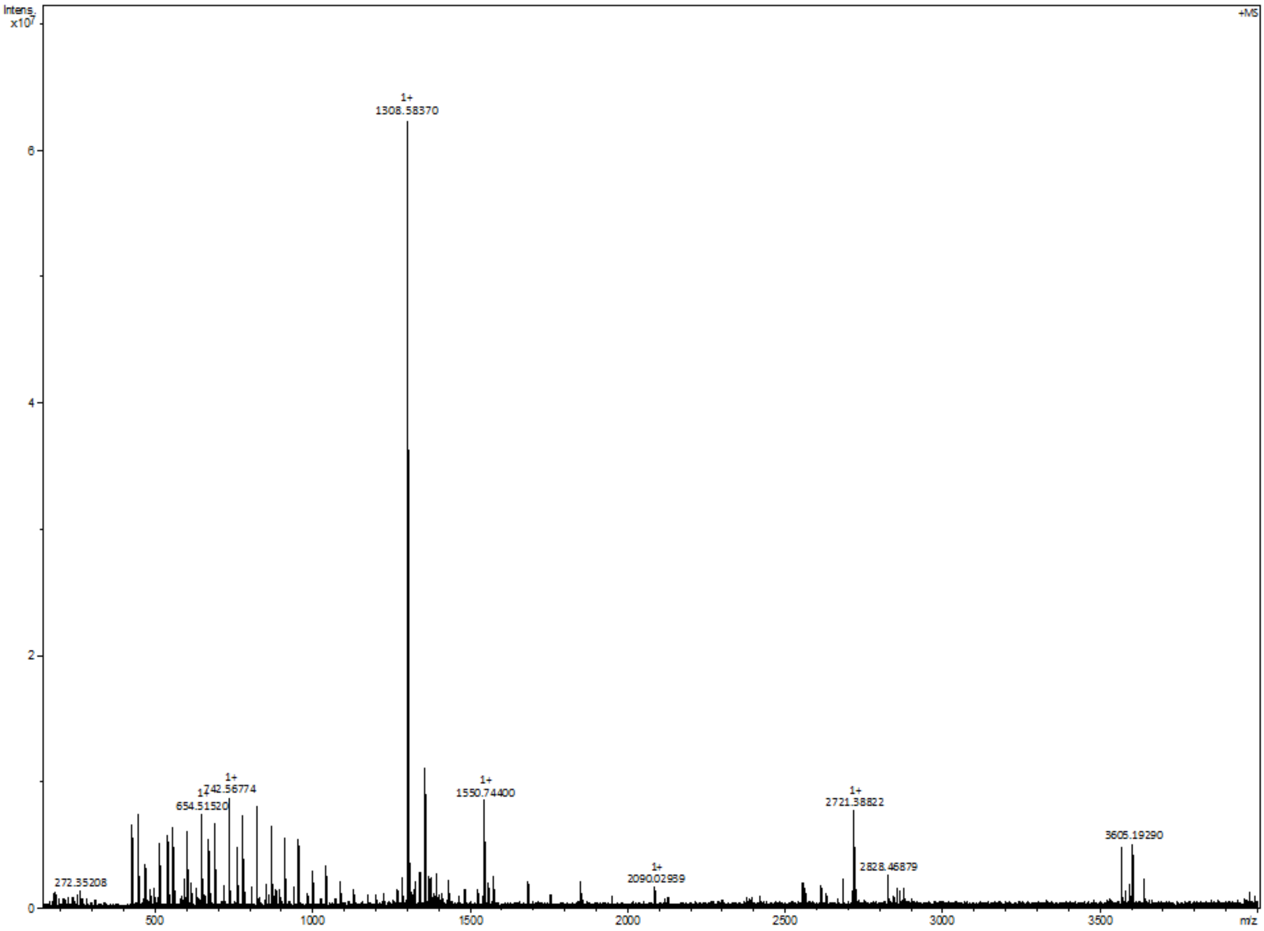
Intens. x10^7
+MS
1+
1308.58370
1+
742.56774
1+
654.51520
1+
1550.74400
1+
2721.38822
3605.19290
2828.46879
1+
2090.02939
272.35208
m/z

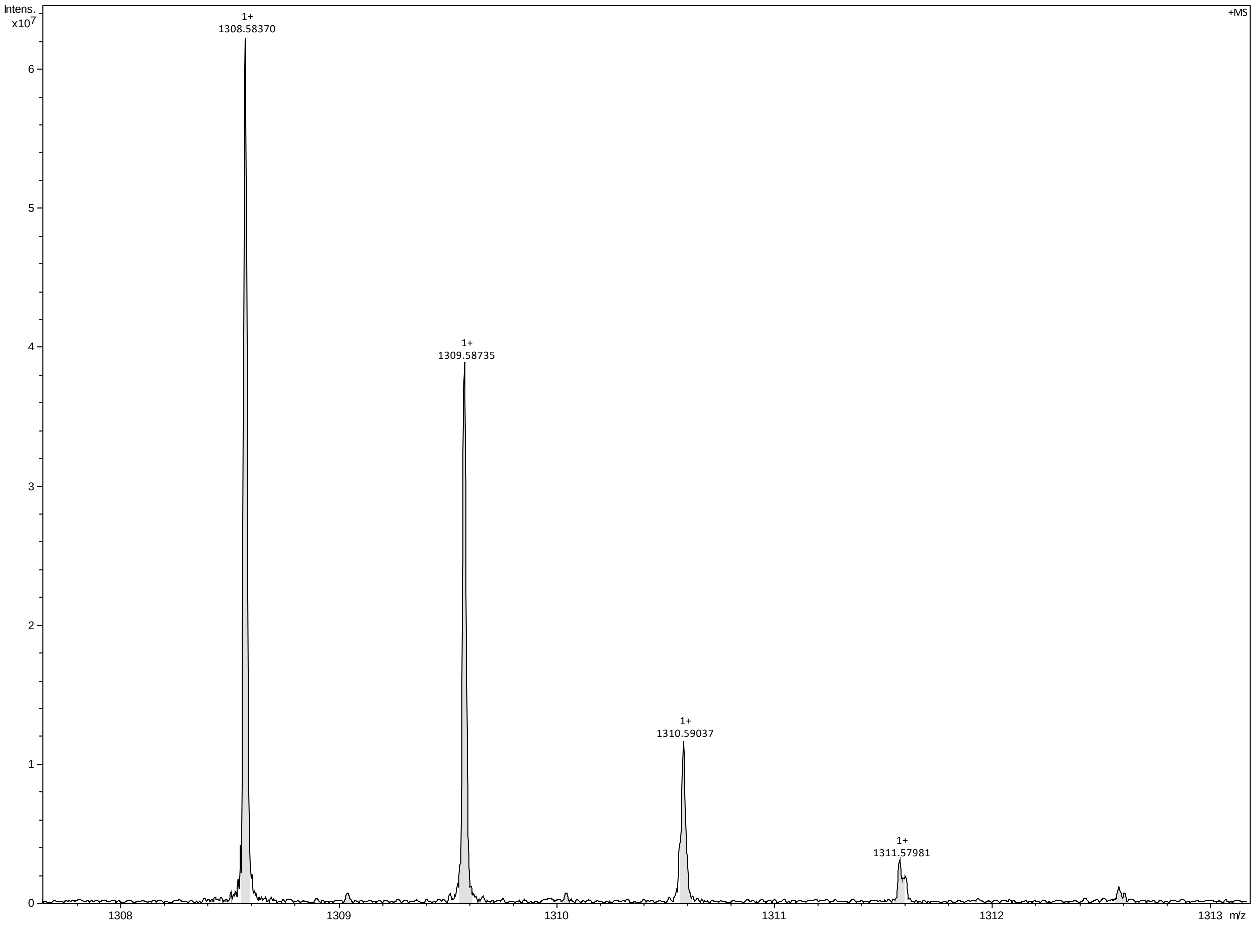
Intens. x10^7
+MS
1+
1308.58370
1+
1309.58735
1+
1310.59037
1+
1311.57981
m/z

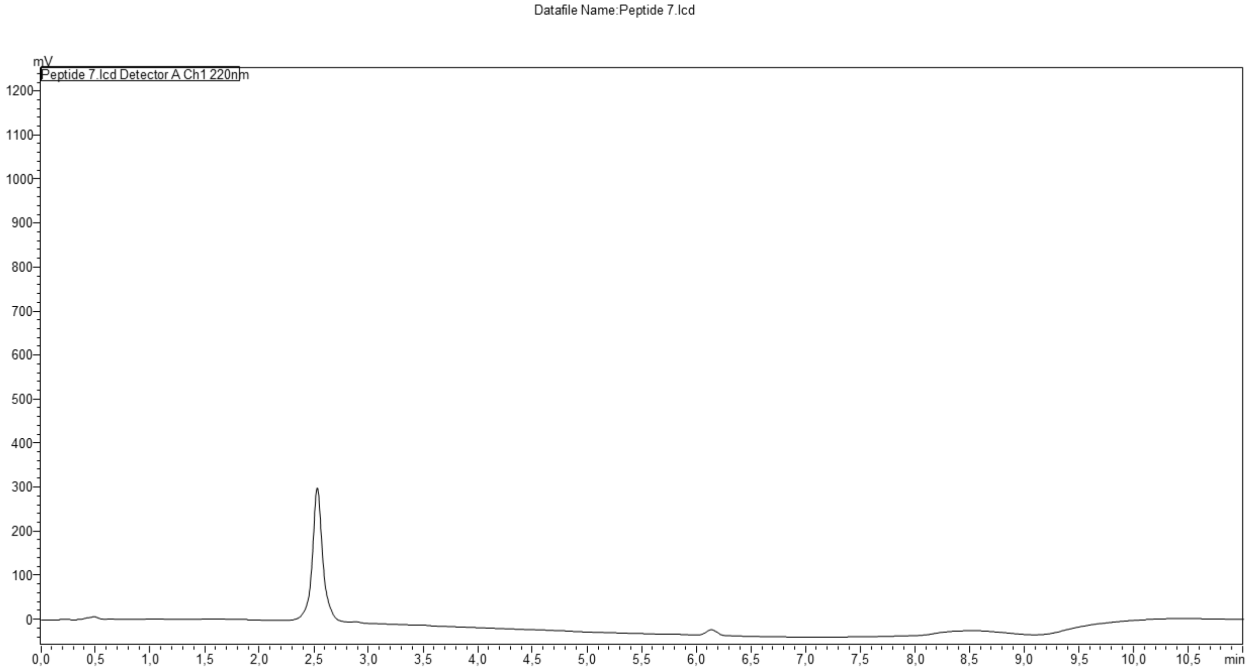

Figure S5. HR-MALDI-MS of Peptide **7** ion $[M+H]^+$ and analytical HPLC trace at 220 nm.

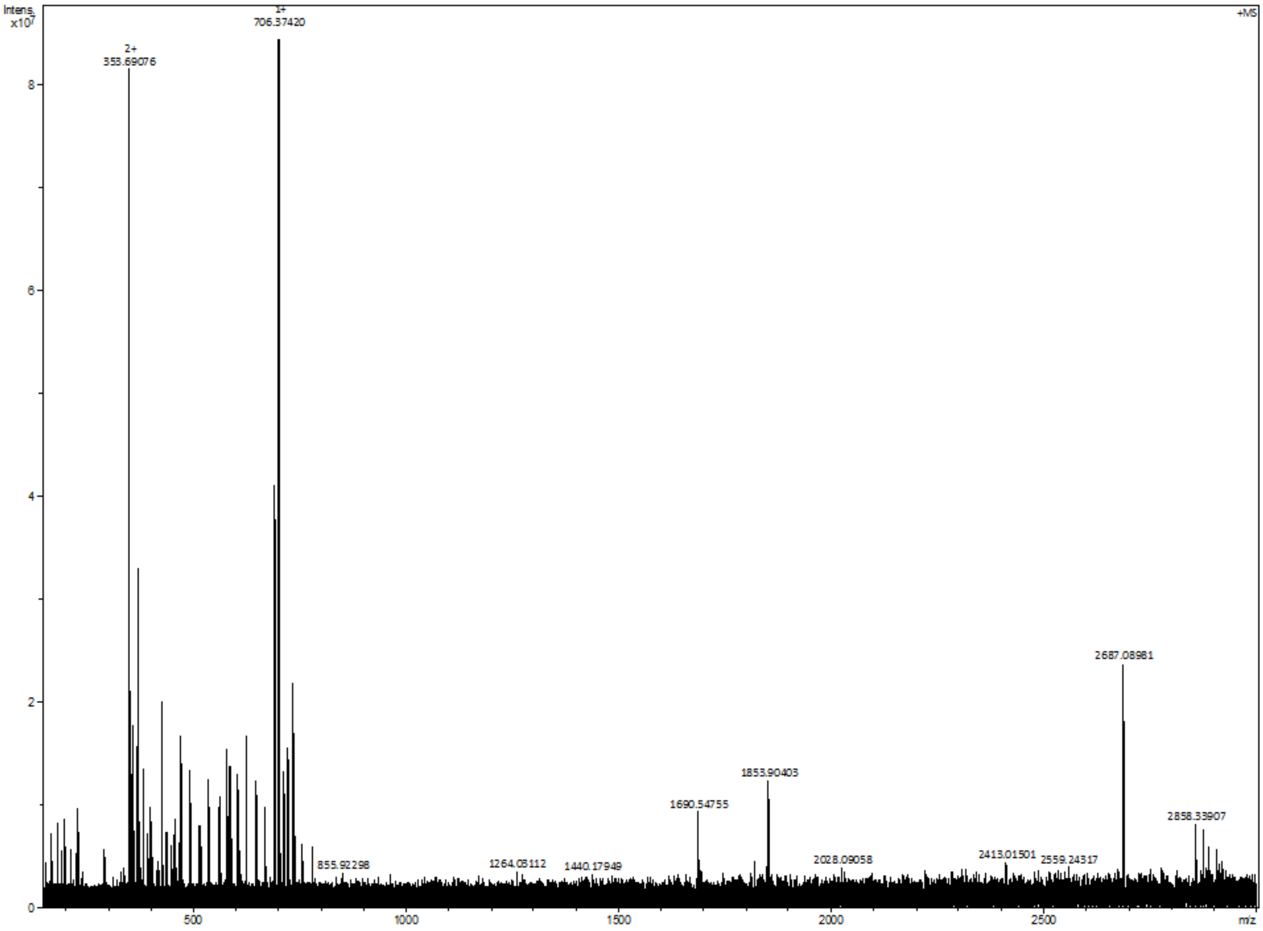

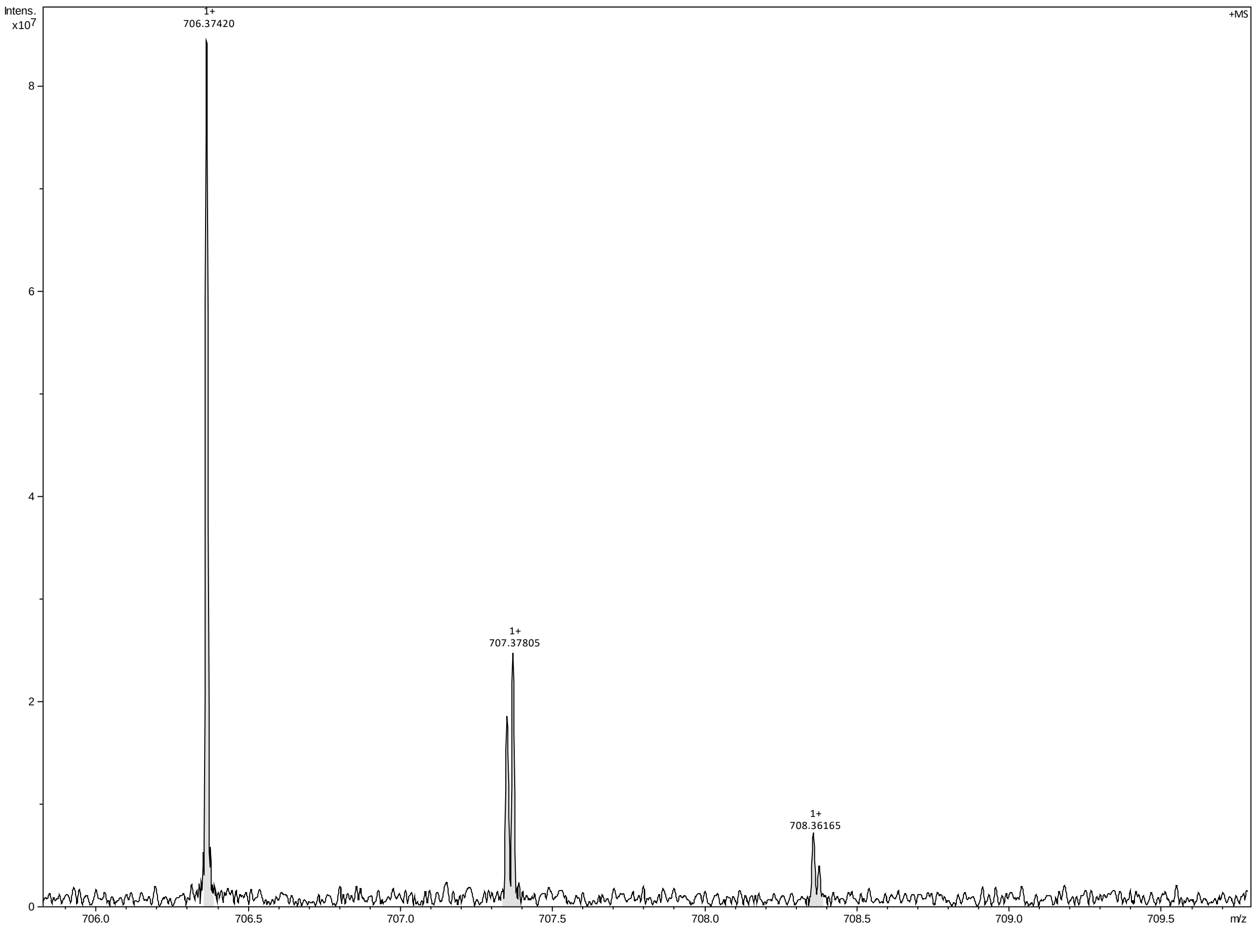

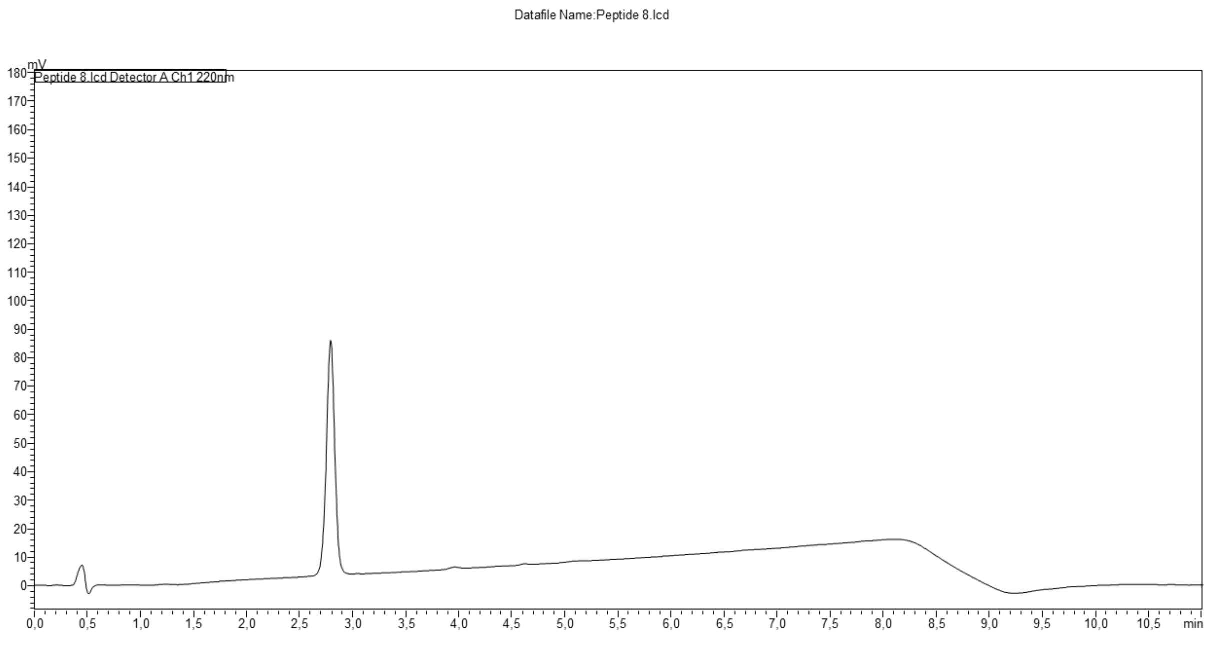

Figure S6. HR-ESI-MS of Peptide **8** ion $[M+H]^+$ and analytical HPLC trace at 220 nm.

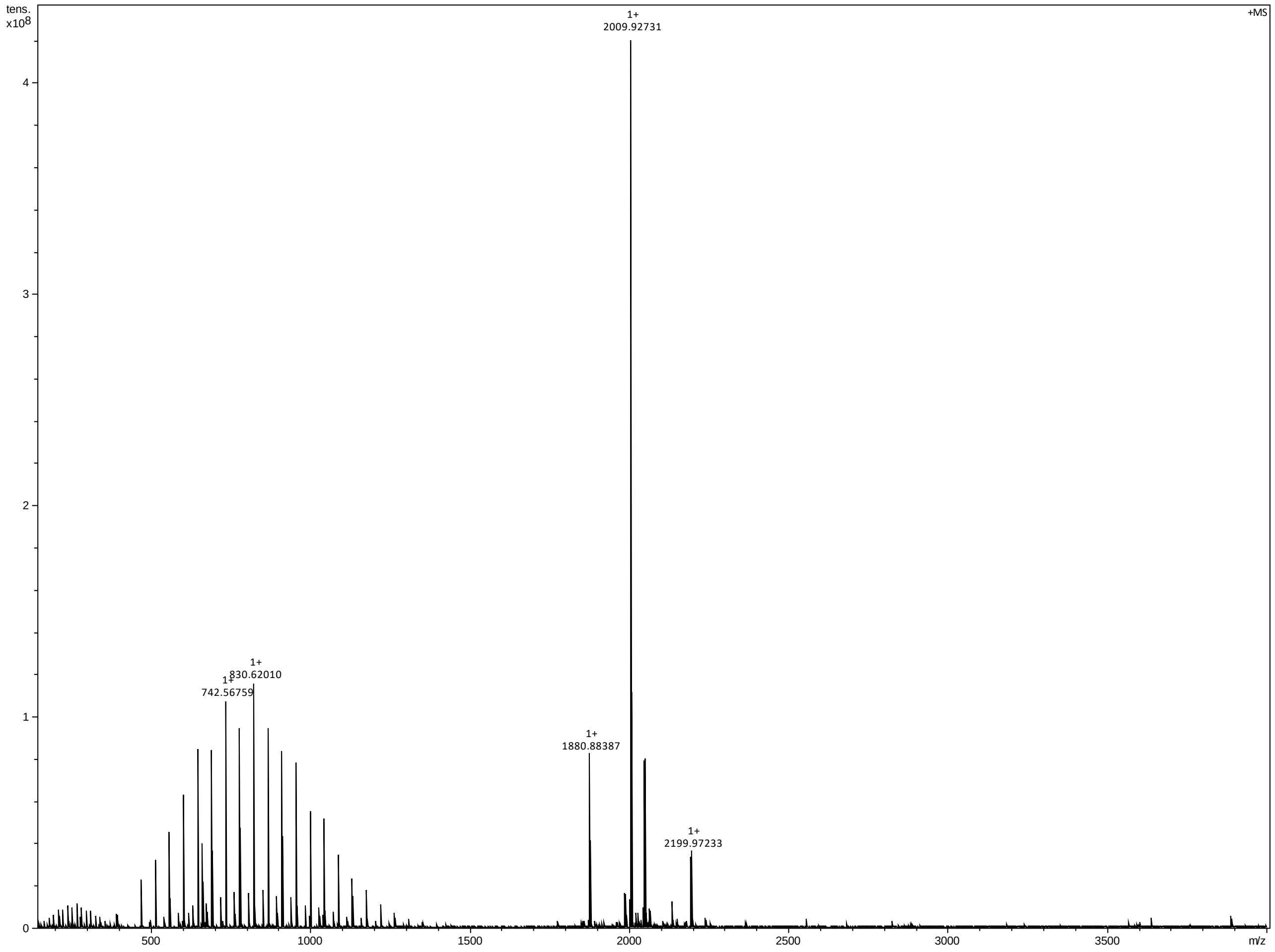
tens. x10^8
+MS
1+
2009.92731
1+
830.62010
1+
742.56759
1+
1880.88387
1+
2199.97233
4
3
2
1
0
500
1000
1500
2000
2500
3000
3500
m/z

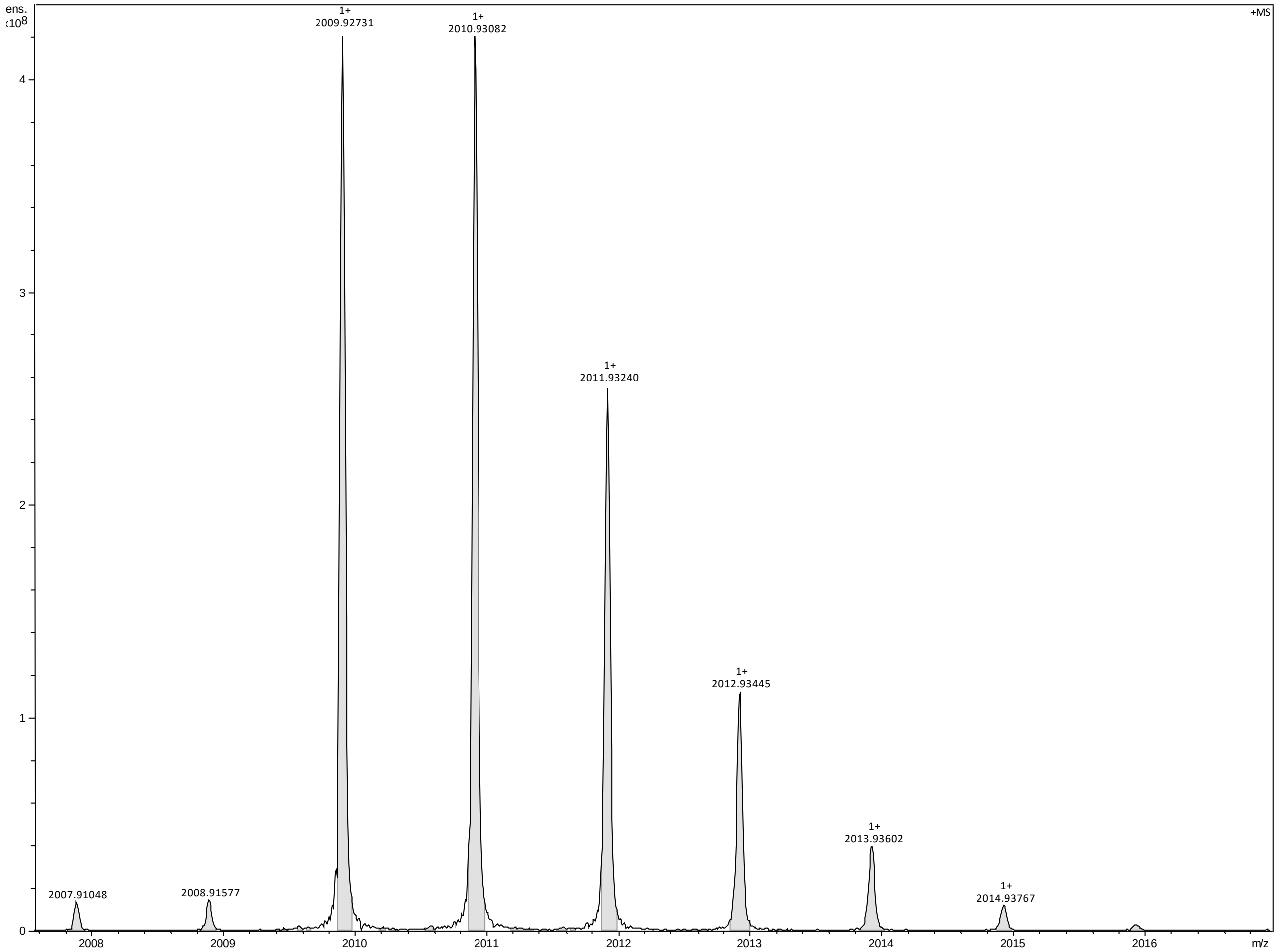

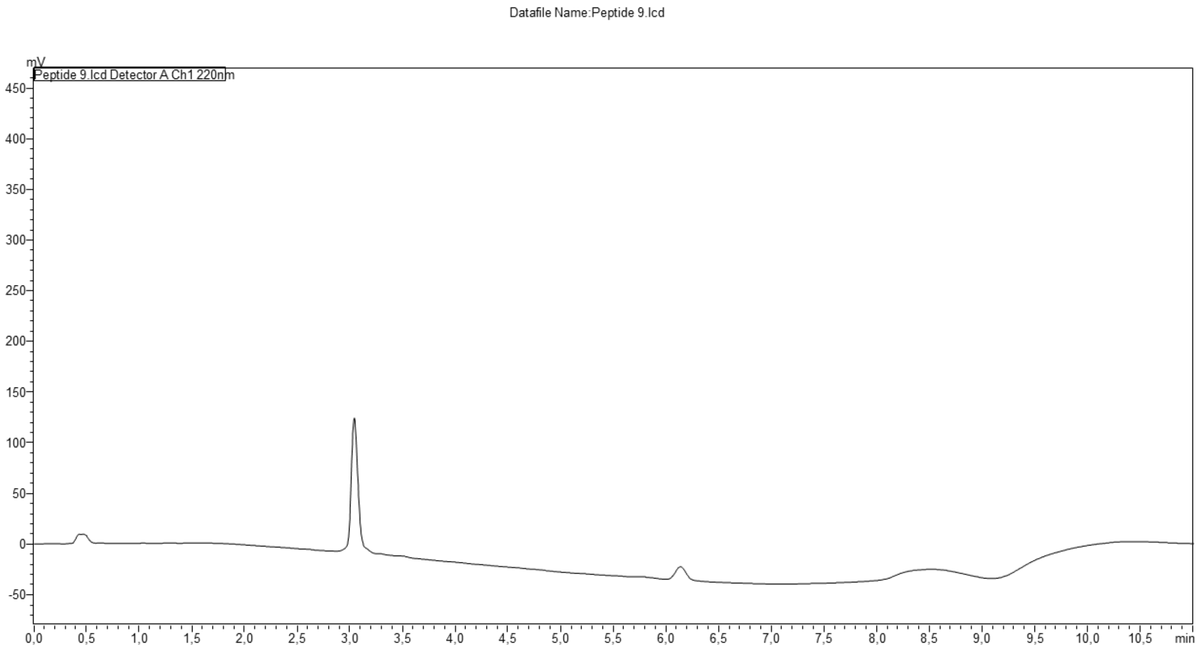

Figure S7. HR-MALDI-MS of Peptide **9** ion $[M+H]^+$ and analytical HPLC trace at 220 nm.

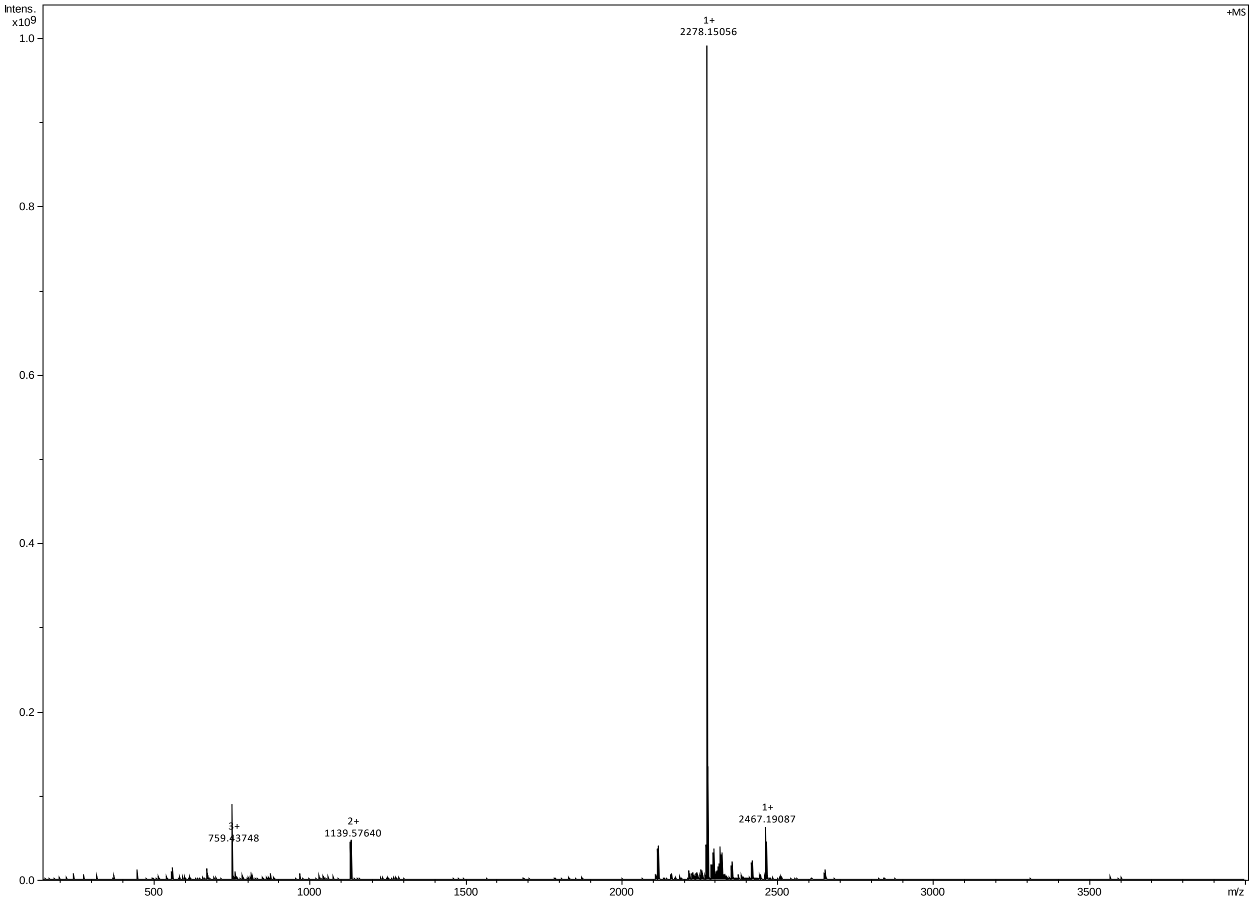
Intens.
x10^9
+MS
1+
2278.15056
3+
759.43748
2+
1139.57640
1+
2467.19087
m/z

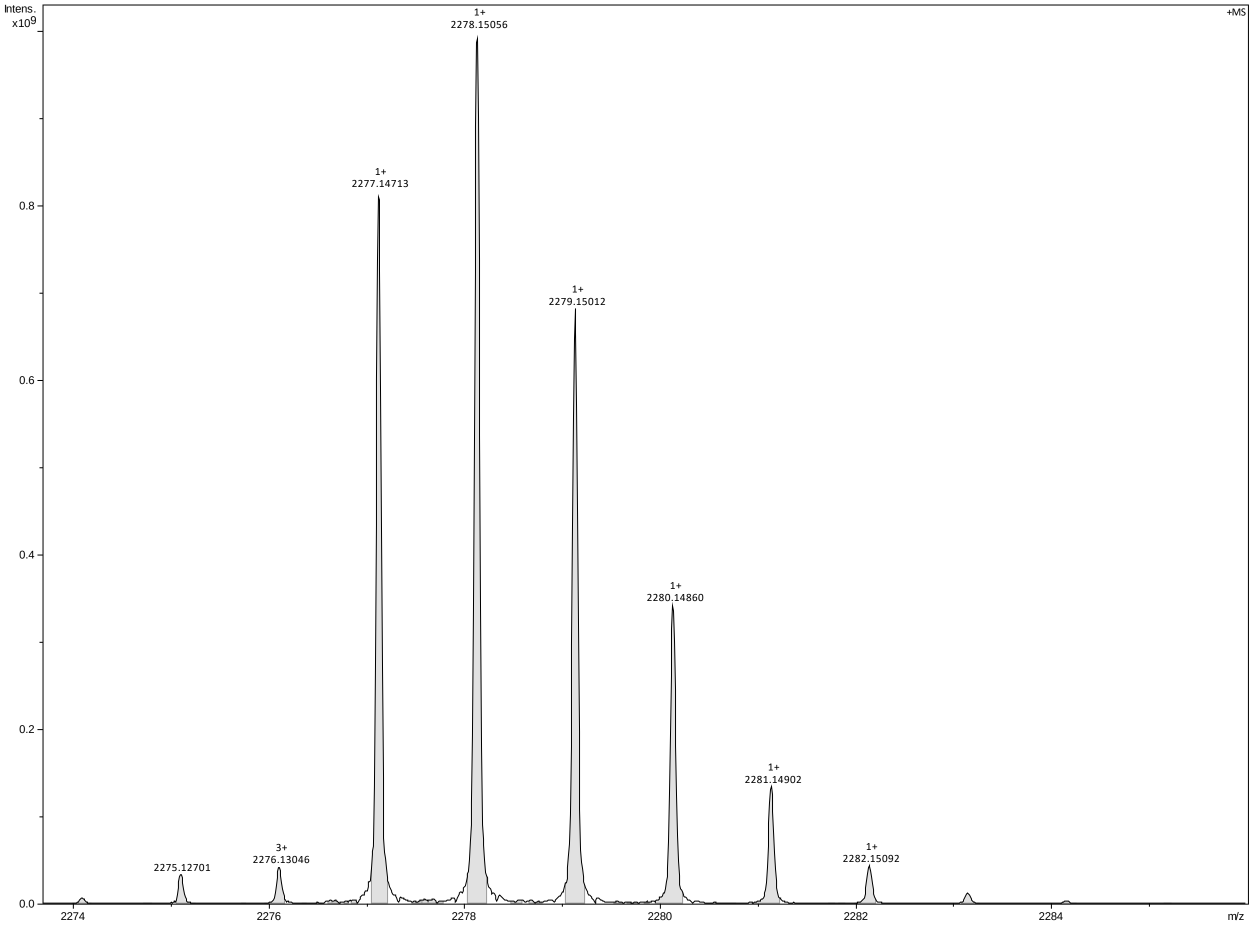
Intens.
x10^9
+MS
1+
2278.15056
1+
2277.14713
1+
2279.15012
1+
2280.14860
1+
2281.14902
2275.12701
3+
2276.13046
1+
2282.15092
m/z

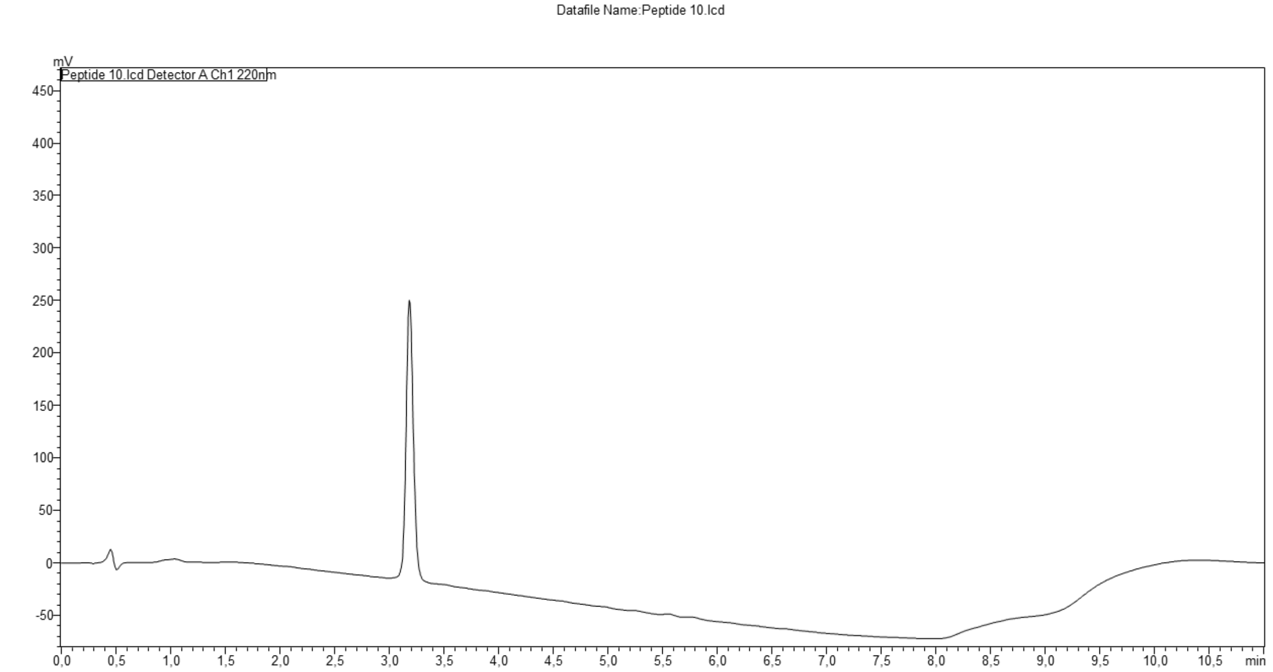

Figure S8. HR-MALDI-MS of Peptide **10** ion $[M+H]^+$ and analytical HPLC trace at 220 nm.

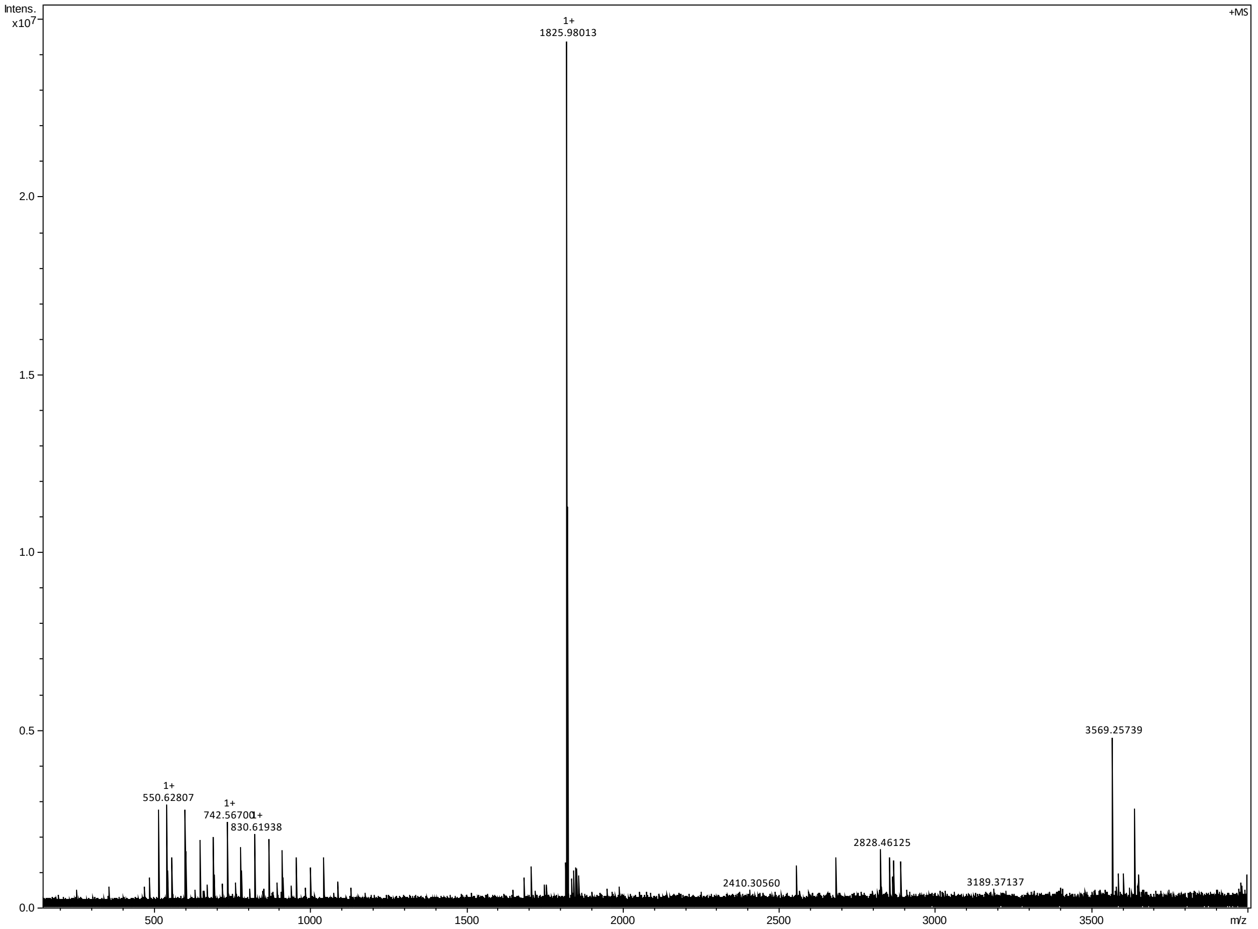

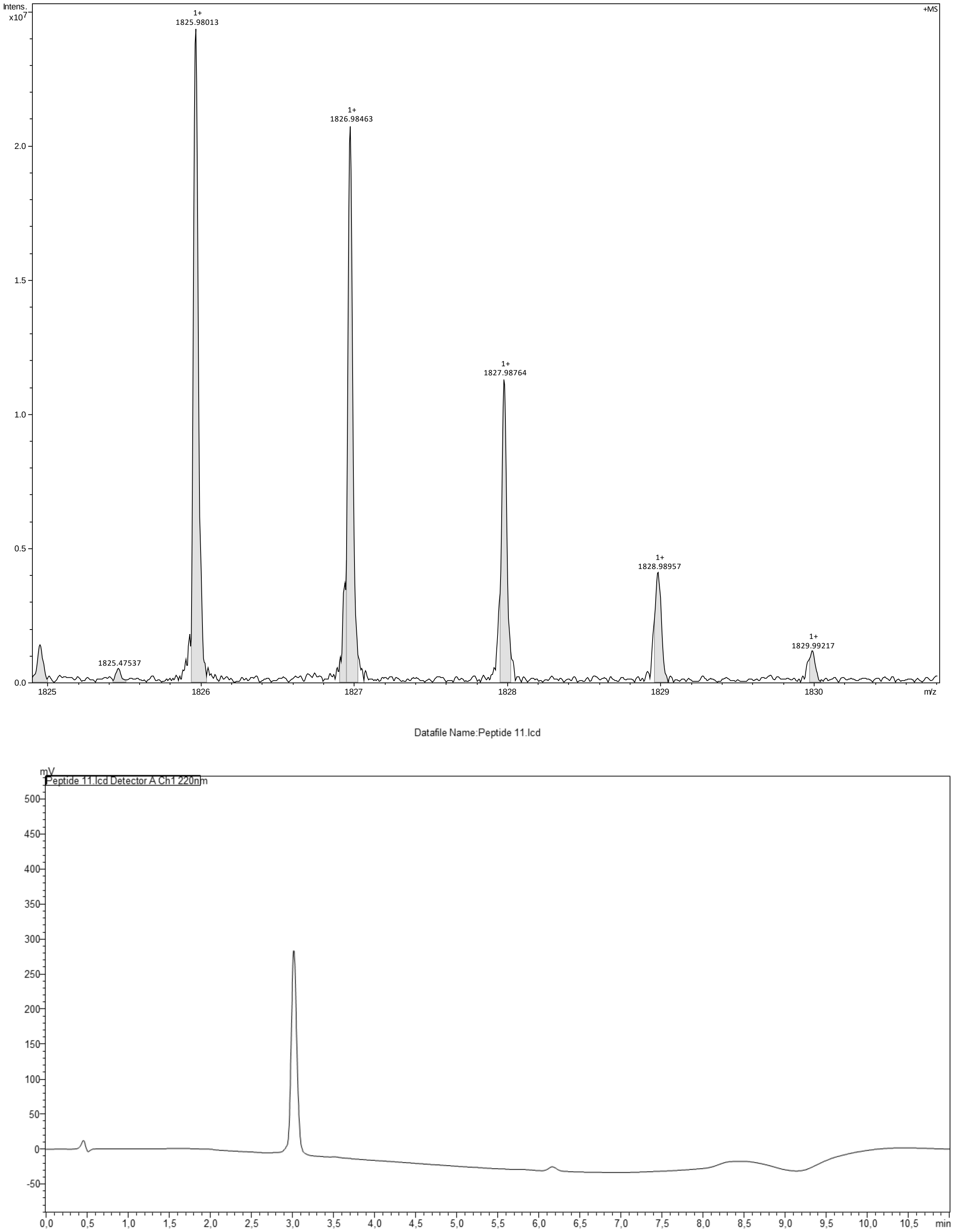

Figure S9. HR-MALDI-MS of Peptide **11** ion $[M+H]^+$ and analytical HPLC trace at 220 nm.

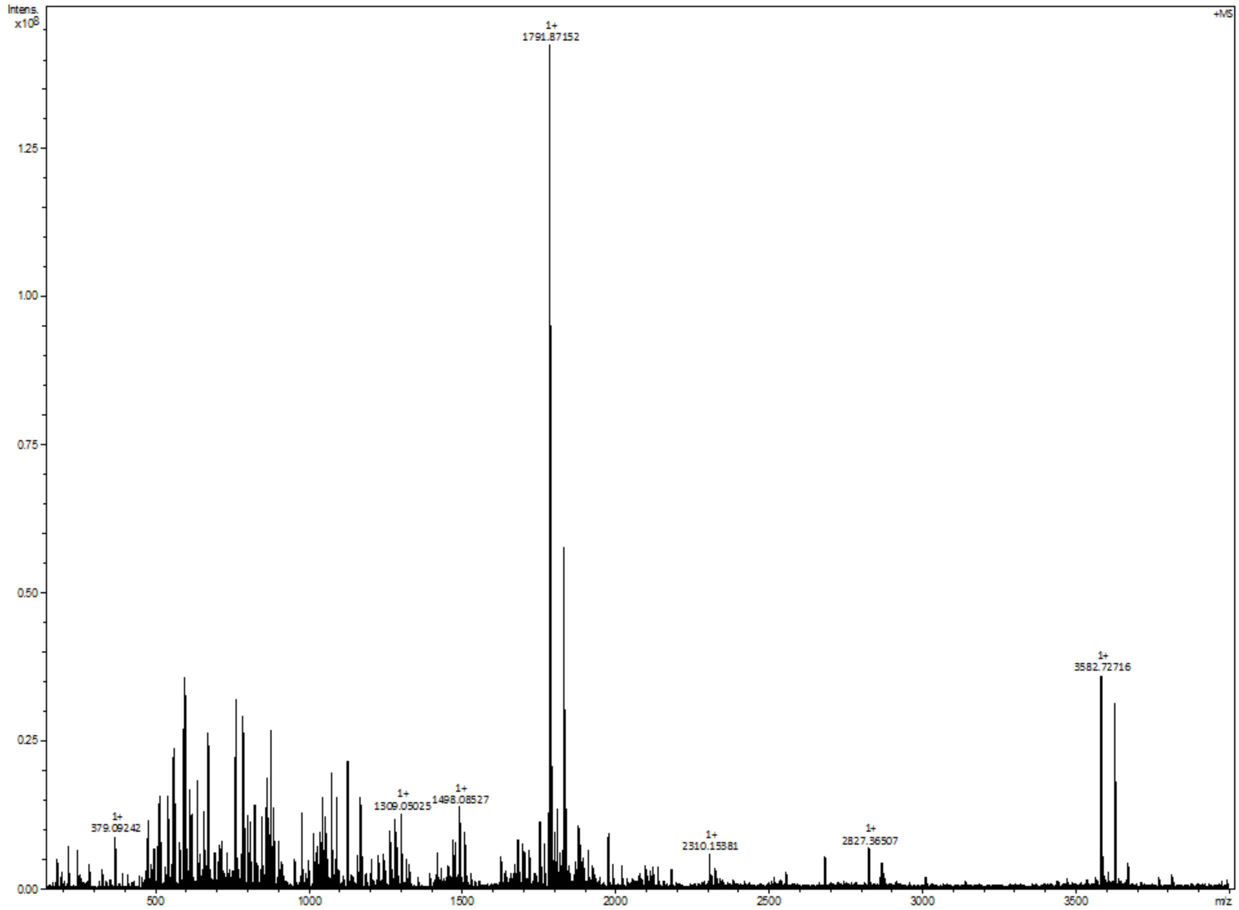
Intens.
x10^8
+MS
1+
1791.87152
1+
3582.72716
1+
379.09242
1+
1309.05025
1+
1498.08527
1+
2310.15381
1+
2827.36507
m/z

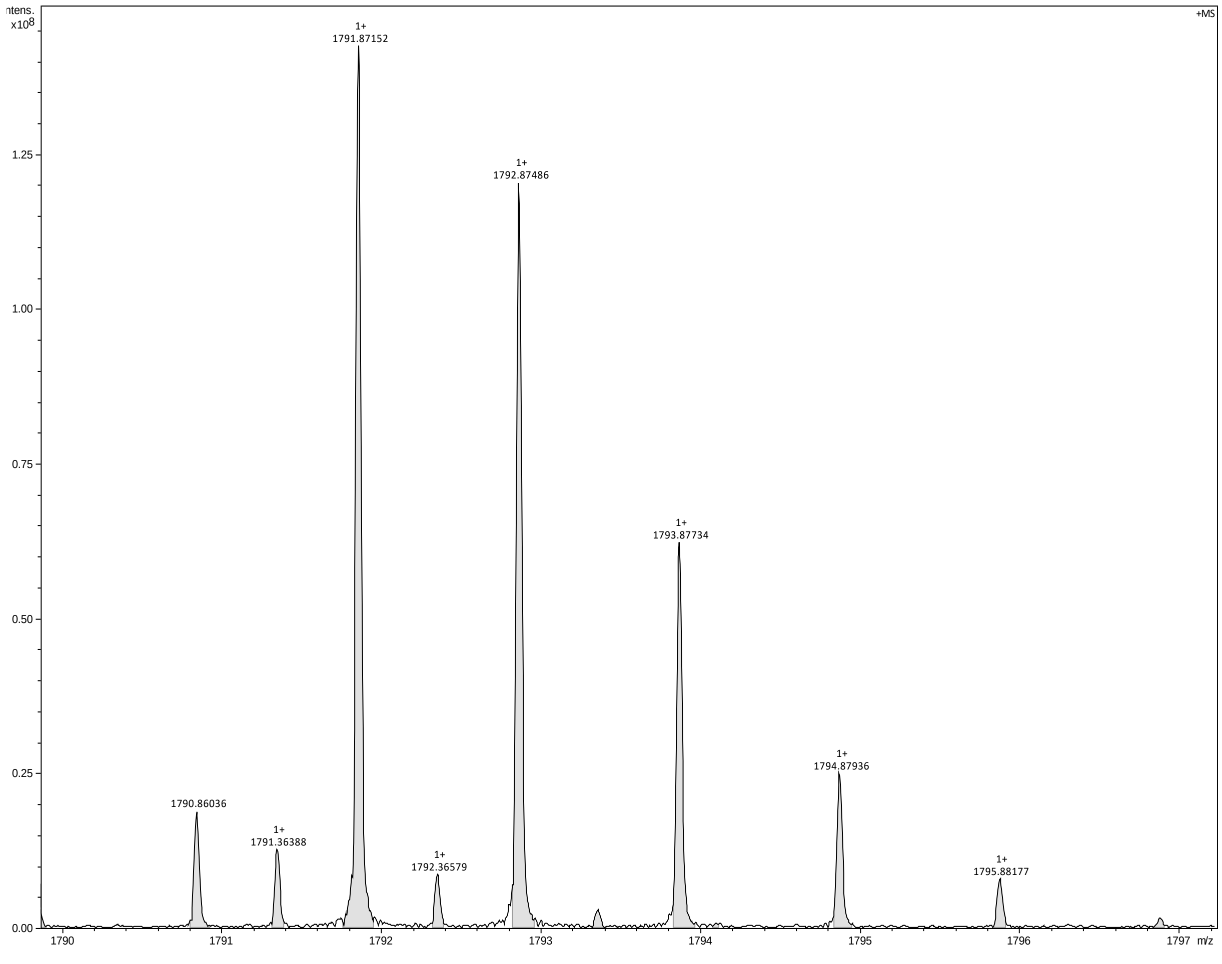
Intens.
x10^8
+MS
1+
1791.87152
1+
1792.87486
1+
1793.87734
1+
1794.87936
1790.86036
1+
1791.36388
1+
1792.36579
1+
1795.88177
m/z

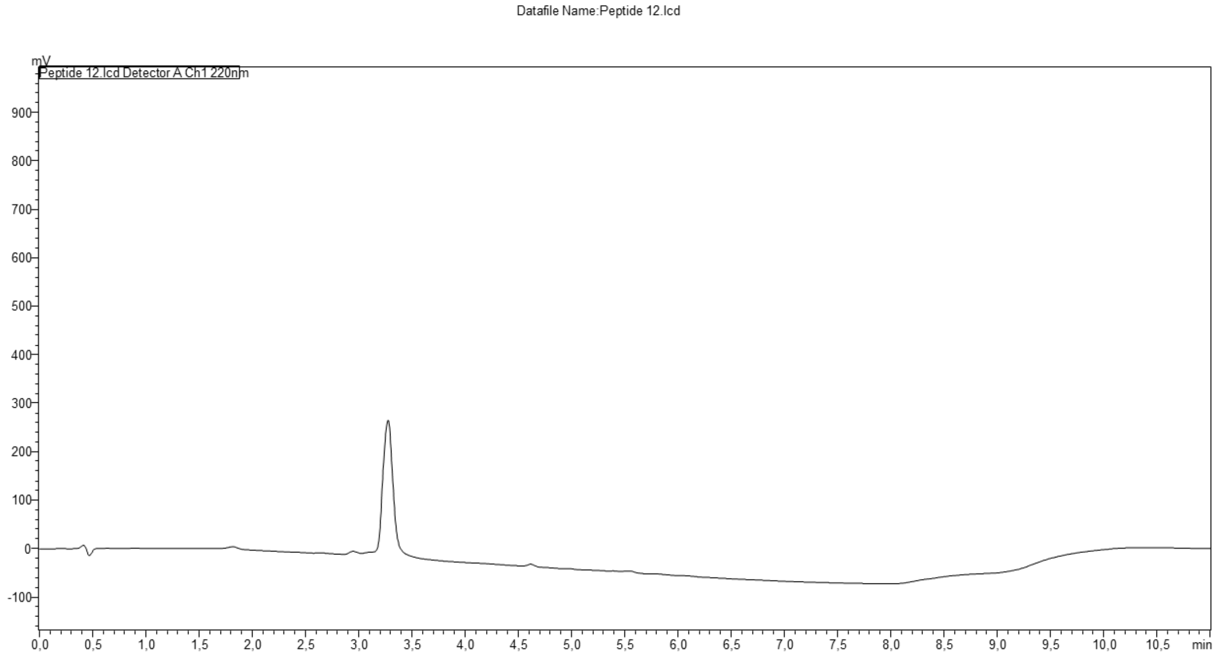

Figure S10. HR-MALDI-MS of Peptide **12** ion [M+H]$^+$ and analytical HPLC trace at 220 nm.

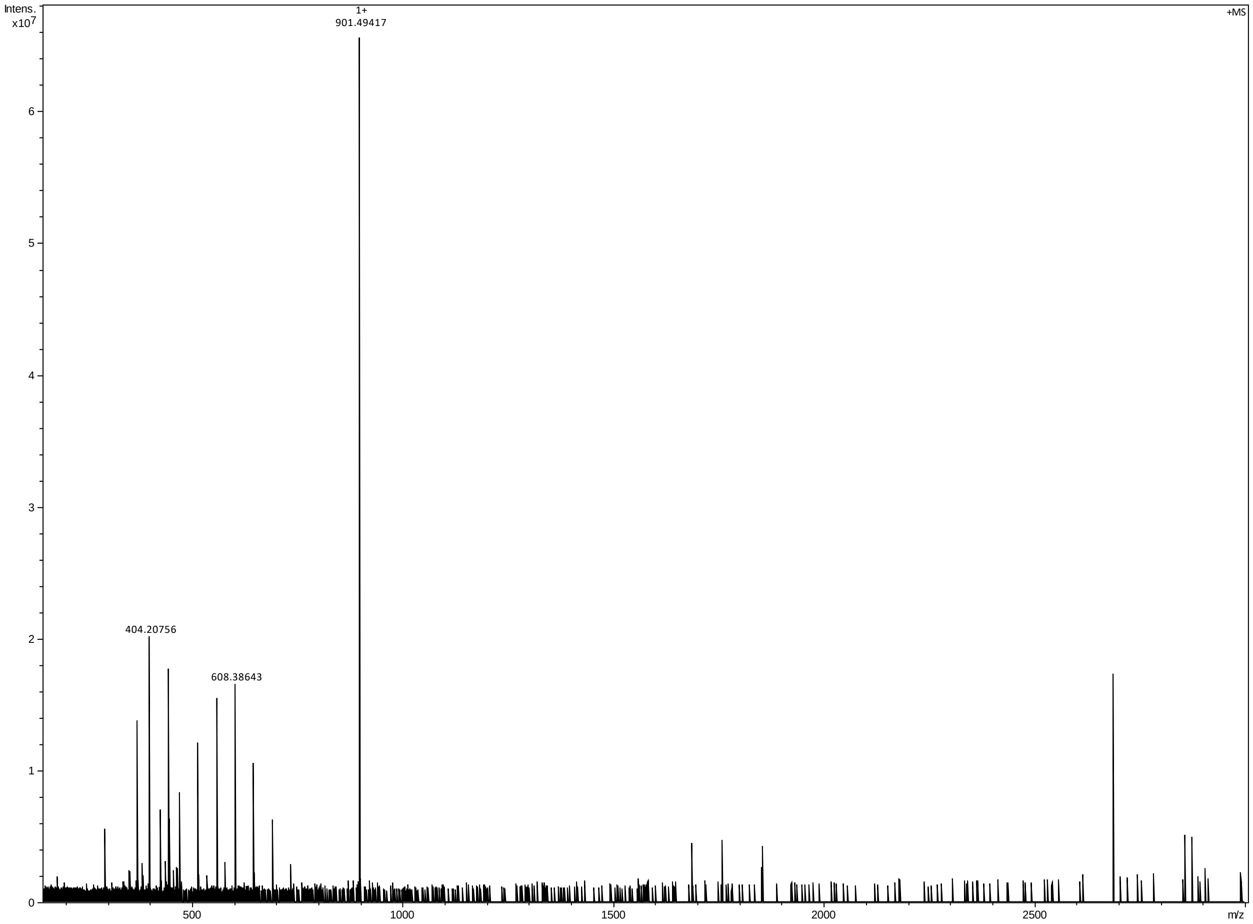

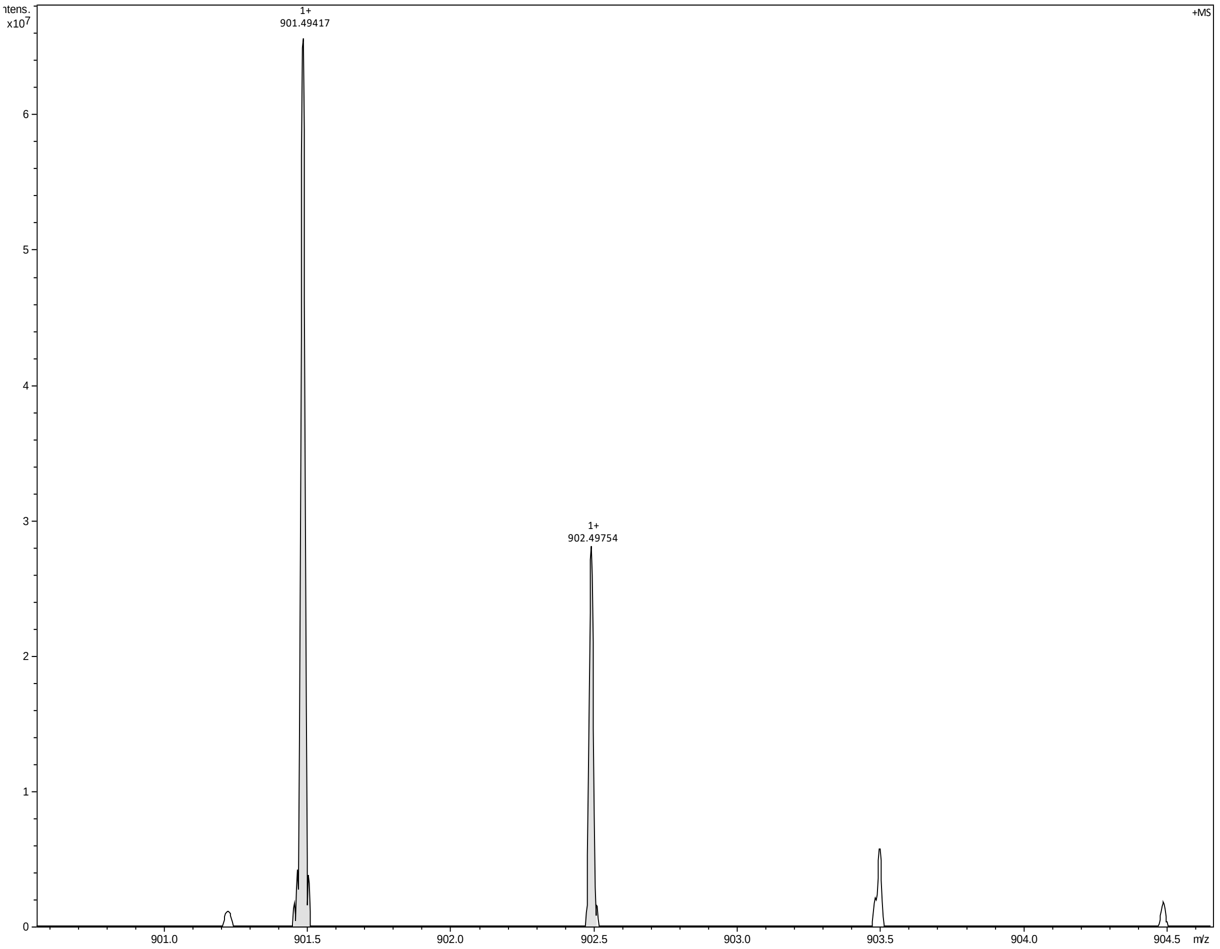

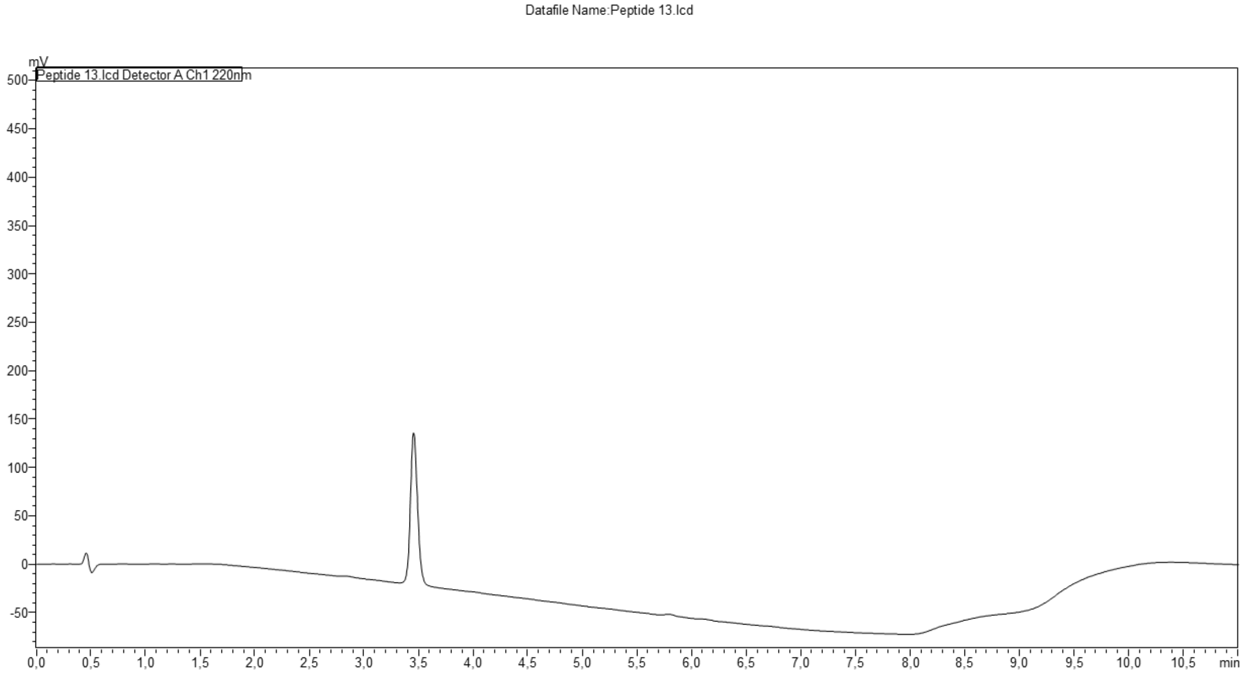

Figure S11. HR-ESI-MS of Peptide **13** ion $[M+H]^+$ and analytical HPLC trace at 220 nm.

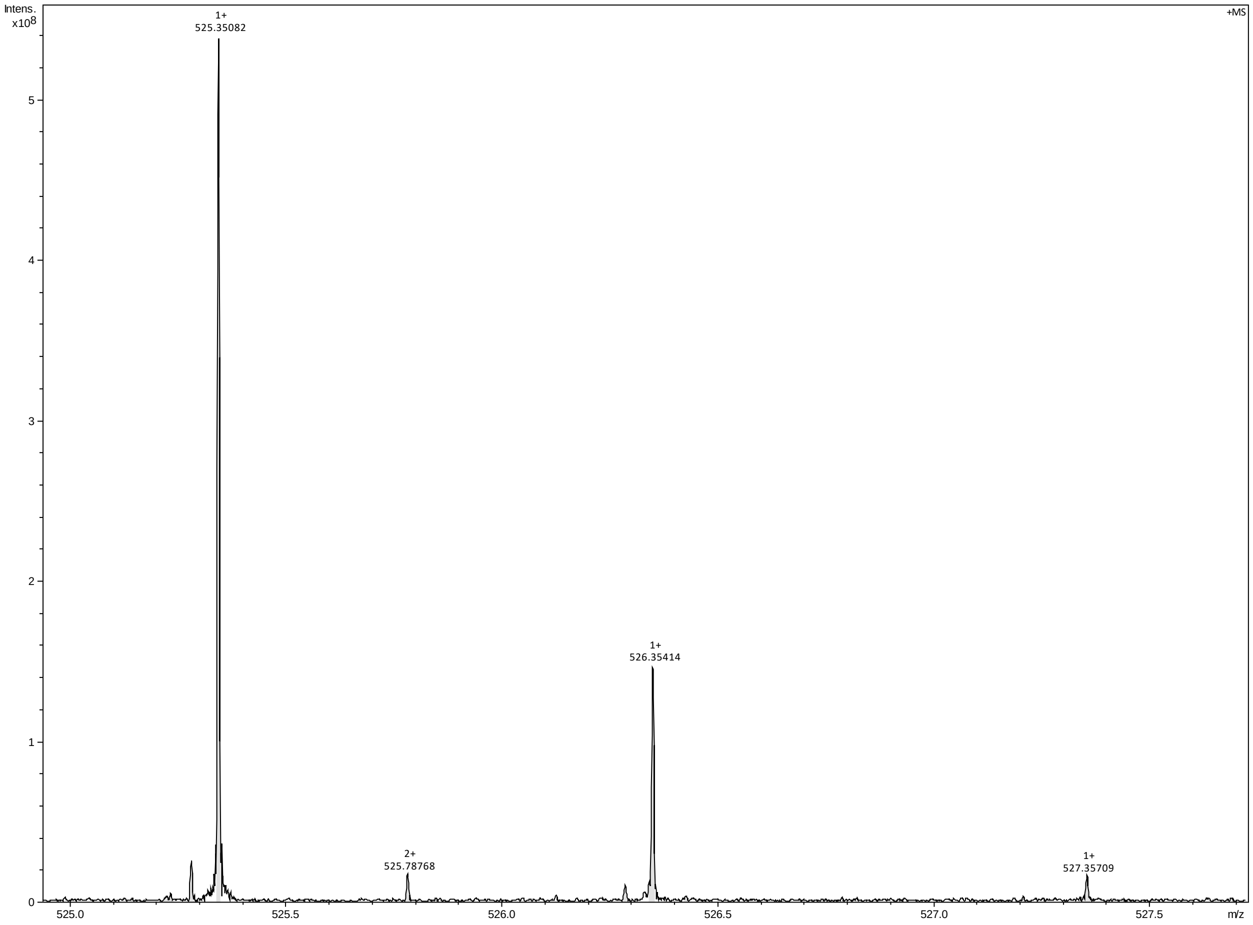

Datafile Name:Peptide 14.lcd

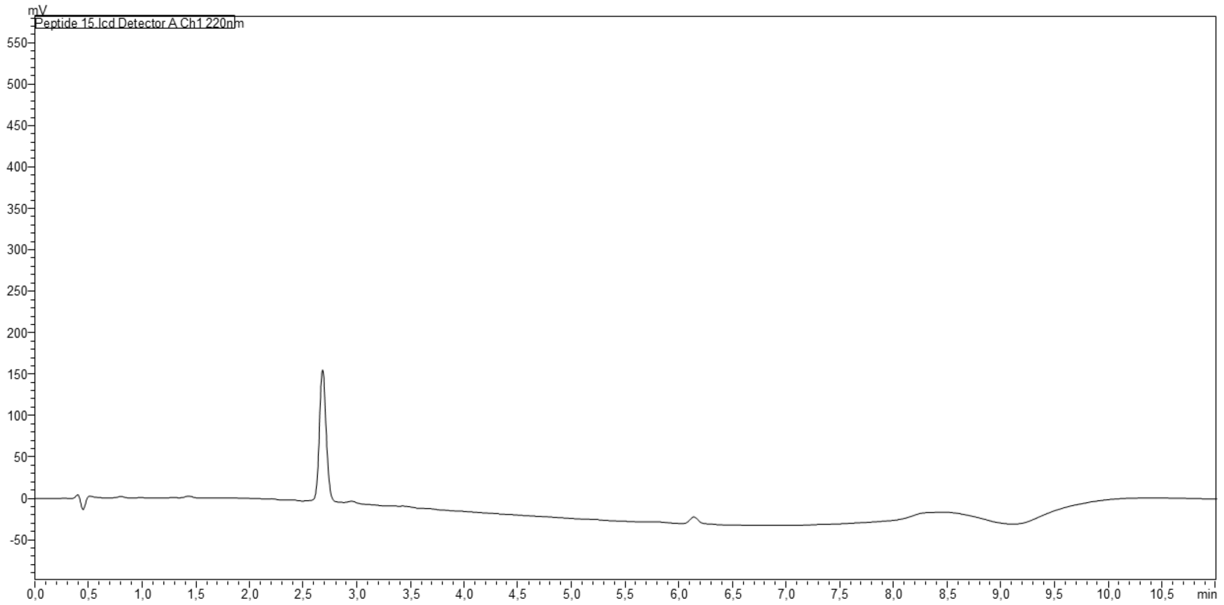

Figure S12. HR-ESI-MS of Peptide **14** ion $[M+H]^+$ and analytical HPLC trace at 220 nm.

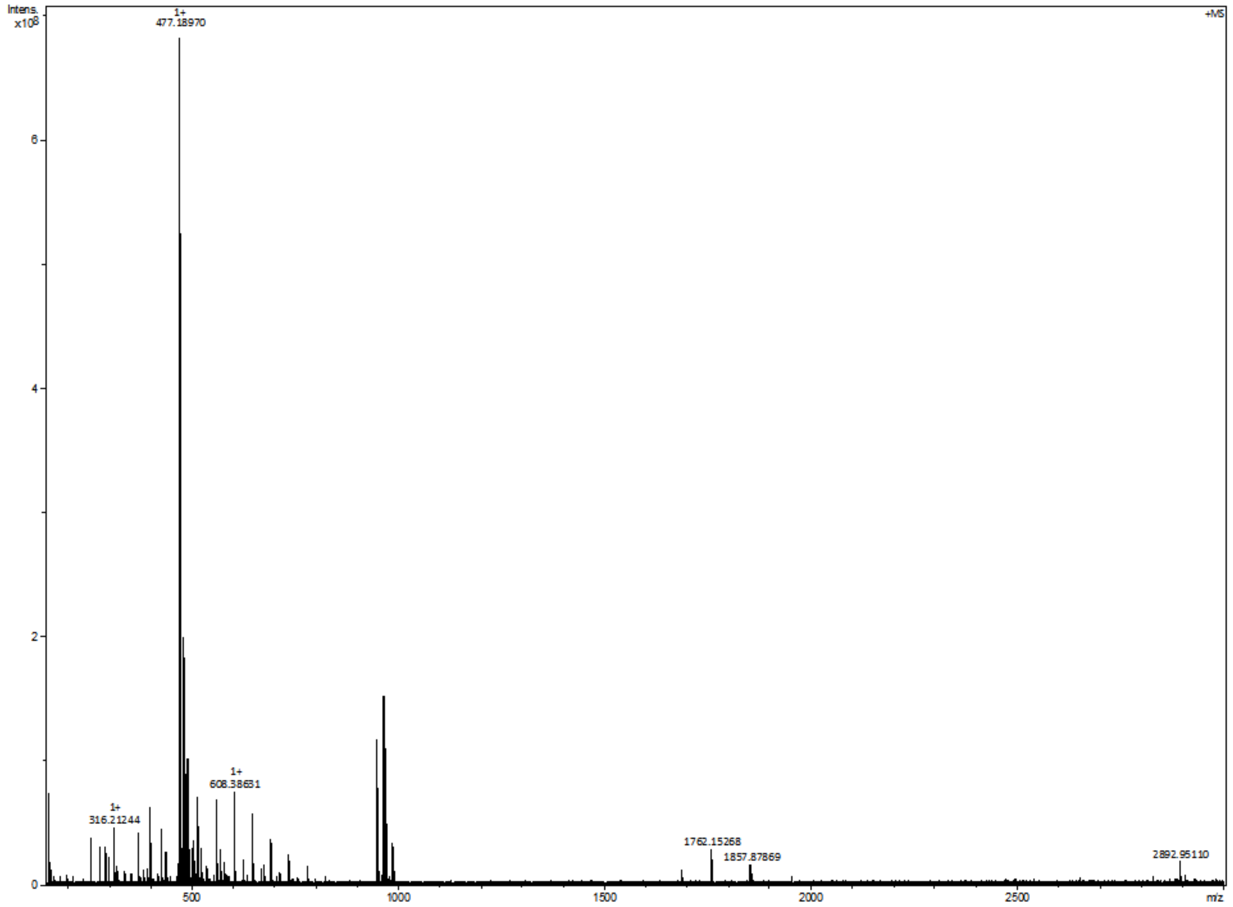
Intens.
x10^8
+MS
1+
477.18970
1+
316.21244
1+
608.38631
1762.15268
1857.87869
2892.95110
m/z

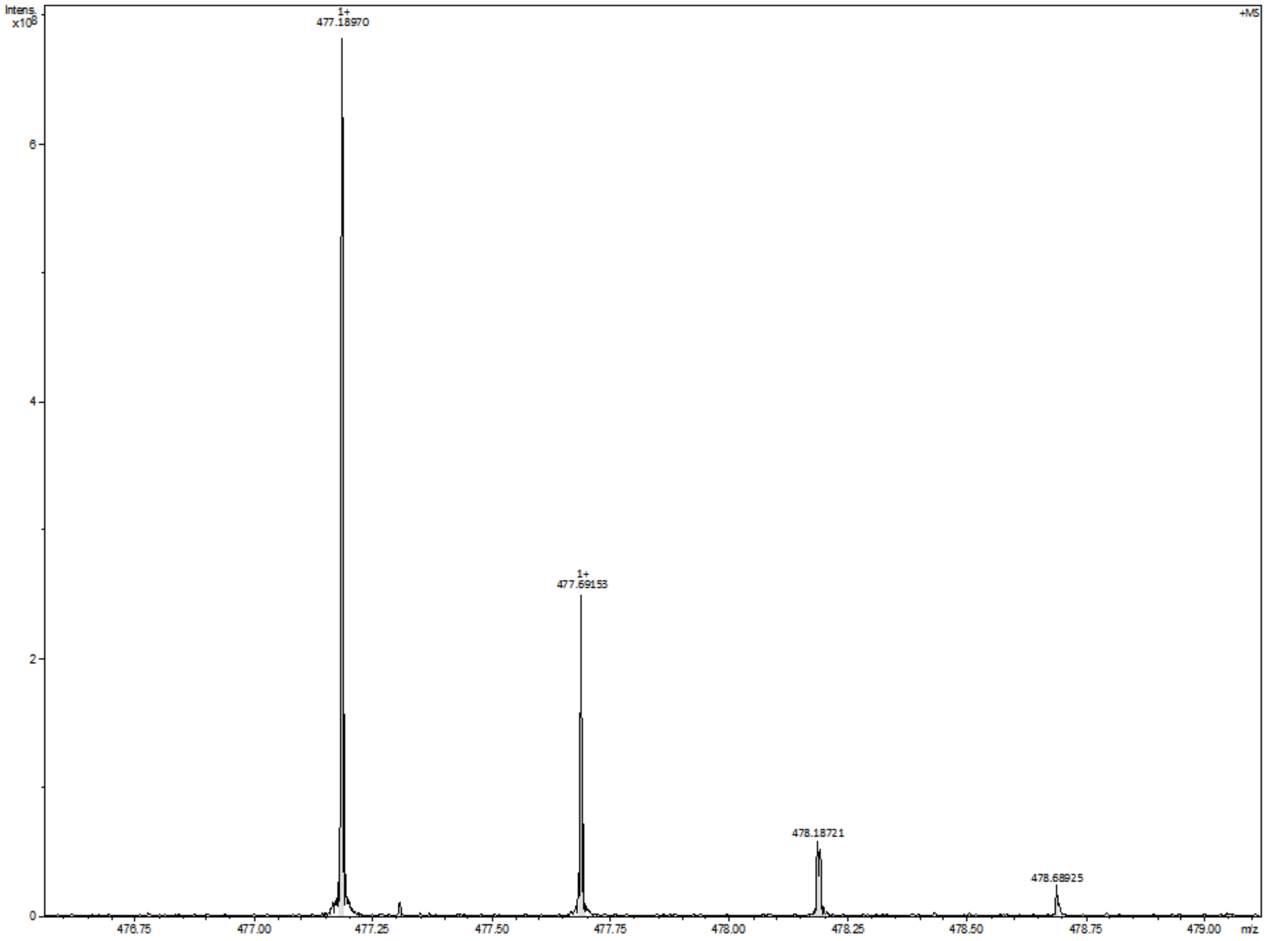
Intens.
x10^8
+MS
1+
477.18970
1+
477.69153
478.18721
478.68925
m/z

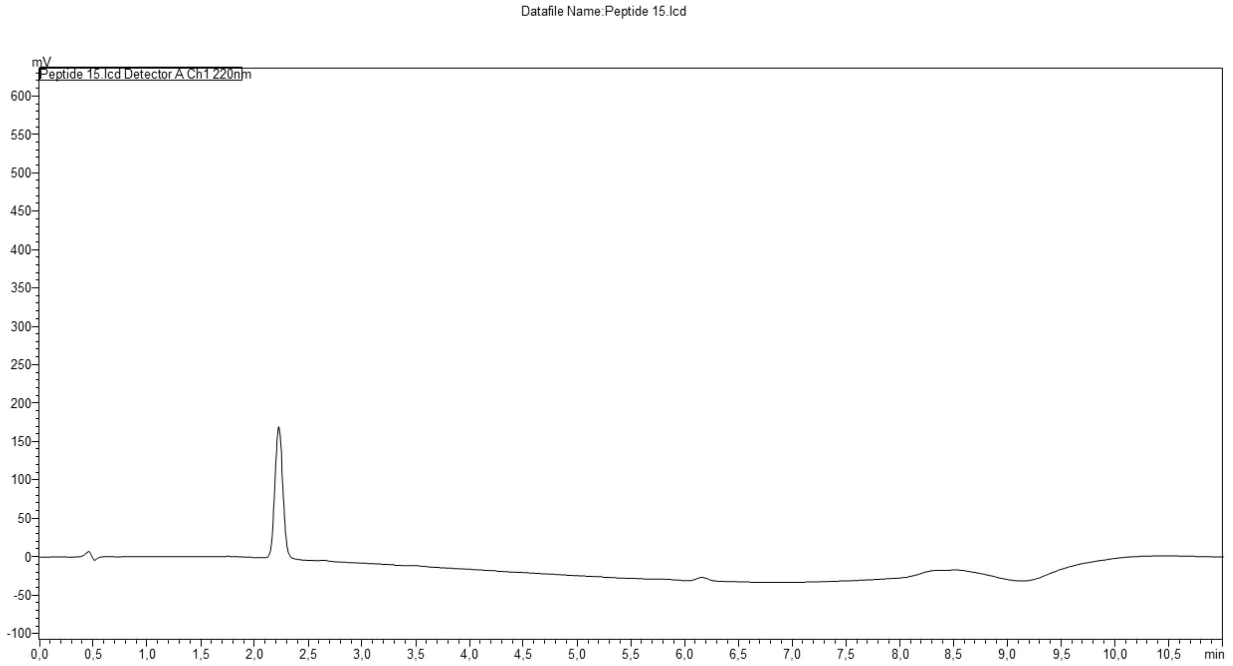

Figure S13. HR-ESI-MS of Peptide **15** ion $[M+H]^+$ and analytical HPLC trace at 220 nm.

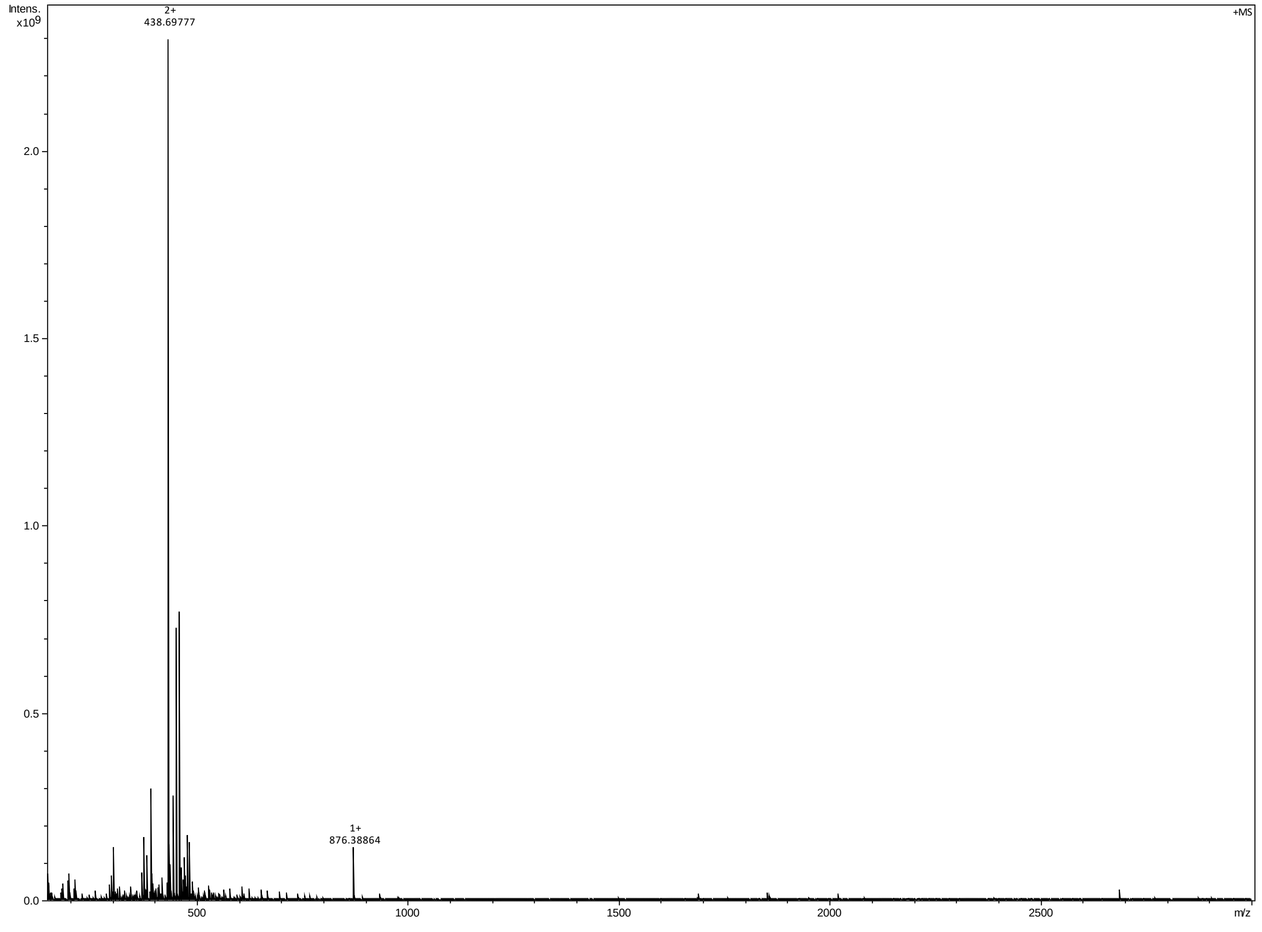

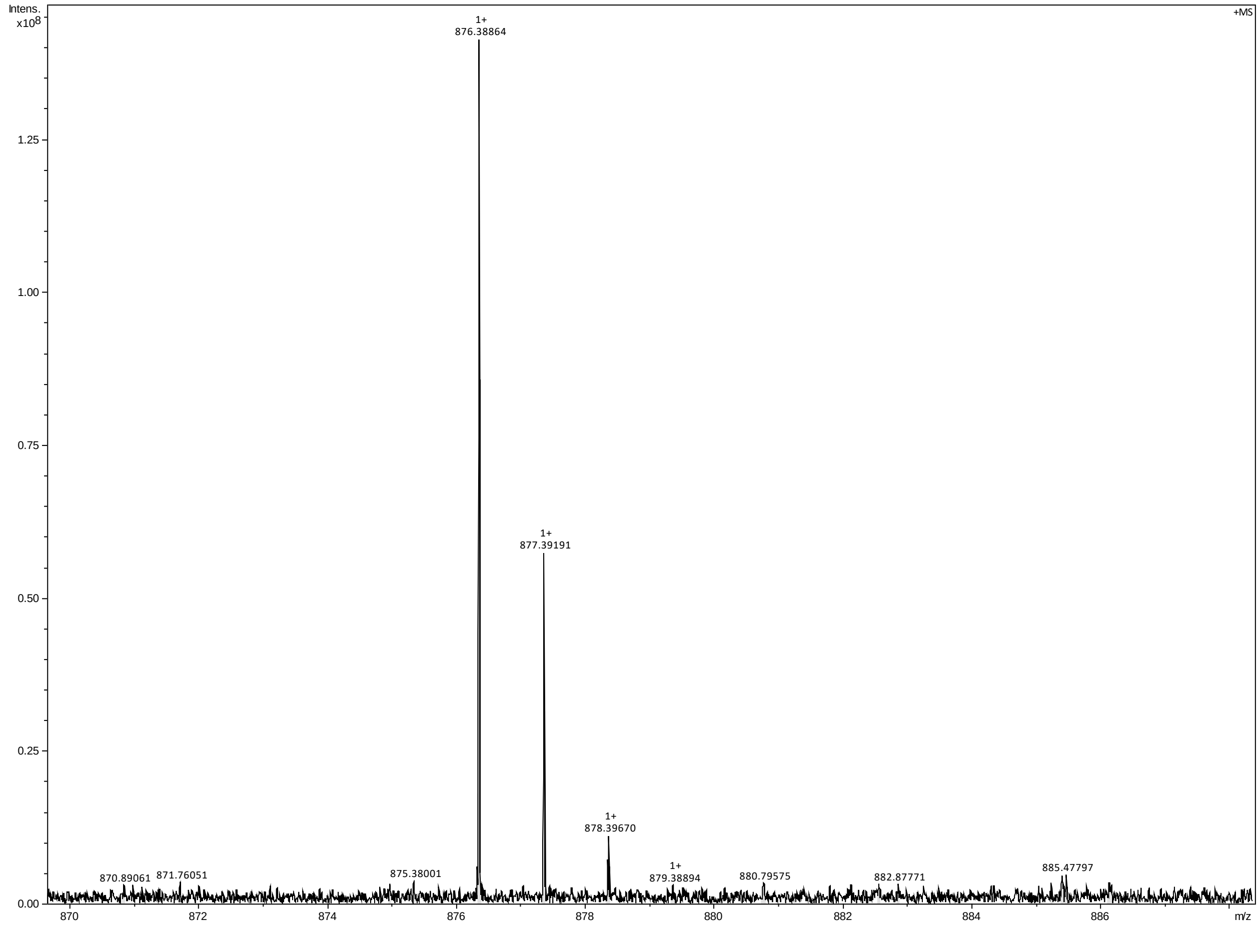

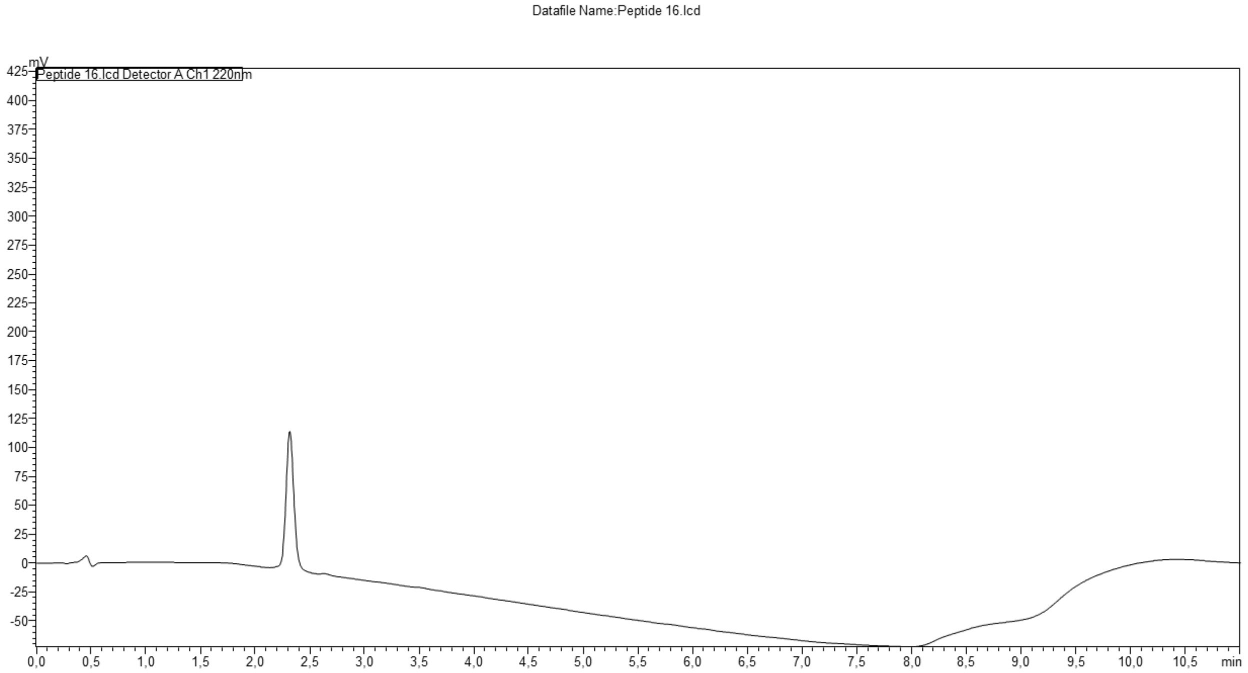

Figure S14. HR-ESI-MS of Peptide **16** ion $[M+H]^+$ and analytical HPLC trace at 220 nm.

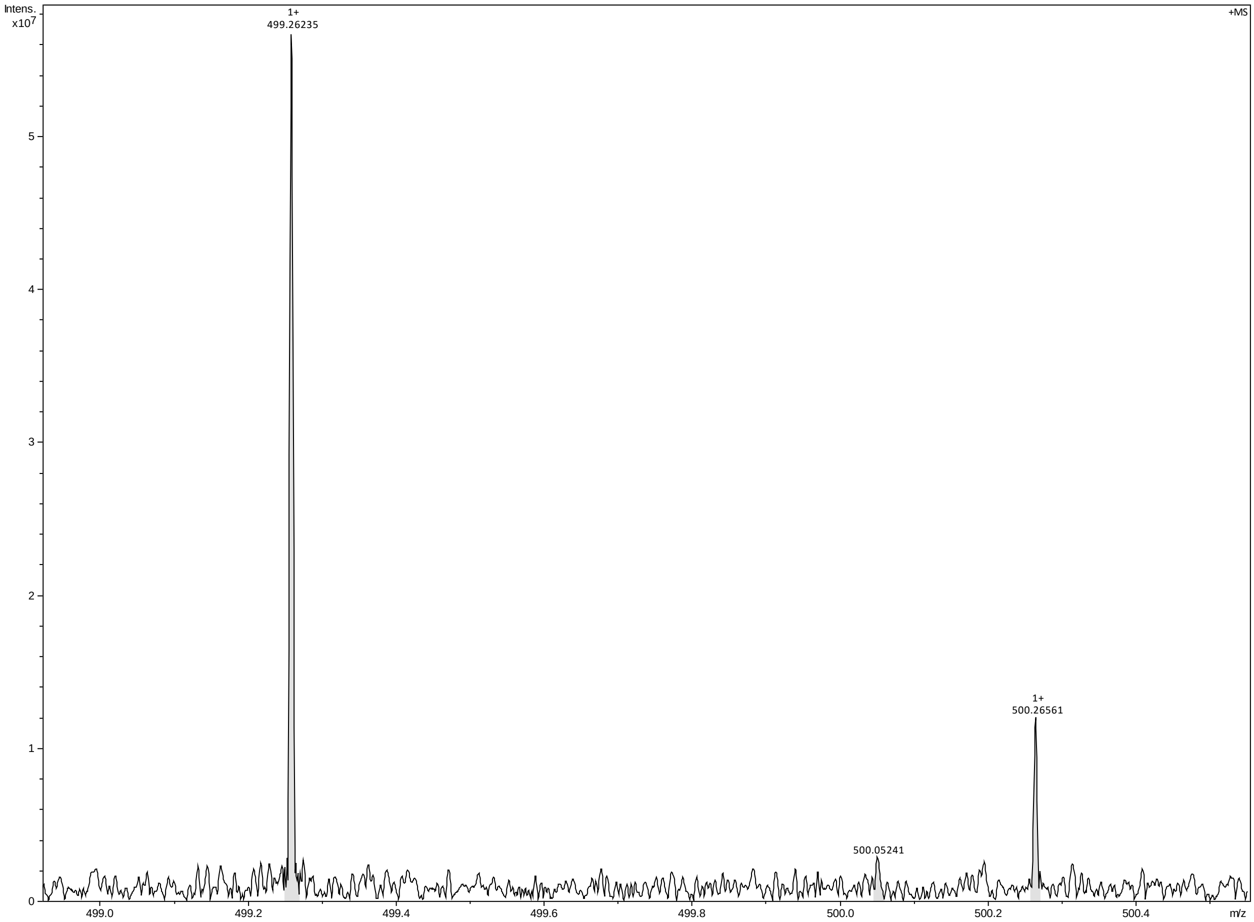

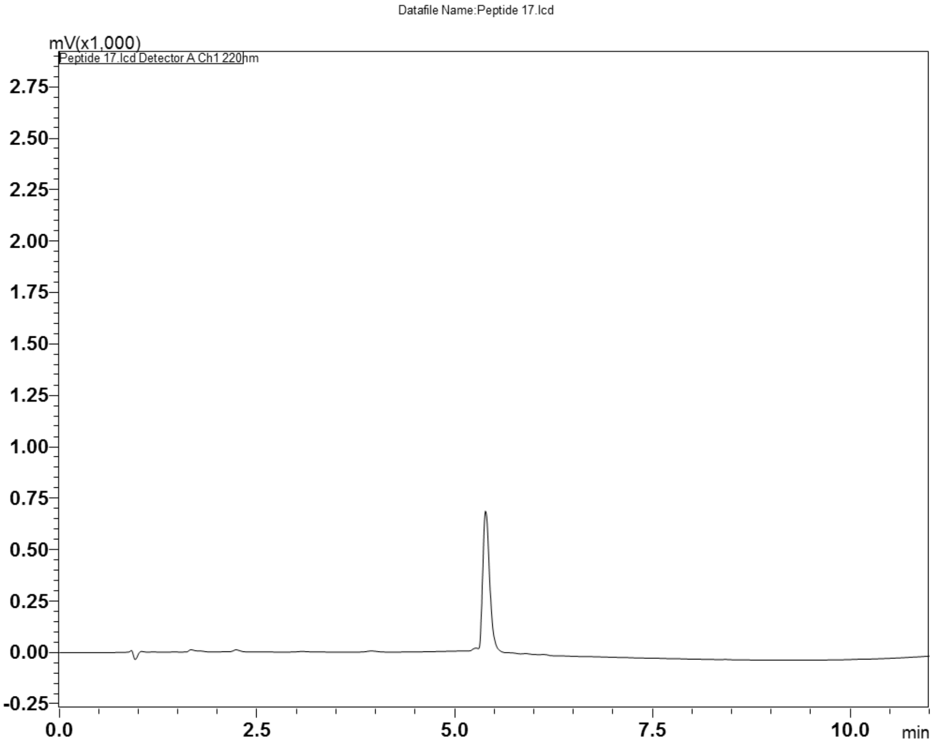

Figure S15. HR-ESI-MS of Peptide **17** ion $[M+H]^{+}$ and analytical HPLC trace at 220 nm.

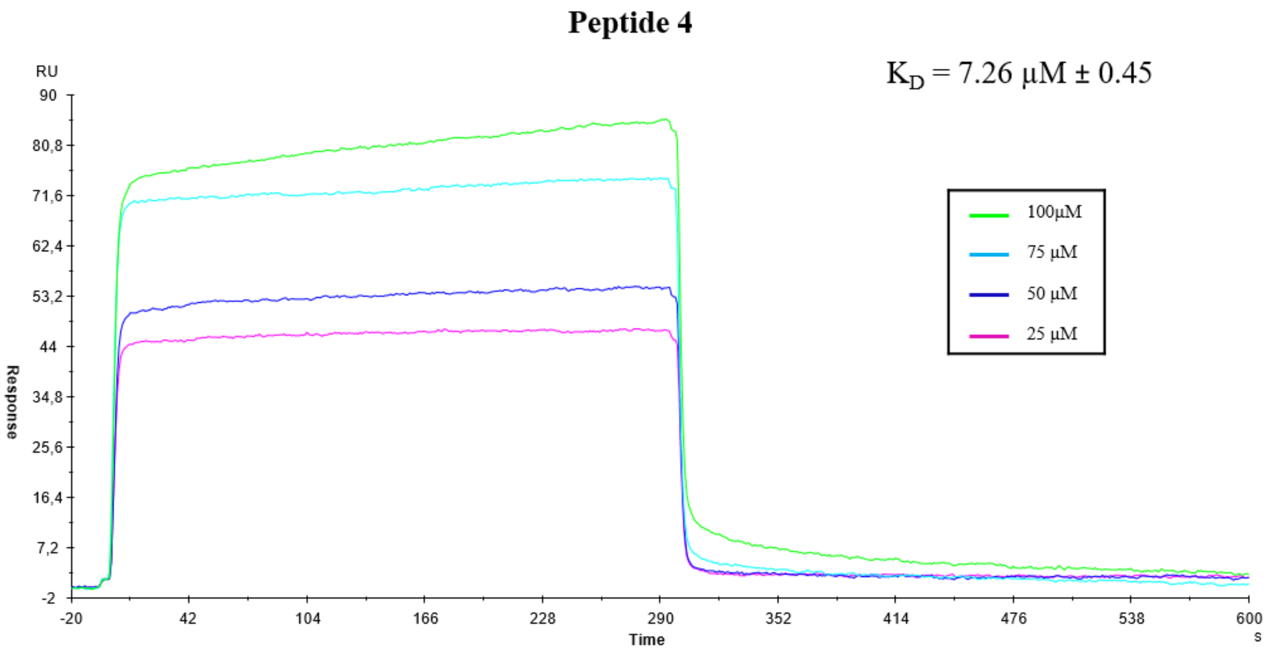

Figure S16. Sensorgram obtained from SPR interaction analysis of compound **4** binding to immobilized HA. This compound was injected at four different concentrations (from 25 µM to 100 µM).

Table S2. ^{1}H chemical shifts (ppm) of **1** in HFA/H_2O (600 MHz, 300 K).

residue	NH	αH	βH	γH	δH	εH	Others
Ser1	7.83	4.12	3.63	-	-	-	-
Lys2	7.45	3.96	1.47	1.12	1.34	2.65	-
			1.43	1.07			
His3	7.87	4.37	2.95	-	6.89 (2H)	-	-
			2.84		8.08 (4H)	-	-
Ser4	7.80	4.22	3.66	-	-	-	-
			3.54	-	-	-	-
Ser5	7.89	4.12	3.61				
Leu6	7.35	4.06	1.29	1.22	0.61	-	-
					0.51	-	-
Asp7	7.59	4.14	2.43	-	-	-	-
Cys8	7.40	4.06	2.69	-	-	-	-
			2.65				
Val9	7.25	3.76	1.81	0.61	-	-	-
Leu10	7.31	3.99	1.30	1.22	0.53	-	-
					0.51		
Arg11	7.21	4.33	1.53	1.54	2.85		6.609 (NHE)
			1.44		2.77		7.526 (NHZ)
Pro12	-	4.04	1.93	1.61	3.39	-	-
			1.71		3.25		

Table S3. ^{1}H chemical shifts (ppm) of **13** in HFA/H_2O (600 MHz, 300 K).

residue	NH	αH	βH	γH	δH	εH	Others
Ser1	7.48	4.28	3.78	-	-	-	
Leu2	8.11	4.06	1.37	1.27	0.60<br>0.64		
Asp3	7.98	4.19	2.42				
Cys4	7.54	4.18	2.69				
Val5	7.41	3.82	1.87	0.66			
Leu6	7.37	4.06	1.28	1.37	0.57<br>0.61		
Arg7	7.37	4.37	1.47<br>1.57	1.36	2.89	-	6.71(NHE)
Pro8	-	4.08	1.76<br>1.99	1.67	3.31<br>3.44	-	-

Table S4. ^{1}H chemical shifts (ppm) of **14** in [D_6]DMSO (600 MHz, 300 K).

residue	NH	αH	βH	γH	δH	εH	Others
Acetyl	-	-	-	-	-	-	1.88
Val1	7.94	4.12	1.97	0.84	-	-	-
Leu2	8.01	4.31	1.48<br>1.59	1.45	0.82<br>0.87	-	-
Arg3	7.91	4.49	1.55<br>1.77	1.53	3.09	-	7.83
Pro4	-	4.20	1.92<br>2.03	1.79	3.59<br>3.73	-	6.88<br>7.32

Table S5. ^{1}H chemical shifts (ppm) of **15** in [D$_6$]DMSO (600 MHz, 300 K).

residue	NH	αH	βH	γH	δH	εH	Others
Acetyl	-	-	-	-	-	-	1.88
Ser1	7.95	4.30	3.54<br>3.57				
Leu2	8.03	4.27	1.62	1.49	0.85<br>0.88		
Asp3	8.20	4.52	2.53<br>2.77				
Cys4	7.75	4.28	2.76<br>2.83				7.24

Table S6. ^{1}H chemical shifts (ppm) of **17** in [D$_6$]DMSO (600 MHz, 300 K).

residue	NH	αH	βH	γH	δH	εH	Others
Acetyl	-	-	-	-	-	-	1.87
Ser1	8.12	4.33	3.62<br>3.57	-	-	-	-
Lys2	8.34	4.17	1.65<br>1.51	1.27	1.51	2.73	-
His3	8.06	4.41	2.96<br>2.85	-	6.82 (2H)	7.51 (4H)	-
Ser4	7.86	4.14	3.63	-	-	-	-

Table S7. Mean values of φ, ψ and χ1 angles and αC distances relative to the most representative conformers of **1**.

peptide	sequence	i+1 φ	i+1 ψ	i+1 χ1	i+2 φ	i+2 ψ	i+2 χ1	αC distance i to i+2	αC distance i to i+3
**1**	Lys2- Ser5	-52.9	-29.7	-83.7	-138.1	-42.8	-140.3	-	4.8
	Ser4-Asp7	-76.3	166.1	-124.7	69.9	8.5	-67.0	-	6.2
	Leu6-Val9	-108.6	75.2	-95.6	64.6	27.2	-120.6	-	5.7
	Asp7-Leu10	64.6	27.2	-120.6	71.1	42.9	-164.2	-	5.3
	Val9-Arg11	-76.1	91.0	-147.5	-	-	-	5.9	-

Table S8. Mean values of φ, ψ and χ1 angles and αC distances relative to the most representative conformers of **13**.

		i+1			i+2			αC distance
peptide	sequence	φ	ψ	χ1	φ	ψ	χ1	i to i+3
**13**	Cys4-Arg7	-51.9	-29.7	-164.4	-152.9	20.8	-116.0	4.4

Table S9. ^{1}H chemical shifts (ppm) of **4** in HFA/H_2O (600 MHz, 300 K).

residue	NH	αH	βH	γH	δH	εH	Others
Lys1		3.74	1.67	2.76	1.21		
Ala2	8.22	4.01	1.15				
Asn3	7.89	4.34	2.53		6.34<br>6.98		
Glu4	8.01	3.95	1.71<br>1.79	2.05			
Gly5	7.92	3.65					
Leu6	7.45	4.04	1.37	1.30	0.60		
Thr7	7.54	3.95		0.90			
Trp8	7.70	4.22	3.05				6.85<br>7.17<br>7.29<br>6.93<br>9.35
Asn9	7.63	4.10	2.31<br>2.36		6.27<br>6.91		
Ser10	7.63	4.05	3.64<br>3.70				
Leu11	7.45	4.56	1.42	1.28	0.58		
Lys12	7.38	3.89	1.41	1.06	1.30	2.66	
Asp13	7.66	4.41	2.45<br>2.53				
Lys14	7.25	3.92	1.49<br>1.61	1.14	1.40	2.71	

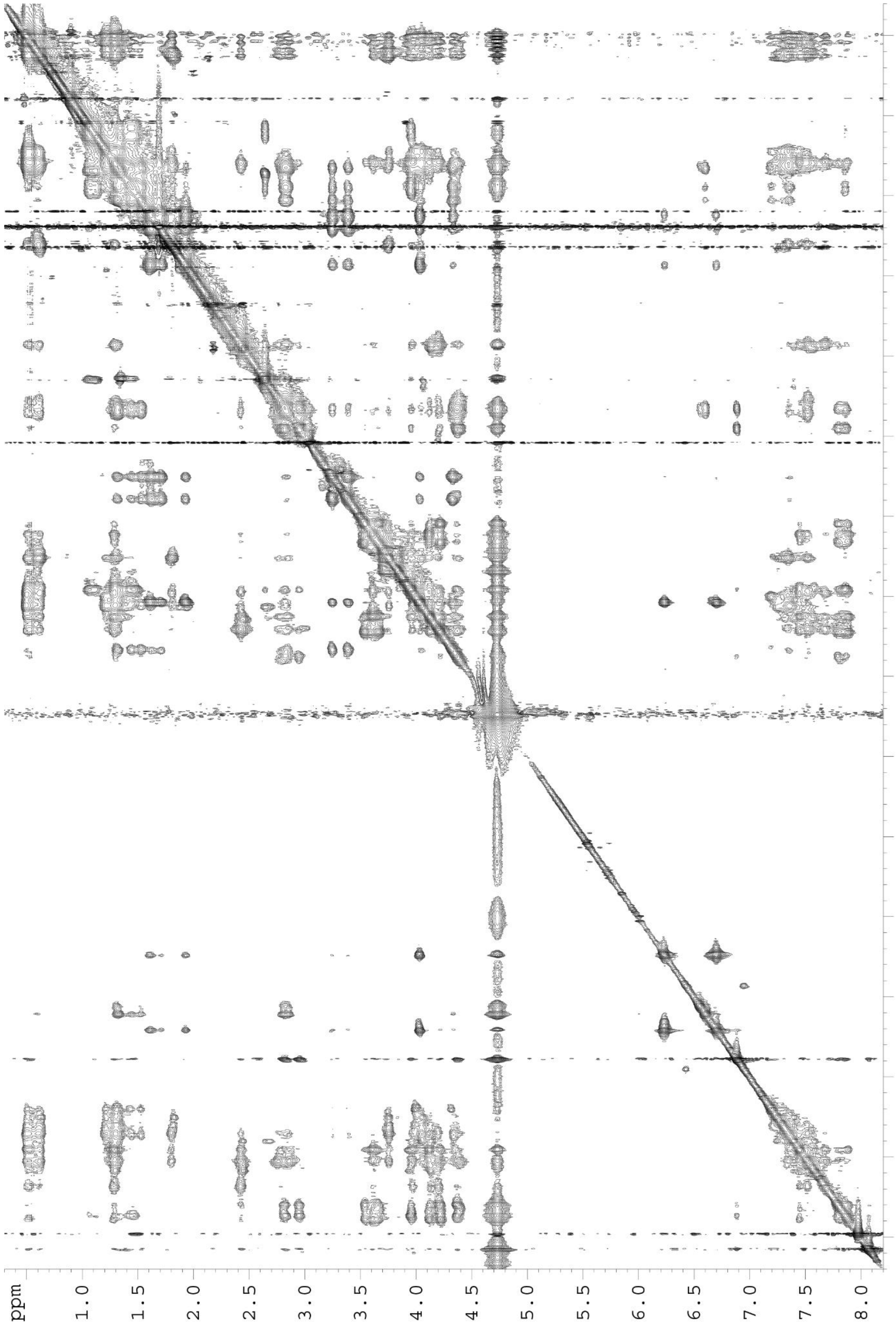

Figure S17. 2D-NOESY spectrum of **1** HFA/H_2O solution (600 MHz, 300 K, t_{mix} = 400 ms).

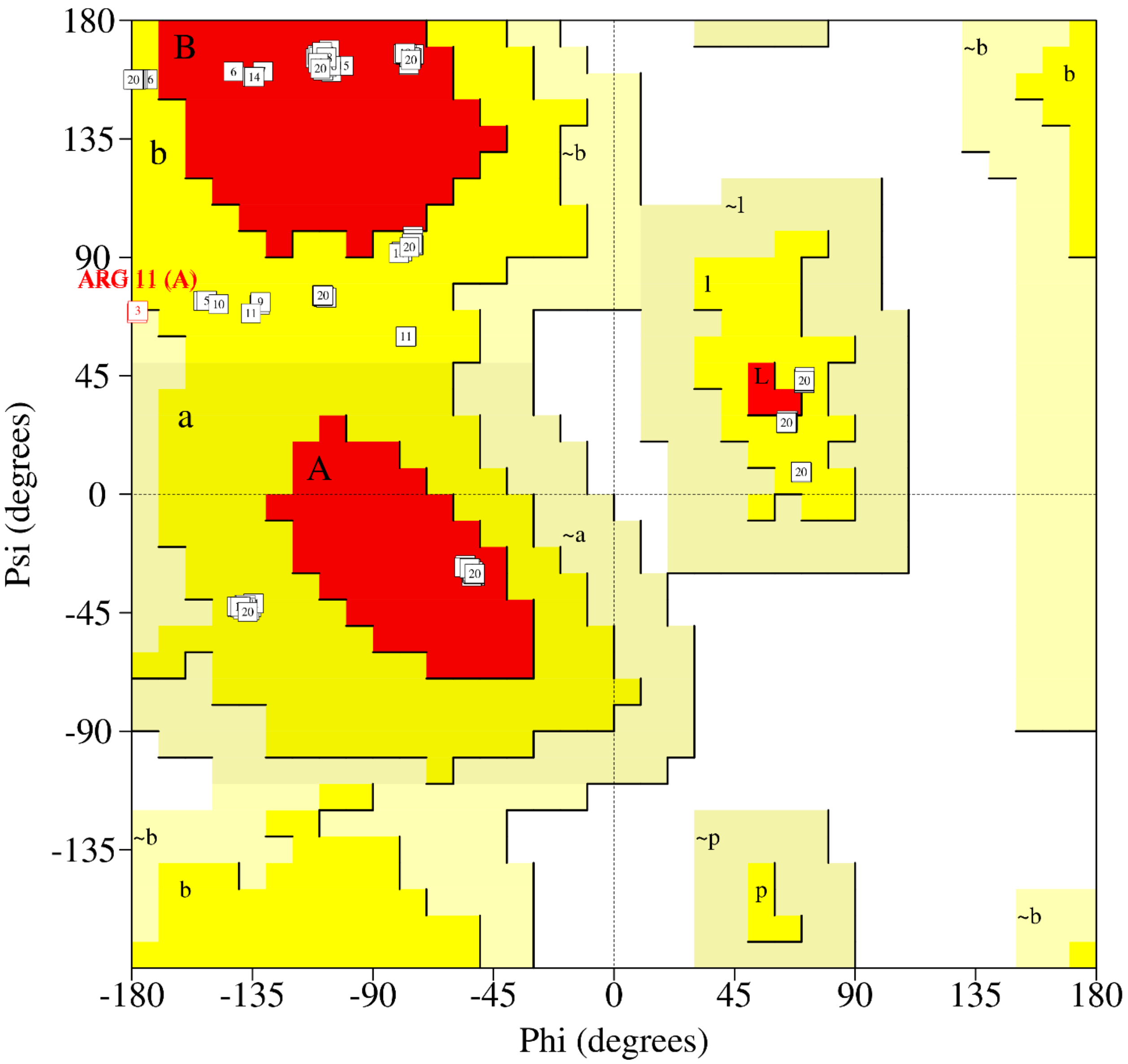

Figure S18. Ramachandran plot of NMR derived bundle of **1**, calculated by PROCHECK[i] software.

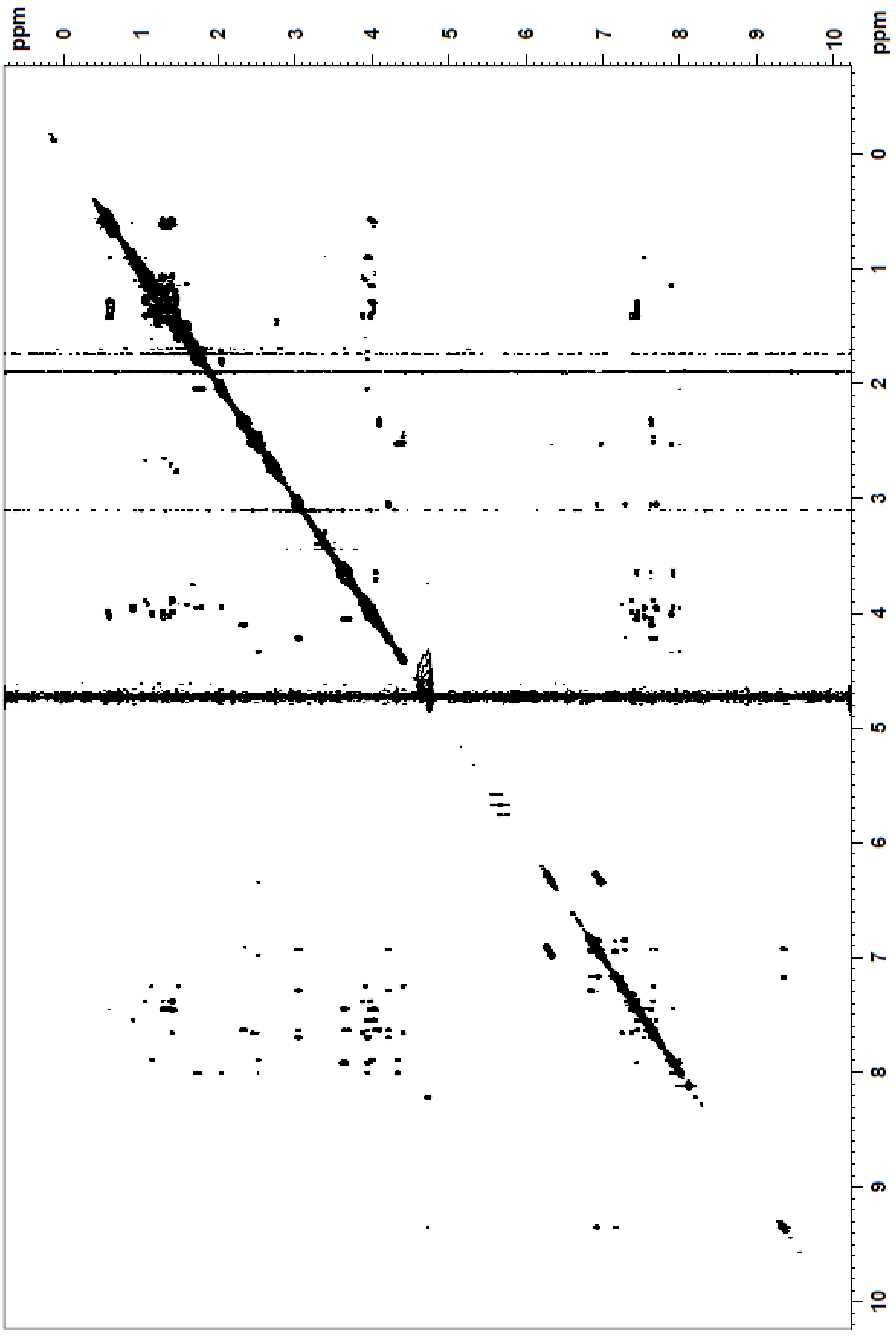

Figure S19. 2D-NOESY spectrum of **4** HFA/H_2O solution (600 MHz, 300 K, t_{mix} = 300 ms).

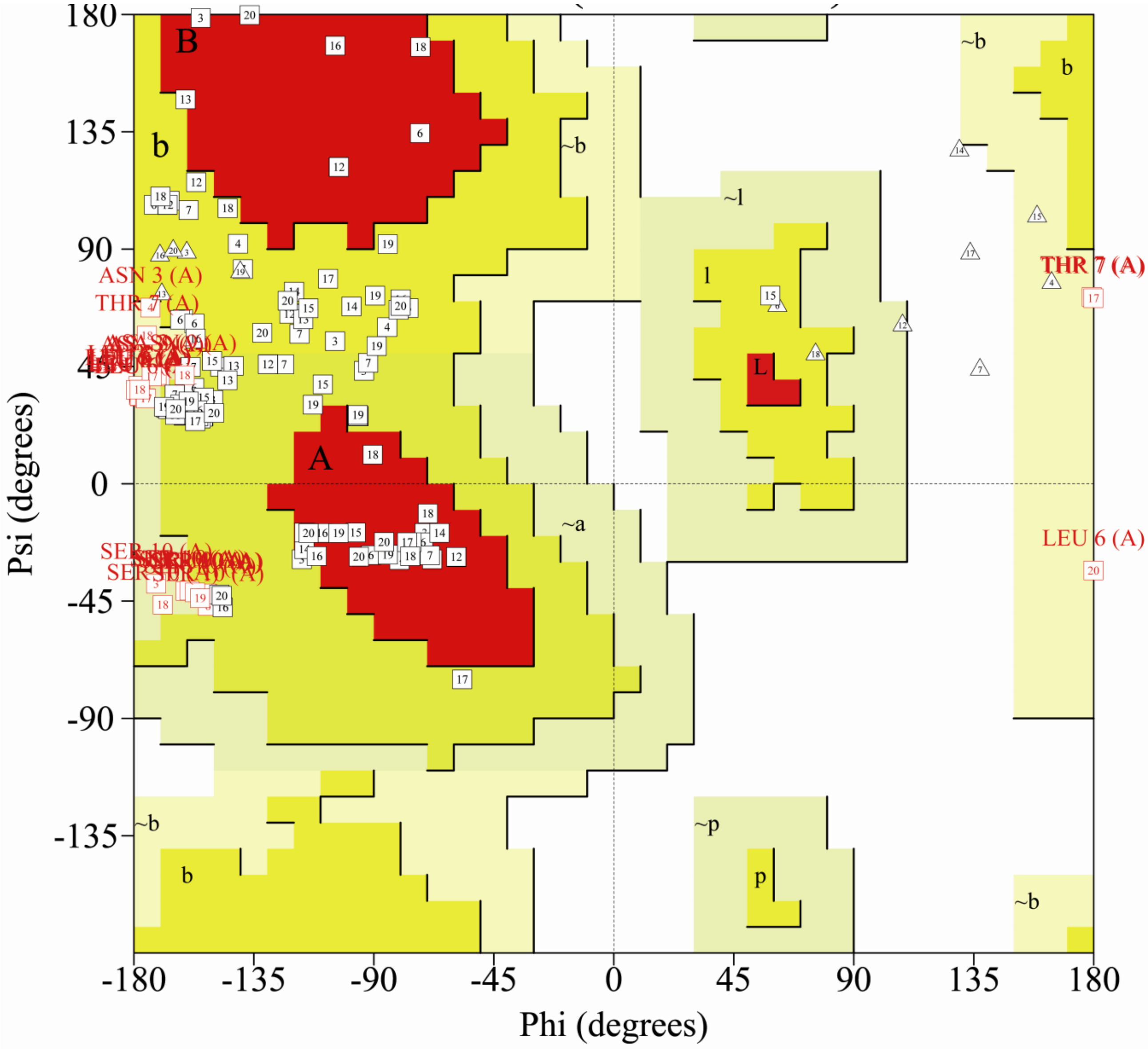

Figure S20. Ramachandran plot of NMR derived bundle of **4**, calculated by PROCHECK[i] software.

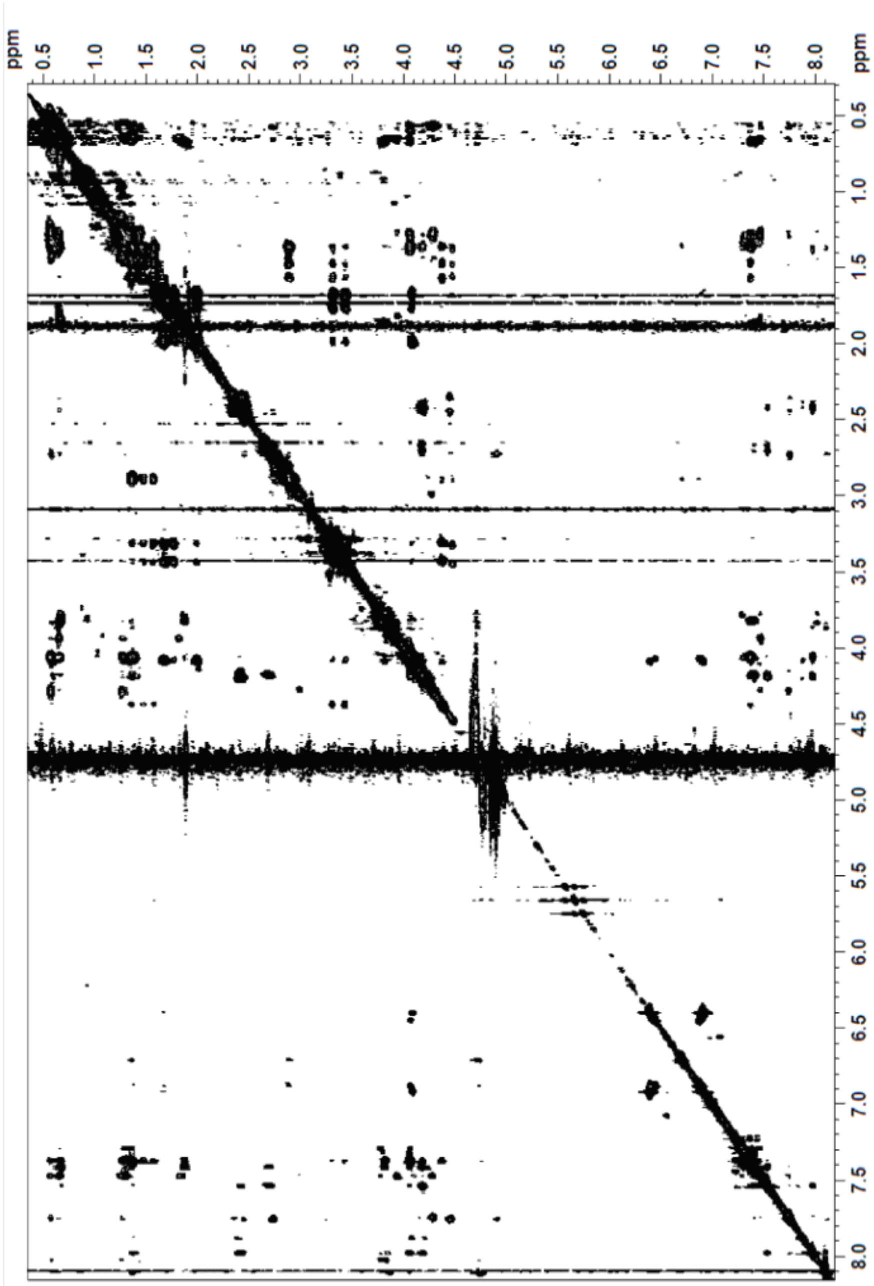

Figure S21. 2D-NOESY spectrum of **13** HFA/H_2O solution (600 MHz, 300 K, t_{mix} = 500 ms).

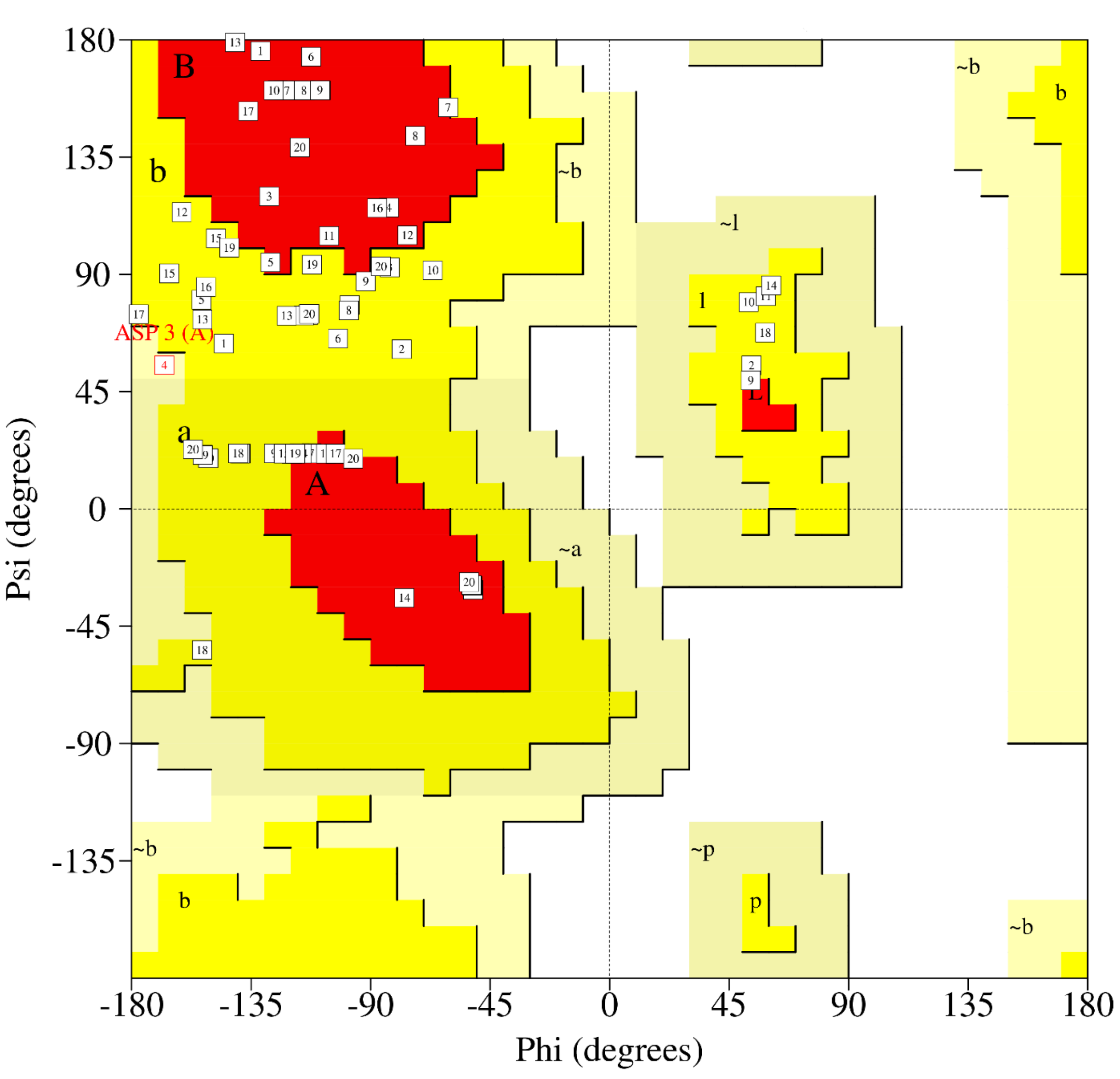

Figure S22. Ramachandran plot of NMR derived bundle of **13**, calculated by PROCHECK[i] software.

References

[i] Laskowski, R. A., Rullmannn, J. A., MacArthur, M. W., Kaptein, R., Thornton, J. M. AQUA and PROCHECK-NMR: programs for checking the quality of protein structures solved by NMR. *J. Biomol. NMR.* **8**, 477-486 (1996).
